# De novo *ZIC2* frameshift variant associated with frontonasal dysplasia in a Limousin calf

**DOI:** 10.1186/s12864-020-07350-y

**Published:** 2021-01-02

**Authors:** Marina Braun, Annika Lehmbecker, Deborah Eikelberg, Maren Hellige, Andreas Beineke, Julia Metzger, Ottmar Distl

**Affiliations:** 1 Institute for Animal Breeding and Genetics, University of Veterinary Medicine Hannover, 30559 Hannover, Germany; 2 Department for Pathology, University of Veterinary Medicine Hannover, 30559 Hannover, Germany; 3 Clinic for Horses, University of Veterinary Medicine Hannover, 30559 Hannover, Germany

**Keywords:** Cattle, Stillborn: arhinencephaly, Aprosencephaly, de novo mutation, *ZIC2*

## Abstract

**Background:**

Bovine frontonasal dysplasias like arhinencephaly, synophthalmia, cyclopia and anophthalmia are sporadic congenital facial malformations. In this study, computed tomography, necropsy, histopathological examinations and whole genome sequencing on an Illumina NextSeq500 were performed to characterize a stillborn Limousin calf with frontonasal dysplasia. In order to identify private genetic and structural variants, we screened whole genome sequencing data of the affected calf and unaffected relatives including parents, a maternal and paternal halfsibling.

**Results:**

The stillborn calf exhibited severe craniofacial malformations. Nose and maxilla were absent, mandibles were upwardly curved and a median cleft palate was evident. Eyes, optic nerve and orbital cavities were not developed and the rudimentary orbita showed hypotelorism. A defect centrally in the front skull covered with a membrane extended into the intracranial cavity. Aprosencephaly affected telencephalic and diencephalic structures and cerebellum. In addition, a shortened tail was seen. Filtering whole genome sequencing data revealed a private frameshift variant within the candidate gene *ZIC2* in the affected calf. This variant was heterozygous mutant in this case and homozygous wild type in parents, half-siblings and controls.

**Conclusions:**

We found a novel *ZIC2* frameshift mutation in an aprosencephalic Limousin calf. The origin of this variant is most likely due to a de novo mutation in the germline of one parent or during very early embryonic development. To the authors’ best knowledge, this is the first identified mutation in cattle associated with bovine frontonasal dysplasia.

**Supplementary Information:**

The online version contains supplementary material available at 10.1186/s12864-020-07350-y.

## Background

Frontonasal dysplasias (FND) comprise a heterogeneous group of disorders with congenital polymalformations caused by abnormal median facial development. The absence of the olfactory tract (arhinencephaly), partial fusion (synophthalmia), complete fusion (cyclopia) or the total absence (anopthalmia) of ocular globes are phenotypes of FND found in cattle [1]. FND can also include cerebral defects as holoprosencephaly (HPE) characterized by incomplete cleavage of the prosencephalon into two hemispheres [2]. Additional organ defects like heart anomalies may occur [3].

In human and mouse, several genetic variants are associated with HPE and FND. Besides chromosomal defects, critical FND candidate genes were identified [4–6]. In human, the spectrum of *Aristaless-Like Homeoboxprotein* (*ALX)* related FNDs involves recessively inherited loss-of-function mutations in the *ALX1*, *ALX3*, and *ALX4* genes [7]. In Burmese cats, a 12-bp in-frame deletion in the *ALX1* gene is responsible for severe craniofacial malformations and FND [8]. In ruminants, sporadic cases of different types of cyclopia and FND occur in cattle [1, 9–17], buffalo [18–20], sheep [21–23] and goats [24] (Table 1). Whole genome scans for FND were not yet performed in ruminants.

**Table 1:** Reported cases of frontonasal dysplasia (FND) in ruminants with their phenotypes

Species	Breed	Phenotype	References
Cattle	Hereford	Holoprosencephaly, arhinencephaly, hypotelorism	Cho et al. (1985) [9]
	Holstein Frisian	Cyclopia	Hammoda and Abdoud (1989) [10]
	Japanese Brown	Median cleft of the face, shortened upper jaw, ocular hypertelorism,	Moritomo et al. (1999) [11]
	German Fleckvieh	Cyclopia and arhinia	Schulze and Distl (2006) [1]
	Holstein	Cyclopia, holoprosencephaly	Kim et al. (2006) [12]
	Brown Swiss crossbreed	Cyclopia with prosencephalic aplasia, brachygnathia superior and arrhinia	ÖZcan et al. (2006) [13]
	Crossbreed	Cyclopia, absent muzzle and all the skeletal structures of the nose, protruding tongue	Malik et al. (2013) [14]
	Crossbreed	Mild hypotelorism, microphthalmia, cheiloschisis, arhinencephaly	Osman et al. (2013) [15]
	Holstein crossbreed	Synophthalmia, arrhinia	Nourani et al. (2014) [16]
	Holstein	Shortening of the nasal structures, micrognatia superior, shortened mandibles, protrusion of the tongue, bilateral eye prolapse, brain malformations	Agerholm et al. (2017) [17]
Buffalo	Surti	Shortened upper jaw, prolonged lower jaw, protruding tongue, nose divided by furrows, hydrocephalus	Pandey et al. (2010) [18]
	Murrah	Cyclopia with cleft palate and complete absence of muzzle	Singh et al. (2013) [19]
	Mediterranean River	Underdeveloped incisive, maxillary and nasal bones with a consequent tongue prolapse, and lower jaw deformities	Albarella et al. (2017) [20]
Sheep	Texel	Aprosencephaly with otocephaly	Brachthäuser et al. (2012) [21]
	Cabugi hair sheep	Domed head with a short nasal bone and curved mandible	Dantas et al. (2014) [22]
	–	Cyclopia (monkey face lamb disease)	Welch et al. (2009) [23]
Goat	Balady	Cyclopia with small upper jaw and large protruded lower jaw	Rashed et al. (2014) [24]

The objective of this study was to give a full phenotypic characterization of a sporadic FND case and filter out private genetic and structural variants for this case using whole genome sequencing (WGS) data of the affected calf, his parents and two unaffected half-siblings.

## Results

### Phenotype

**Fig. 1:**
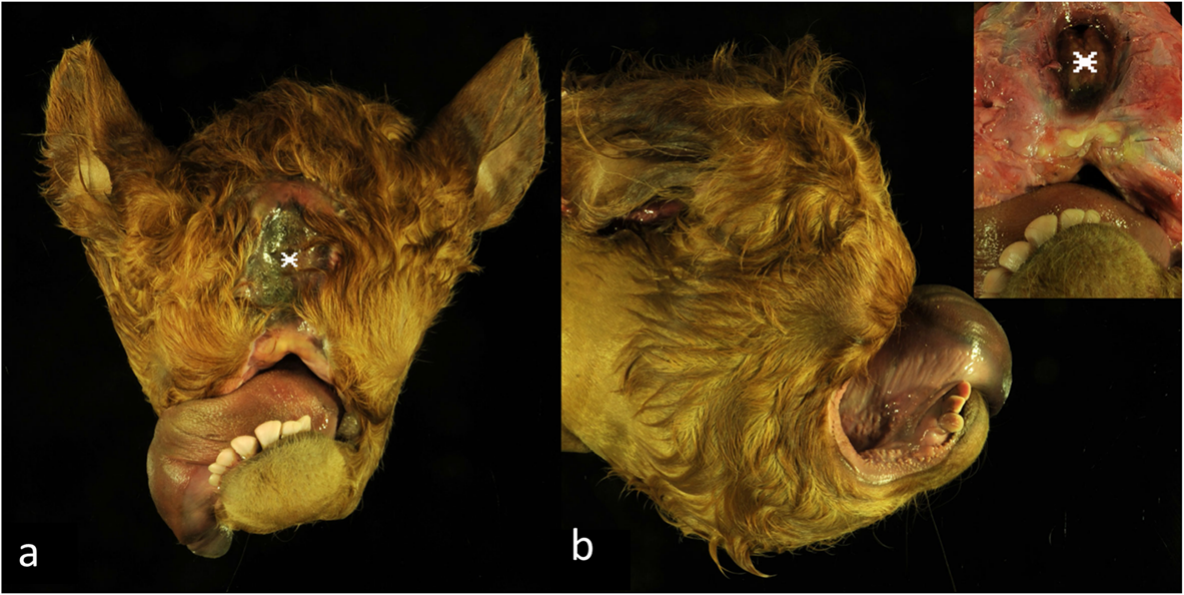
Macroscopic pictures of the skull of the affected calf (**a**, **b**) showing severe deformations and absence of facial structures. The nose and the maxilla were not developed and the mandibles were curved upwards. Eyes and orbital cavities were absent. The tongue was protruded. Centrally on the skull a defect was present (asterisk), insert showing a higher magnification of the defect after removal of the skin

The stillborn male Limousin calf had a weight of 52 kg and the crest-rump length was 103 cm (40.55 in.). The head showed severe craniofacial alterations such as abnormal development of the maxillary processes and face skull (Fig. 1). Nasal openings were missing and the region of the upper jaw and muzzle seemed to be constricted and covered with haired skin. Eyes, eyelids or eyelashes were not discernible. The tongue was protruded. A skin defect covered by a membrane was located centrally in the front of the skull. A very short tail was obvious.

### Computed tomography (CT)

**Fig. 2:**
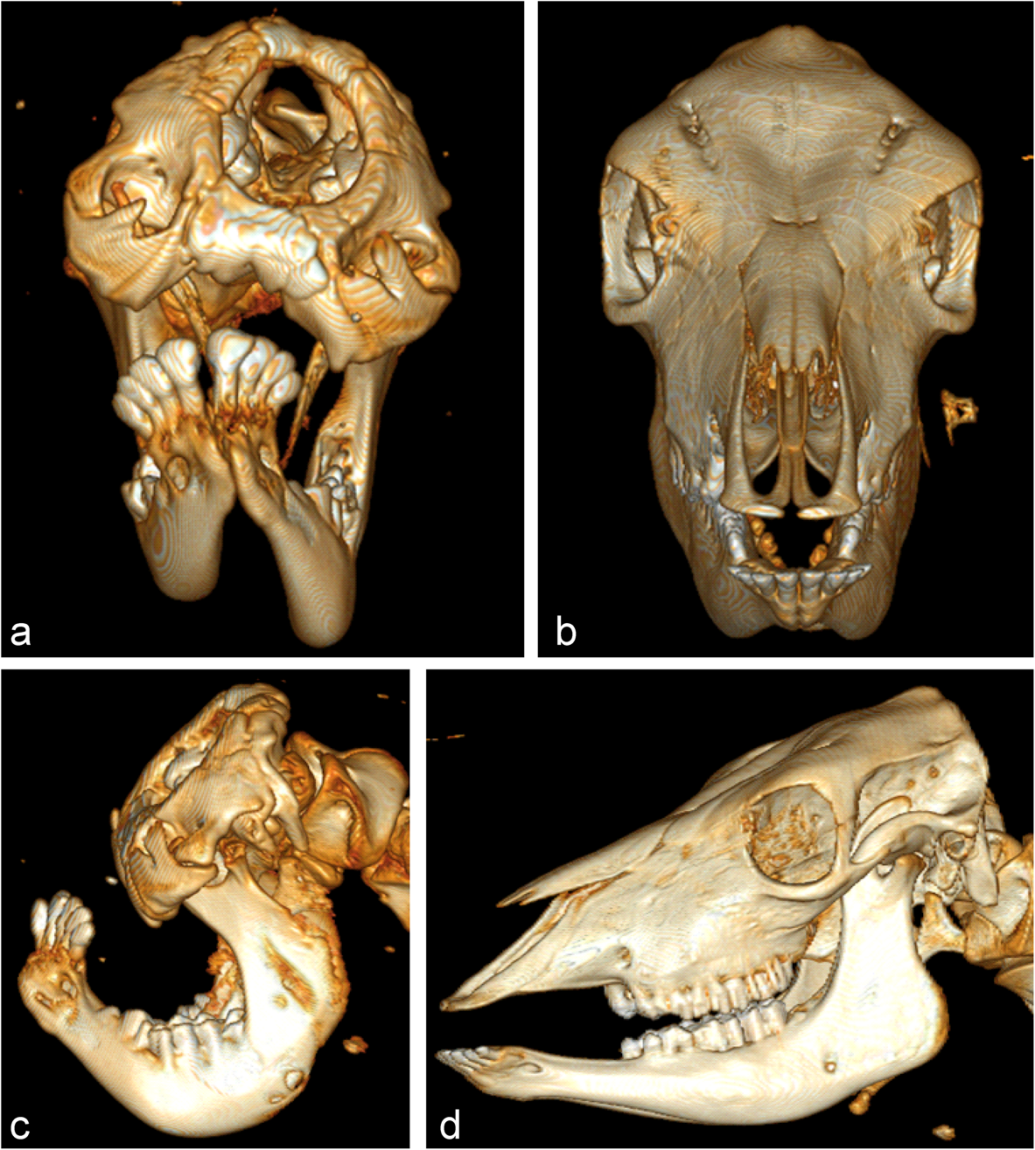
Computed tomography of the skull of the affected calf (**a**, **c**) and a control animal (**b**, **d**). The control animal was a Holstein calf. **a** In the rostrocaudal view the absence of the maxilla, nasal and incisive bones and the non-fused os frontale were striking features. Also the rudimentary orbita and the cleft palate were evident. **c** Left lateral view displays the strongly curved malformed mandible. The four pairs of incisive teeth were positioned near to the coronoid process. The molars and premolars were rudimentary curved

In the CT scan, the skull showed severe abnormalities compared to a healthy animal (Fig. 2). The maxilla was bilaterally completely absent. The mandibles were strongly curved upwards. The right part of the mandible was smaller than the left one. Four pairs of incisive teeth were completely developed, the molars and premolars were rudimentary and curved. The nasal bones, forming the nose in normal animals (*os nasale*, *os incisivum*, *os lacrimal*e, *os ethmoidale*, *os presphenoidale*, *choana* (*os palatinum*, *os spehnoidale*) and *vomer*) were absent. The *os frontale* had open structures. The orbits were rudimentary and placed on the front side near to the midline of the face instead of the lateral sides of the head. The palate had a wide and complete median cleft.

**Fig. 3:**
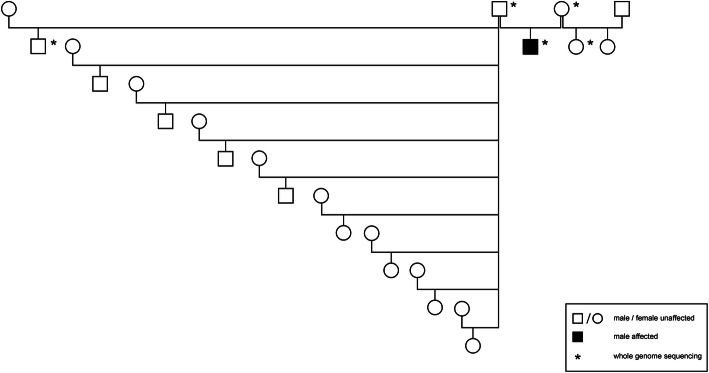
Pedigree of the Limousin calf, which was affected with a frontonasal dysplasia phenotype. Animals sampled for whole genome sequencing are marked with an asterisk

### Necropsy findings

The head showed severe deformations and absence of facial structures. The nose and the maxilla were not developed and the mandibles were curved upwards. Eyes, optic nerv and orbital cavities were absent. Centrally on the frontal skull a defect, measuring 7 × 5 × 0.5 cm in width, extended into the intracranial cavity. A thin brownish membrane covered this bone defect. The whole skull was heavily deformed and appeared shortened and squat. Only parts of the brain stem were developed. Aprosencephaly affected telencephalic (cerebral cortex, hippocampus and striatum) and diencephalic structures (globus pallidus, thalamus, hypothalamus and hypophysis) and cerebellum. The malformations of the forebrain included an absence of the prosencephalon (including telencephalon and diencephalon) and a hypoplastic mesencephalon and rhombencephalon. In the oral cavity, incisors and canini were developed, but premolars were missing. A median cleft palate of the soft and hard palate due to a complete median disclosure was evident. The vertebral column consisted of 7 cervical, 13 thoracic, 6 lumbar, 5 sacral, and only 4 coccygeal vertebral bodies.

The heart showed severe complex malformations with a persisting foramen ovale measuring 2 × 2 cm, a completely absent cardiac septum and only one single ventricle. Furthermore, a complete fetal atelectasis was found.

Histologically, the membrane covering the central defect on the skull was composed of collagen-rich connective tissue. Directly adherent to this membrane, parts of meninges and neuronal tissue were seen. Remnants of brain tissue consisted of mainly white matter with single scattered neurons.

### Pedigree analysis

The Limousin calf was the only one FND case in this herd with purebred Limousin (Fig. 3). The actual herd size when the FND case occurred was 60 females in reproductive age. The sire of the present case was a natural service purebred Limousin bull, which sired all heifers and cows of this herd for two consecutive years. An autosomal recessive mode of inheritance, where both, the dam and the sire, are heterozygous mutant, is very unlikely due to the sporadic occurrence of this case and 120 progeny within 2 years from matings of the same bull in this herd. A paternal dominant germline mutation also seems unlikely because of the large number of progeny and only one case among 120 births. A maternal germline mutation or a novel mutation in early embryonic development may be considered as possible hypotheses.

### Whole genome sequencing

**Fig. 4:**
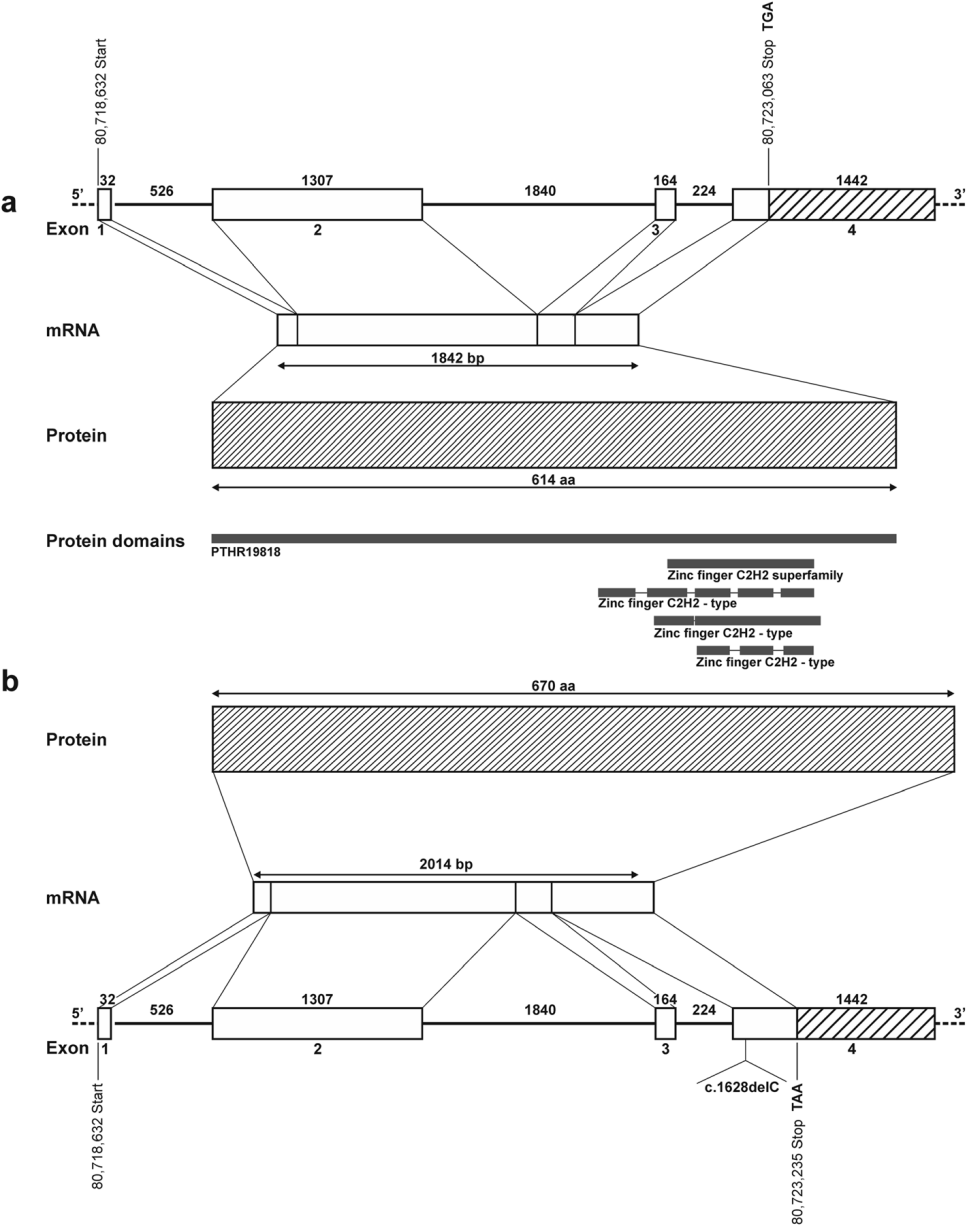
Gene model of the wild type (**a**) and mutant (**b**) *ZIC2* according to UMD3.1. The location of the ZIC2:g.80722845 TC>T (ARS-UCD1.2: g.76742066TC>T) variant in exon 4 is marked. The wild type and mutant mRNA in base pairs (bp) and amino acid (aa) sequence and protein domains are given

We screened 755 filtered genetic and structural variants (Additional file 1: Table S1) which were filtered out under the hypothesis of a recessive inheritance (heterozygous in both parents and homozygous mutant in the case) or a de novo dominant germline mutation (heterozygous in the case and homozygous wild type in both parents and half-sibs) (Fig. 3). There were no variants, which fulfilled the condition of a recessive inheritance. Then we searched for candidate genes (Additional file 2: Table S2) among the filtered variants. The sole candidate gene with a variant identified in the case was *ZIC2*. A 1-bp deletion (g.80722845TC>T; ARS-UCD1.2:g.76742066TC>T) in exon 4 of the candidate gene *ZIC2* (Fig. 4) was heterozygous in the affected calf and homozygous wild type in both parents and all other Limousin herdmates and private controls. This variant was not yet deposited in dbSNP. Due to this heterozygous 1-bp deletion, a new stop codon (TAA) was predicted for position ZIC2:g.80723235 (ARS-UCD1.2:g.76742456). The wild type stop codon (TGA) at position ZIC2:g.80723063 (ARS-UCD1.2:g.76742284) was removed.

The protein effect of this variant was predicted to be probably damaging (0.991) using PolyPhen-2. The sequence of the mutant protein has a likely amino acid (aa) exchange of prolin to arginine at position 543 (p.Pro543fs; ARS-UCD1.2:p.Pro454fs) followed by an altered sequence of 127 aa (Additional file 3: Figure S1). The mutant ZIC2 protein was predicted to have additional 56 aa in comparison to the wild type protein and instead of 614 aa (ARS-UCD1.2:525aa) an increase to 670 aa (ARS-UCD1.2:581aa). The frameshift mutation was predicted to cause a truncation of the *Zinc Finger Protein Zic and Gli* (PTHR19818) domain at codon 543 using ORFfinder.

## Discussion

Congenital FND, arhinencephaly and severe defects of the central nervous system were the main and characteristic findings in the present case. Similar brain malformations like HPE and aprosencephaly were previously reported in a Hereford calf [9] and a lamb [21]. Contrasting to the present case, bone structures of the head were completely absent in this lamb. A Brown Swiss crossbreed calf showed very similar malformations of facial structures with the present case [13]. Muzzle and nasal bones were completely absent and the mandibles were strongly curved upwards as well as a membraned opening to the intracranial cavity were common findings among our case and this Brown Swiss crossbreed FND calf. Ophthalmological malformations characterized by hypotelorism without indications of cyclopia or synophthalmia in the present case were also often seen in previously reported FND cases in cattle [1, 12–14, 16]. A cleft palate was an accompanying sign in a Japanese Brown calf [11], Murrah Buffalo calf [19] as well in our case. We observed a shortened tail in the affected Limousin calf investigated here, but could not confirm a complete absence of the tail as previously described in a crossbreed calf with mild FND [15].

In human with arhinencephaly, synophthalmia and HPE also heart defects like inter-ventricular communication orifice were found similar to the complex heart malformations seen in the present case of our Limousin calf [3].

The WGS data analysis of the present case retrieved a heterozygous frameshift variant in *ZIC2*, which is a strong candidate gene for HPE in human and mouse. We validated the wild type genotypes in the parents, two half-siblings and controls of the affected Limousin calf but were not able to get Sanger sequences from a further tissue sample of the affected calf. The reason Sanger sequencing failed is that DNA degraded because sampling could only be done from the insufficiently cooled body about 2 weeks after birth of the affected calf. In cases of human HPE associated with *ZIC2* mutations, only mild to moderate facial malformations were observed [25–27]. Despite the mild facial phenotypes, these patients often exhibit severe HPE and neurological impairments [25–27]. However, mouse models revealed severe facial malformations in embryos like as hypotelorism, synophthalmy and cyclopia [28]. In human, patients with a 13q deletion syndrome including *ZIC2*, showed diverse phenotypes with congenital defects including craniofacial dysmorphy, HPE, aprosencephaly and heart malformations [29]. The subfamily PTHR19818:SF18 is part of the ‘Zinc Finger Protein ZIC and GLI’ protein domain family. Due to RNA polymerase II regulatory region sequence and transcription factor activity, the molecular function of this protein domain consists in creating the zinc finger transcription factor that regulates mRNA transcription [30]. Combined regulative function of *GLI* and *ZIC* genes responds to inductive signals and induces patterned neural cell differentiation [31]. The zinc-finger domains of the Zic and Gli factors bind to the identical target sequences as the transcription factors, which mediate hedgehog signals [32]. We suggest that the modified *ZIC2* protein domain in this case altered mRNA transcription regulation and the neural cell differentiation in early embryonic development.

We excluded an autosomal recessive mode of inheritance for the present FND phenotype. Only the affected calf had a heterozygous mutant genotype for the *ZIC2* candidate variant and all family members including parents were homozygous wild type. A paternal or maternal germline mutation may be likely the source of the *ZIC2* frameshift or even a novel mutation in very early embryonic development may lead to a post-zygotic heterozygous mutant genotype [33]. For the present case, the hypothesis of a paternal germline mutation seems less likely based on the pattern of the *ZIC2* frameshift mutation in the Limousin family and the frequency of FND in about 120 progeny. In human, pathogenic *ZIC2* variants are largely de novo and in addition, more frequently de novo than any other HPE-associated genes [34]. In addition, *ZIC2* is one of the most commonly heterozygous mutated genes in human HPE patients [35, 36]. A large spectrum of mutations were reported in human and most of them were located in zinc finger domains [34–36]. Mutations within the *N*-terminal region resulted more often in the most severe HPE cases (alobar form), in which the brain is not divided into hemispheres. Semilobar forms, characterized by incomplete forebrain division, were more frequently associated with mutations within the *C*-terminal region [34].

## Conclusions

In the present study, we identified a de novo frameshift variant in *ZIC2* for lethal frontonasal dysplasia in a Limousin calf. To the authors’ best knowledge, this is the first report of an FND associated mutation in cattle identified through WGS.

## Methods

### Animals

The examined male purebred Limousin calf was stillborn in July 2017 after an extended duration of pregnancy by 22 days. We got this stillborn calf from the private owner and herdbook breeder of this Limousin herd. According to the report of the owner, the calf was the first case of any skeletal malformations seen in this cattle herd. The sire of the affected calf was a natural service Limousin bull, registered in the Limousin herd book. This bull sired all heifers and cows of this herd in the calving period when the case was observed. The dam and the sire of the calf were in healthy condition and had normal appearance. Further, all half-siblings of the affected calf were in healthy condition and had no congenital abnormalities. We collected tissue samples from the affected calf and EDTA-blood samples from the *vena jugularis* of the sire and dam of the affected calf, and further of two maternal half-siblings, 9 paternal half-siblings and three unrelated control animals. Pedigree data from the herd with the affected calf were collected and edited for analysis.

### Phenotype

The stillborn affected calf was submitted to CT and a patho-morphological examination. CT scans were acquired with a multislice helical CT scanner (Brilliance TM CT 16 BigBore, Philips Healthcare, The Netherlands) using conventional settings (120 kV and 315 mA) and 0.8 mm slice thickness. Necropsy and histopathological examinations were performed for the affected calf with particular reference on the malformations of the head and brain. Samples of the membrane covering the defect on the front skull were taken, fixed in 10% formalin and embedded in paraffin. Sections of 4 μm were stained with hematoxylin and eosin and were microscopically examined.

### Whole genome sequencing

To identify potential causal variants, genomic DNA from the affected calf, its dam and sire, one female maternal and one male paternal unaffected half-siblings were isolated using a chloroform extraction. Quality control of the isolated DNA was performed and ensuing libraries from these samples were prepared according to the manufacturers protocols using the NEBNext Ultra DNA Library Prep Kit for Illumina (New England BioLabs, Ipswich, MA, USA).WGS was performed on an Illumina NextSeq500 (Illumina, San Diego, CA, USA) in a 2 × 150 bp paired-end mode. Quality control of the WGS data was done using fastqc 0.11.5 [37] and the reads were trimmed using PRINSEQ (V 0.20.4) [38]. The remaining data were mapped to the bovine reference genome UMD 3.1. (Ensembl) using BWA 0.7.13 [39]. Mean coverage was 8.38X with a mean error rate (mismatches/bases mapped) of 1.84 × 10^− 2^ and a mean mapping rate of 96.62%. Bam-files were sorted, indexed and marked for duplicates using SAMtools 1.3.1 [40] and Picard tools (http://broadinstitute.github.io/picard/, version 2.9.4). Variants were called with GATK, version 4.0 [41], using Base Quality Score Recalibration (BQSR), Haplotype Caller and Variant Recalibrator. Variant calling was done for the five Limousin samples and further 89 WGS data from cattle of the breeds Holstein, Fleckvieh, Braunvieh, Vorderwald, German Angus, Galloway, Limousin, Charolais, Hereford, Tyrolean Grey and Miniature Zebu. The resulting vcf-file was used for screening private variants in the FND case. Variants selected for analysis had a read depth of 2–999 and quality score values > 20. We first screened the bovine *ALX1* gene (ENSBTAG00000014977, UMD3.1) on BTA 514991697–15,013,533 bp (ARS-UCD1.2: 14917352–14,939,191 bp) from the vcf-file using SAS, version 9.4 (Statistical Analysis System, Cary, NC, USA) to identify all coding and noncoding variants of the affected calf. All further variants filtered had high or moderate effects according to prediction toolbox SNPEff version 4.3 q (2017-08-30, SNPEff database UMD3.1.86) [42]. We filtered for variants, which were homozygous mutant in the affected Limousin calf and heterozygous mutant in the dam and sire and heterozygous or homozygous wild type in both half-siblings using SAS, version 9.4. In addition, we screened for variants, which were heterozygous mutant in the affected Limousin calf but homozygous wild in both parents and both half-siblings. All filtered variants had to be homozygous wild type in all 89 private controls and not yet deposited in dbSNP. Filtered variants were screened for candidate genes associated with the terms FND, arhinencephaly, holosprosencephaly and cyclopia for all mammalian species retrieved from National Center for Biotechnology Information (NCBI, www.ncbi.nlm.nih.gov) and Online Mendelian Inheritance in Animals (OMIA, www.omia.org, date of access: 12 April 2020). In addition, we mapped the NGS data to ARS-UCD1.2 and visualized the bam-files at critical positions for possible causative variants using Integrated Genomics Viewer (IGV) [43]. In order to check for large deletions/insertions and duplications as well as chromosomal aberrations, we used the breakpoint prediction framework LUMPY [44]. We compared all WGS data from 89 private controls with data from the affected calf and its relatives. Structural variants were filtered out which were heterozygous mutant in the affected calf and homozygous wild type in all other animals or which were homozygous mutant in the affected calf and heterozygous in parents and not homozygous mutant in its half-sibs. These structural variants were investigated for their potential functional effects by comparison of their genomic positions with those regions harboring candidate genes for HPE in human and animals according to National Center for Biotechnology Information (NCBI, www.ncbi.nlm.nih.gov).

To verify the previously investigated protein effect of the *ZIC2* variant, we applied the *PolyPhen-2* (Polymorphism Phenotyping v2) tool. To visualize predicted changes in the *ZIC2* protein, we used ORFfinder (NCBI, www.ncbi.nlm.nih.gov/orffinder). The resulting amino acid (aa) sequences of the wild type protein (NSBTAP00000035289, UMD3.1) and predicted mutant protein were compared and aligned using ClustalW2, version 2.1 [45]. Predicted protein domains were identified using InterPro [46].

## Supplementary Information

**Additional file 1: Table S1.** Filtering result of whole genome sequencing data revealed 755 variants. Only one variant (printed in bold) on BTA 12 within the critical candidate gene *ZIC2*, which was associated with holoprosencephaly in mammalian animals, exclusively occurred in the affected calf. The dam, the sire, one male paternal (a) and one female maternal half-sibling (b) were homozygous wild type, as well as further 89 controls of the breeds Holstein, Fleckvieh, Braunvieh, Vorderwald, German Angus, Galloway, Limousin, Charolais, Hereford, Tyrolean Grey and Miniature Zebu.

**Additional file 2: Table S2.** Candidate genes for frontonasal dysplasia (FND), arhinencephaly, holoprosencephaly and cyclopia in mammalian animals according to NCBI. The bovine orthologues gene were presented with the chromosomal position according to UMD3.1. The candidate gene *ZIC2* which was filtered out of whole genome sequencing data is in bold.

**Additional file 3: Figure S1.** Protein sequences of the wild type and mutant ZIC2 protein (NSBTAP00000035289) are compared. The amino acid frameshift of prolin to arginine at position 543 is red framed. The extension of the protein sequence over 127 amino acids for the mutant protein is given. Asterisks mark identical amino acids between the wild type and mutant sequences.

## Data Availability

Variants were submitted to European Variation Archive (https://www.ebi.ac.uk/eva/?Home) referred to as PRJEB36774 (ARS-UCD1.2:g.76742066TC>T). WGS data of the calves, the sire, the dam and the maternal and paternal half-siblings were deposited in NCBI Sequence Read Archive under the project number PRJNA526664 (SAMN11107014, SAMN11107015, SAMN11107016, SAMN11107017, SAMN11107018). Further WGS data were retrieved from Sequence Read Archive (SRA, NCBI).

## Abbreviations

aa: Amino acid
*ALX*: *Aristaless-like homeoboxprotein*
BQSR: Base quality score recalibration
BTA: *Bos taurus* autosome
CT: Computed tomography
EDTA: Ethylenediamine tetraacetic acid
FND: Frontonasal dysplasia
*Gli*: *Glioma-associated oncogene*
HPE: Holoprosencephaly
IGV: Integrated Genomics Viewer
OMIA: Online Mendelian Inheritance in Animals
PCR: Polymerase chain reaction
SAS: Statistical Analysis System
WGS: Whole genome sequencing
*ZIC2*: *Zinc finger family member 2*
