# Supplementary material for: De novo ZIC2 frameshift variant associated with frontonasal dysplasia in a Limousin calf
Source: BMC Genomics. 2021 Jan 2;22:1. doi: 10.1186/s12864-020-07350-y (PMC7777292; doi:10.1186/s12864-020-07350-y)
Supplement: Supplementary file 1 — Additional file 1: Table S1. Filtering result of whole genome sequencing data revealed 755 variants. Only one variant (printed in bold) on BTA 12 within the critical candidate gene ZIC2, which was associated with holoprosencephaly in mammalian animals, exclusively occurred in the affected calf. The dam, the sire, one male paternal (a) and one female maternal half-sibling (b) were homozygous wild type, as well as further 89 controls of the breeds Holstein, Fleckvieh, Braunvieh, Vorderwald, German Angus, Galloway, Limousin, Charolais, Hereford, Tyrolean Grey and Miniature Zebu. [file 12864_2020_7350_MOESM1_ESM.docx]

**Additional file 1: Table S1.** Filtering result of whole genome sequencing data revealed 755 variants. Only one variant (printed in bold) on BTA 12 within the critical candidate gene *ZIC2*, which was associated with holoprosencephaly in mammalian animals, exclusively occurred in the affected calf. The dam, the sire, one male paternal (a) and one female maternal half-sibling (b) were homozygous wild type, as well as further 89 controls of the breeds Holstein, Fleckvieh, Braunvieh, Vorderwald, German Angus, Galloway, Limousin, Charolais, Hereford, Tyrolean Grey and Miniature Zebu.

| **Gene** | **BTA** | **Position** | **Base change** | **Genotype** | | | | | **Transcript** | **cDNA** | **Protein** |
| --- | --- | --- | --- | --- | --- | --- | --- | --- | --- | --- | --- |
|  |  |  |  | **Case** | **Sire** | **Dam** | **a** | **b** |  |  |  |
| MCM9 | 9 | 32614164 | C>CGTACCGCCCTGA | 0/1 | 0/0 | 0/0 | 0/0 | 0/0 | ENSBTAT00000002665.5 | c.2405_2406insGTACCGCCCTGA | p.Ala802_Gly803insTyrArgProGlu |
| MCM9 | 9 | 32614166 | G>GTTGTTCCCAATGCACCAGATGCGCGATCACTTCGGTATCCGTTTCAGAATCAAAACGGTAACCGCGCTCGATCAGCAATTCACGCAAAGGTTCGTGGTTTTCGAT | 0/1 | 0/0 | 0/0 | 0/0 | 0/0 | ENSBTAT00000002665.5 | c.2407_2408insTTGTTCCCAATGCACCAGATGCGCGATCACTTCGGTATCCGTTTCAGAATCAAAACGGTAACCGCGCTCGATCAGCAATTCACGCAAAGGTTCGTGGTTTTCGAT | p.Gly803delinsValValProAsnAlaProAspAlaArgSerLeuArgTyrProPheGlnAsnGlnAsnGlyAsnArgAlaArgSerAlaIleHisAlaLysValArgGlyPheArgTer |
| AKAP1 | 19 | 8181385 | G>GCATTGAAGGTATTGAGAATCTCAGAGCCACTCATGCTCGGGTGTCGGTACCGATCATCGGCATTATCAAACGCGACCTGCCAGATTCGCCGGTGCGTATCACGCCTTTTCT | 0/1 | 0/0 | 0/0 | 0/0 | 0/0 | ENSBTAT00000049153.3 | c.143_144insCATTGAAGGTATTGAGAATCTCAGAGCCACTCATGCTCGGGTGTCGGTACCGATCATCGGCATTATCAAACGCGACCTGCCAGATTCGCCGGTGCGTATCACGCCTTTTCT | p.Arg48_Ala49insIleGluGlyIleGluAsnLeuArgAlaThrHisAlaArgValSerValProIleIleGlyIleIleLysArgAspLeuProAspSerProValArgIleThrProPheLeu |
| PARP14 | 1 | 67651832 | A>ACCGAT | 0/1 | 0/0 | 0/0 | 0/0 | 0/0 | ENSBTAT00000022152.5 | c.4875_4876insCGATC | p.Thr1627fs |
| PARP14 | 1 | 67651835 | G>GCGCTCGCAGGATTACAGCGCCATCAAGGACGTGTTCCGTCCGGGACATGCGGATTACACCTACGAACAAAAATACGGCGTGCGCGATTATCGTGGCGGTGGCCGTTCTTCTGCCCGCGA | 0/1 | 0/0 | 0/0 | 0/0 | 0/0 | ENSBTAT00000022152.5 | c.4877_4878insCGCTCGCAGGATTACAGCGCCATCAAGGACGTGTTCCGTCCGGGACATGCGGATTACACCTACGAACAAAAATACGGCGTGCGCGATTATCGTGGCGGTGGCCGTTCTTCTGCCCGCGA | p.Arg1626fs |
| PDIA5 | 1 | 68012172 | CAGGGA>C | 0/1 | 0/0 | 0/0 | 0/0 | 0/0 | ENSBTAT00000025128.3 | c.-4_1delAGGGA | p.Met1fs |
| PDIA5 | 1 | 68012178 | T>TTCC | 0/1 | 0/0 | 0/0 | 0/0 | 0/0 | ENSBTAT00000025128.3 | c.2_3insTCC | p.Met1delinsIlePro |
| **Gene** | **BTA** | **Position** | **Base change** | **Genotype** | | | | | **Transcript** | **cDNA** | **Protein** |
|  |  |  |  | **Case** | **Sire** | **Dam** | **a** | **b** |  |  |  |
| PDIA5 | 1 | 68012180 | G>C | 0/1 | 0/0 | 0/0 | 0/0 | 0/0 | ENSBTAT00000025128.3 | c.4G>C | p.Ala2Pro |
| PDIA5 | 1 | 68012182 | G>GTATTCTTTGTGCTACT | 0/1 | 0/0 | 0/0 | 0/0 | 0/0 | ENSBTAT00000025128.3 | c.6_7insTATTCTTTGTGCTACT | p.Arg3fs |
| PDIA5 | 1 | 68012184 | G>GTTAT | 0/1 | 0/0 | 0/0 | 0/0 | 0/0 | ENSBTAT00000025128.3 | c.8_9insTTAT | p.Val4fs |
| PDIA5 | 1 | 68012186 | G>C | 0/1 | 0/0 | 0/0 | 0/0 | 0/0 | ENSBTAT00000025128.3 | c.10G>C | p.Val4Leu |
| PDIA5 | 1 | 68012189 | G>C | 0/1 | 0/0 | 0/0 | 0/0 | 0/0 | ENSBTAT00000025128.3 | c.13G>C | p.Val5Leu |
| PDIA5 | 1 | 68012193 | C>CATCT | 0/1 | 0/0 | 0/0 | 0/0 | 0/0 | ENSBTAT00000025128.3 | c.17_18insATCT | p.Ala7fs |
| PDIA5 | 1 | 68012197 | G>GACCA | 0/1 | 0/0 | 0/0 | 0/0 | 0/0 | ENSBTAT00000025128.3 | c.21_22insACCA | p.Trp8fs |
| PDIA5 | 1 | 68012199 | G>GC | 0/1 | 0/0 | 0/0 | 0/0 | 0/0 | ENSBTAT00000025128.3 | c.23_24insC | p.Trp8fs |
| PDIA5 | 1 | 68012202 | T>TCCCCGCCGCA | 0/1 | 0/0 | 0/0 | 0/0 | 0/0 | ENSBTAT00000025128.3 | c.26_27insCCCCGCCGCA | p.Leu10fs |
| PDIA5 | 1 | 68012205 | T>A | 0/1 | 0/0 | 0/0 | 0/0 | 0/0 | ENSBTAT00000025128.3 | c.29T>A | p.Leu10His |
| PDIA5 | 1 | 68012207 | C>CGG | 0/1 | 0/0 | 0/0 | 0/0 | 0/0 | ENSBTAT00000025128.3 | c.31_32insGG | p.Leu11fs |
| PDIA5 | 1 | 68012208 | T>TAAAAACAGAGCATCA | 0/1 | 0/0 | 0/0 | 0/0 | 0/0 | ENSBTAT00000025128.3 | c.32_33insAAAAACAGAGCATCA | p.Leu11_Pro12insLysThrGluHisGln |
| PDIA5 | 1 | 68012211 | C>CTATGTCCTGGTG | 0/1 | 0/0 | 0/0 | 0/0 | 0/0 | ENSBTAT00000025128.3 | c.35_36insTATGTCCTGGTG | p.Pro12_Leu13insMetSerTrpTrp |
| RNF168 | 1 | 71631668 | C>CCAGTTATGCCGTTTTGACCGAAGATGAACGCGAATTAGGTGTCTGTGTCGTCGATATTGGTGGCGGAACCATGGATATCGCTGTTTATACCGGTGGCGCACTGCGTCATACTAAGGT | 0/1 | 0/0 | 0/0 | 0/0 | 0/0 | ENSBTAT00000017712.4 | c.1388_1389insACCTTAGTATGACGCAGTGCGCCACCGGTATAAACAGCGATATCCATGGTTCCGCCACCAATATCGACGACACAGACACCTAATTCGCGTTCATCTTCGGTCAAAACGGCATAACTG | p.Met463delinsIleProTerTyrAspAlaValArgHisArgTyrLysGlnArgTyrProTrpPheArgHisGlnTyrArgArgHisArgHisLeuIleArgValHisLeuArgSerLysArgHisAsnTrp |
| EIF4G1 | 1 | 83475873 | G>GCCTGCGCGGGTACCGGCATCCAGCGCGGTAAGGATAAACAGCGCTTCAAACAGAATACCGAAGTGGTACCAGAAGCCCAT | 0/1 | 0/0 | 0/0 | 0/0 | 0/0 | ENSBTAT00000032213.4 | c.3005_3006insATGGGCTTCTGGTACCACTTCGGTATTCTGTTTGAAGCGCTGTTTATCCTTACCGCGCTGGATGCCGGTACCCGCGCAGG | p.Arg1003fs |
| ENSBTAG00000047570 | 1 | 110024000 | A>T | 0/1 | 0/0 | 0/0 | 0/0 | 0/0 | ENSBTAT00000063226.1 | c.688T>A | p.Ser230Thr |
| RSRC1 | 1 | 110163851 | G>T | 0/1 | 0/0 | 0/0 | 0/0 | 0/0 | ENSBTAT00000044707.2 | c.490C>A | p.Arg164Ser |
| **Gene** | **BTA** | **Position** | **Base change** | **Genotype** | | | | | **Transcript** | **cDNA** | **Protein** |
|  |  |  |  | **Case** | **Sire** | **Dam** | **a** | **b** |  |  |  |
| MED12L | 1 | 117620501 | TG>T | 0/1 | 0/0 | 0/0 | 0/0 | 0/0 | ENSBTAT00000010239.5 | c.2752delC | p.His918fs |
| AGTR1 | 1 | 120403950 | T>G | 0/1 | 0/0 | 0/0 | 0/0 | 0/0 | ENSBTAT00000063575.1 | c.139A>C | p.Ser47Arg |
| TXLNA | 1 | 130659011 | GGGA>G | 0/1 | 0/0 | 0/0 | 0/0 | 0/0 | ENSBTAT00000017100.3 | c.814_816delGAG | p.Glu272del |
| PIK3CB | 1 | 131432991 | AAC>A | 0/1 | 0/0 | 0/0 | 0/0 | 0/0 | ENSBTAT00000009083.5 | c.290_291delCA | p.Thr97fs |
| EPHB1 | 1 | 135192064 | CAG>C | 0/1 | 0/0 | 0/0 | 0/0 | 0/0 | ENSBTAT00000020455.5 | c.2815_2816delCT | p.Leu939fs |
| ACAD11 | 1 | 138084820 | A>G | 0/1 | 0/0 | 0/0 | 0/0 | 0/0 | ENSBTAT00000054042.2 | c.1593-2A>G |  |
| WDR4 | 1 | 144659297 | T>TGCGTTACGACATTGAGGATA | 0/1 | 0/0 | 0/0 | 0/0 | 0/0 | ENSBTAT00000028643.4 | c.823_824insTATCCTCAATGTCGTAACGC | p.Asp275fs |
| WDR4 | 1 | 144659299 | C>CGGCGGT | 0/1 | 0/0 | 0/0 | 0/0 | 0/0 | ENSBTAT00000028643.4 | c.821_822insACCGCC | p.Leu274_Asp275insProPro |
| PCBP3 | 1 | 147333283 | T>TTAA | 0/1 | 0/0 | 0/0 | 0/0 | 0/0 | ENSBTAT00000043482.2 | c.47_48insTAA | p.Ile16_Arg17insAsn |
| PCBP3 | 1 | 147333284 | C>CGGGCTGATCACGCAGGCGTTTTTCCCACAGCACGATATCGGGCAGTTACATCTTCCCCTTTTACAGCGTTTTTCCGCGCAACAACGTCAGTTACAT | 0/1 | 0/0 | 0/0 | 0/0 | 0/0 | ENSBTAT00000043482.2 | c.48_49insGGGCTGATCACGCAGGCGTTTTTCCCACAGCACGATATCGGGCAGTTACATCTTCCCCTTTTACAGCGTTTTTCCGCGCAACAACGTCAGTTACAT | p.Ile16_Arg17insGlyLeuIleThrGlnAlaPhePheProGlnHisAspIleGlyGlnLeuHisLeuProLeuLeuGlnArgPheSerAlaGlnGlnArgGlnLeuHis |
| PCNT | 1 | 147810949 | AT>A | 0/1 | 0/0 | 0/0 | 0/0 | 0/0 | ENSBTAT00000029328.5 | c.5909delT | p.Ile1970fs |
| AMER3 | 2 | 1405097 | AC>A | 0/1 | 0/0 | 0/0 | 0/0 | 0/0 | ENSBTAT00000054423.2 | c.916delC | p.His306fs |
| FSIP2 | 2 | 10625667 | T>A | 0/1 | 0/0 | 0/0 | 0/0 | 0/0 | ENSBTAT00000013129.4 | c.6280A>T | p.Asn2094Tyr |
| FSIP2 | 2 | 10625668 | A>T | 0/1 | 0/0 | 0/0 | 0/0 | 0/0 | ENSBTAT00000013129.4 | c.6279T>A | p.Asn2093Lys |
| ENSBTAG00000026986 | 2 | 18178578 | GT>G | 0/1 | 0/0 | 0/0 | 0/0 | 0/0 | ENSBTAT00000061449.2 | c.31590delT | p.Pro10531fs |
| GPR155 | 2 | 22394116 | CT>C | 0/1 | 0/0 | 0/0 | 0/0 | 0/0 | ENSBTAT00000028831.4 | c.2601delT | p.Lys868fs |
| TBR1 | 2 | 35071029 | A>AGGCGATCCCGATGCTGGTGGTACTGGGCACCATCACCGATGCTCCGGCTACCCTGATCAACGTGATTCATGATTT | 0/1 | 0/0 | 0/0 | 0/0 | 0/0 | ENSBTAT00000028908.4 | c.411_412insAAATCATGAATCACGTTGATCAGGGTAGCCGGAGCATCGGTGATGGTGCCCAGTACCACCAGCATCGGGATCGCC | p.Phe137_Ser138insLysSerTerIleThrLeuIleArgValAlaGlyAlaSerValMetValProSerThrThrSerIleGlyIleAla |
| WDSUB1 | 2 | 37169549 | C>T | 0/1 | 0/0 | 0/0 | 0/0 | 0/0 | ENSBTAT00000064632.1 | c.65C>T | p.Ser22Phe |
| **Gene** | **BTA** | **Position** | **Base change** | **Genotype** | | | | | **Transcript** | **cDNA** | **Protein** |
|  |  |  |  | **Case** | **Sire** | **Dam** | **a** | **b** |  |  |  |
| ACVR1C | 2 | 38573835 | GA>G | 0/1 | 0/0 | 0/0 | 0/0 | 0/0 | ENSBTAT00000025753.5 | c.437delA | p.Lys146fs |
| ACVR1C | 2 | 38573839 | A>T | 0/1 | 0/0 | 0/0 | 0/0 | 0/0 | ENSBTAT00000025753.5 | c.436A>T | p.Lys146* |
| CXCR4 | 2 | 61585015 | G>GTGGCGTTCCAGCAACGGTTTCAGGAAGCGTGCGGTATGCGATTCTTTGCATTCCGCCACGGTTTCCGGCGTA | 0/1 | 0/0 | 0/0 | 0/0 | 0/0 | ENSBTAT00000001406.3 | c.762_763insTGGCGTTCCAGCAACGGTTTCAGGAAGCGTGCGGTATGCGATTCTTTGCATTCCGCCACGGTTTCCGGCGTA | p.Leu254_Pro255insTrpArgSerSerAsnGlyPheArgLysArgAlaValCysAspSerLeuHisSerAlaThrValSerGlyVal |
| LCT | 2 | 61901131 | G>GACGAGCCGGACGCGTTCTGGGACGCCTTGCA | 0/1 | 0/0 | 0/0 | 0/0 | 0/0 | ENSBTAT00000020185.4 | c.4450_4451insACGAGCCGGACGCGTTCTGGGACGCCTTGCA | p.Ala1484fs |
| LCT | 2 | 61901134 | A>ACTTG | 0/1 | 0/0 | 0/0 | 0/0 | 0/0 | ENSBTAT00000020185.4 | c.4453_4454insCTTG | p.Asn1485fs |
| LCT | 2 | 61901135 | A>ATCCTGCCGGC | 0/1 | 0/0 | 0/0 | 0/0 | 0/0 | ENSBTAT00000020185.4 | c.4454_4455insTCCTGCCGGC | p.Ile1486fs |
| C1QL2 | 2 | 71284972 | C>CGCCATGTGGCTGTAAGTAGCCAAGAACCCAGTGCACTCTACAGCAGGAAAATCCTG | 0/1 | 0/0 | 0/0 | 0/0 | 0/0 | ENSBTAT00000050672.3 | c.378_379insCAGGATTTTCCTGCTGTAGAGTGCACTGGGTTCTTGGCTACTTACAGCCACATGGC | p.Gly127fs |
| CLASP1 | 2 | 73588280 | A>T | 0/1 | 0/0 | 0/0 | 0/0 | 0/0 | ENSBTAT00000065657.1 | c.1688T>A | p.Leu563His |
| TYW5 | 2 | 88930130 | T>A | 0/1 | 0/0 | 0/0 | 0/0 | 0/0 | ENSBTAT00000025608.4 | c.84A>T | p.Lys28Asn |
| CPS1 | 2 | 98812301 | G>GCTCAAGGCCACCTCGGGTGGTGGCGGTCGCGGC | 0/1 | 0/0 | 0/0 | 0/0 | 0/0 | ENSBTAT00000022158.5 | c.1755_1756insCTCAAGGCCACCTCGGGTGGTGGCGGTCGCGGC | p.Met585_Ile586insLeuLysAlaThrSerGlyGlyGlyGlyArgGly |
| CPS1 | 2 | 98812303 | T>TTCGTCGCTGCAACAG | 0/1 | 0/0 | 0/0 | 0/0 | 0/0 | ENSBTAT00000022158.5 | c.1757_1758insTCGTCGCTGCAACAG | p.Ile586_Arg587insArgArgCysAsnSer |
| CPS1 | 2 | 98812307 | T>TGAAGAAC | 0/1 | 0/0 | 0/0 | 0/0 | 0/0 | ENSBTAT00000022158.5 | c.1761_1762insGAAGAAC | p.Ser588fs |
| CPS1 | 2 | 98812309 | C>CGAGCAAGCCTTCCCGCGGG | 0/1 | 0/0 | 0/0 | 0/0 | 0/0 | ENSBTAT00000022158.5 | c.1763_1764insGAGCAAGCCTTCCCGCGGG | p.Ala589fs |
| CPS1 | 2 | 98812311 | G>GATCTCGGAAGCGACCAAGGC | 0/1 | 0/0 | 0/0 | 0/0 | 0/0 | ENSBTAT00000022158.5 | c.1765_1766insATCTCGGAAGCGACCAAGGC | p.Ala589fs |
| CPS1 | 2 | 98812314 | T>TTGGTTCGGCCG | 0/1 | 0/0 | 0/0 | 0/0 | 0/0 | ENSBTAT00000022158.5 | c.1768_1769insTGGTTCGGCCG | p.Tyr590fs |
| **Gene** | **BTA** | **Position** | **Base change** | **Genotype** | | | | | **Transcript** | **cDNA** | **Protein** |
|  |  |  |  | **Case** | **Sire** | **Dam** | **a** | **b** |  |  |  |
| ERBB4 | 2 | 99664378 | C>CGCTACATCCATGGAAGCCGCCCGTGCCGCCGTGGCGCTGGCGAAAGTGGAGTATCAG | 0/1 | 0/0 | 0/0 | 0/0 | 0/0 | ENSBTAT00000061483.2 | c.2651_2652insCTGATACTCCACTTTCGCCAGCGCCACGGCGGCACGGGCGGCTTCCATGGATGTAGC | p.Arg884delinsSerTerTyrSerThrPheAlaSerAlaThrAlaAlaArgAlaAlaSerMetAspValAla |
| BCS1L | 2 | 107345716 | T>TCAGCAAGCCGCTGCCGCTGGCCGAACTGGTGGAGTATTTGACGTTTGGG | 0/1 | 0/0 | 0/0 | 0/0 | 0/0 | ENSBTAT00000004967.1 | c.1202_1203insCAGCAAGCCGCTGCCGCTGGCCGAACTGGTGGAGTATTTGACGTTTGGG | p.Met401fs |
| DNPEP | 2 | 108037039 | GC>G | 0/1 | 0/0 | 0/0 | 0/0 | 0/0 | ENSBTAT00000028671.4 | c.1080delG | p.Arg360fs |
| ZBTB8OS | 2 | 121818198 | GA>G | 0/1 | 0/0 | 0/0 | 0/0 | 0/0 | ENSBTAT00000038929.3 | c.12delA | p.Glu6fs |
| ZBTB8OS | 2 | 121818201 | G>T | 0/1 | 0/0 | 0/0 | 0/0 | 0/0 | ENSBTAT00000038929.3 | c.13G>T | p.Gly5Trp |
| ZBTB8OS | 2 | 121818217 | AT>A | 0/1 | 0/0 | 0/0 | 0/0 | 0/0 | ENSBTAT00000038929.3 | c.31delT | p.Tyr11fs |
| LCK | 2 | 122100555 | TC>T | 0/1 | 0/0 | 0/0 | 0/0 | 0/0 | ENSBTAT00000038070.3 | c.274delG | p.Glu92fs |
| TCEB3 | 2 | 129744391 | T>TCA | 0/1 | 0/0 | 0/0 | 0/0 | 0/0 | ENSBTAT00000063789.1 | c.754_755insTG | p.Gln252fs |
| TCEB3 | 2 | 129744392 | G>GCCCGGTGT | 0/1 | 0/0 | 0/0 | 0/0 | 0/0 | ENSBTAT00000063789.1 | c.753_754insACACCGGG | p.Gln252fs |
| TCEB3 | 2 | 129744394 | C>CAACATACGGCGGGTCGTATTATTGGCGCCTTCCGCATAGTGGTTATCCCAGTCAAACAATGCCGGAATAT | 0/1 | 0/0 | 0/0 | 0/0 | 0/0 | ENSBTAT00000063789.1 | c.751_752insATATTCCGGCATTGTTTGACTGGGATAACCACTATGCGGAAGGCGCCAATAATACGACCCGCCGTATGTT | p.Ser251fs |
| DCAF6 | 3 | 681116 | TC>T | 0/1 | 0/0 | 0/0 | 0/0 | 0/0 | ENSBTAT00000027930.5 | c.716delG | p.Gly239fs |
| RXRG | 3 | 3617471 | AC>A | 0/1 | 0/0 | 0/0 | 0/0 | 0/0 | ENSBTAT00000022350.3 | c.511delC | p.Leu171fs |
| RXRG | 3 | 3617474 | T>A | 0/1 | 0/0 | 0/0 | 0/0 | 0/0 | ENSBTAT00000022350.3 | c.512T>A | p.Leu171His |
| CCT3 | 3 | 14551850 | C>CGTTTACGGCACCATCACCTCGCTGACCATTTCCAACGGGCTGAACGAACCGGACCGCGAGGCCGCCGAACGCTGGGTGAGTTTCCTTGAAAAAGCCGATCACGCGACGGCGTGGGT | 0/1 | 0/0 | 0/0 | 0/0 | 0/0 | ENSBTAT00000008358.5 | c.1284_1285insGTTTACGGCACCATCACCTCGCTGACCATTTCCAACGGGCTGAACGAACCGGACCGCGAGGCCGCCGAACGCTGGGTGAGTTTCCTTGAAAAAGCCGATCACGCGACGGCGTGGGT | p.Met429fs |
| **Gene** | **BTA** | **Position** | **Base change** | **Genotype** | | | | | **Transcript** | **cDNA** | **Protein** |
|  |  |  |  | **Case** | **Sire** | **Dam** | **a** | **b** |  |  |  |
| ASH1L | 3 | 15307983 | TA>T | 0/1 | 0/0 | 0/0 | 0/0 | 0/0 | ENSBTAT00000005172.5 | c.6118delA | p.Arg2040fs |
| SLC39A1 | 3 | 16533401 | TC>T | 0/1 | 0/0 | 0/0 | 0/0 | 0/0 | ENSBTAT00000034015.4 | c.558delC | p.Leu187fs |
| SLC39A1 | 3 | 16533404 | T>A | 0/1 | 0/0 | 0/0 | 0/0 | 0/0 | ENSBTAT00000034015.4 | c.559T>A | p.Leu187Met |
| ENSBTAG00000024437 | 3 | 17140260 | C>A | 0/1 | 0/0 | 0/0 | 0/0 | 0/0 | ENSBTAT00000033888.3 | c.186C>A | p.Phe62Leu |
| CGN | 3 | 19297059 | C>G | 0/1 | 0/0 | 0/0 | 0/0 | 0/0 | ENSBTAT00000023352.5 | c.2583G>C | p.Gln861His |
| MTMR11 | 3 | 20721739 | C>T | 0/1 | 0/0 | 0/0 | 0/0 | 0/0 | ENSBTAT00000016579.5 | c.628C>T | p.Arg210* |
| GPR89 | 3 | 21806903 | TC>T | 0/1 | 0/0 | 0/0 | 0/0 | 0/0 | ENSBTAT00000045701.3 | c.970delG | p.Glu324fs |
| ARHGAP29 | 3 | 49473263 | TGA>T | 0/1 | 0/0 | 0/0 | 0/0 | 0/0 | ENSBTAT00000005488.5 | c.3043_3044delAG | p.Arg1015fs |
| MCOLN3 | 3 | 59272795 | CT>C | 0/1 | 0/0 | 0/0 | 0/0 | 0/0 | ENSBTAT00000022589.5 | c.968delT | p.Leu323fs |
| LPAR3 | 3 | 59458674 | A>C | 0/1 | 0/0 | 0/0 | 0/0 | 0/0 | ENSBTAT00000004931.5 | c.895A>C | p.Asn299His |
| LPAR3 | 3 | 59458676 | C>CGTGCTGATAGTGGAATCTTTGTACGATGCGCAATTTGTCGCCAGCCATACGG | 0/1 | 0/0 | 0/0 | 0/0 | 0/0 | ENSBTAT00000004931.5 | c.897_898insGTGCTGATAGTGGAATCTTTGTACGATGCGCAATTTGTCGCCAGCCATACGG | p.Ser300fs |
| SRSF11 | 3 | 75263035 | A>C | 0/1 | 0/0 | 0/0 | 0/0 | 0/0 | ENSBTAT00000020564.5 | c.599T>G | p.Val200Gly |
| DHCR24 | 3 | 92011069 | G>GACA | 0/1 | 0/0 | 0/0 | 0/0 | 0/0 | ENSBTAT00000006153.4 | c.1136_1137insCAA | p.Glu379delinsAspLys |
| DHCR24 | 3 | 92011071 | G>GGTCGGGGCCGATACCGGCGGGTTCACCGGGAGTGATAACAACTCGATTCATACACTTCATCCTT | 0/1 | 0/0 | 0/0 | 0/0 | 0/0 | ENSBTAT00000006153.4 | c.1137_1138insGTCGGGGCCGATACCGGCGGGTTCACCGGGAGTGATAACAACTCGATTCATACACTTCATCCTT | p.Gln380fs |
| MROH7 | 3 | 92152543 | GC>G | 0/1 | 0/0 | 0/0 | 0/0 | 0/0 | ENSBTAT00000061627.2 | c.1133delG | p.Arg378fs |
| MROH7 | 3 | 92152545 | G>A | 0/1 | 0/0 | 0/0 | 0/0 | 0/0 | ENSBTAT00000061627.2 | c.1132C>T | p.Arg378Cys |
| FAAH | 3 | 100326242 | TC>T | 0/1 | 0/0 | 0/0 | 0/0 | 0/0 | ENSBTAT00000009880.4 | c.451delG | p.Asp151fs |
| RAD54L | 3 | 100433735 | CTGA>C | 0/1 | 0/0 | 0/0 | 0/0 | 0/0 | ENSBTAT00000026513.5 | c.471_473delTCA | p.His157del |
| BTBD19 | 3 | 101785060 | GC>G | 0/1 | 0/0 | 0/0 | 0/0 | 0/0 | ENSBTAT00000055916.2 | c.864delG | p.Phe290fs |
| ERI3 | 3 | 102293167 | T>TCCCATGCCGTC | 0/1 | 0/0 | 0/0 | 0/0 | 0/0 | ENSBTAT00000020671.3 | c.189_190insCCCATGCCGTC | p.Gly64fs |
| ERI3 | 3 | 102293171 | A>ACCAG | 0/1 | 0/0 | 0/0 | 0/0 | 0/0 | ENSBTAT00000020671.3 | c.193_194insCCAG | p.Ile65fs |
| **Gene** | **BTA** | **Position** | **Base change** | **Genotype** | | | | | **Transcript** | **cDNA** | **Protein** |
|  |  |  |  | **Case** | **Sire** | **Dam** | **a** | **b** |  |  |  |
| ERI3 | 3 | 102293172 | T>TTTCAGCTTGAGGCCTAAACTGCCGGCCAGTAGACGGGCAATATCCGGGTCGCTGCCGATG | 0/1 | 0/0 | 0/0 | 0/0 | 0/0 | ENSBTAT00000020671.3 | c.194_195insTTCAGCTTGAGGCCTAAACTGCCGGCCAGTAGACGGGCAATATCCGGGTCGCTGCCGATG | p.Ile65_Phe66insSerAlaTerGlyLeuAsnCysArgProValAspGlyGlnTyrProGlyArgCysArgCys |
| IPO13 | 3 | 102693151 | GC>G | 0/1 | 0/0 | 0/0 | 0/0 | 0/0 | ENSBTAT00000025142.5 | c.1034delG | p.Gly345fs |
| P3H1 | 3 | 104080432 | CCA>C | 0/1 | 0/0 | 0/0 | 0/0 | 0/0 | ENSBTAT00000023110.5 | c.1389_1390delCA | p.Lys464fs |
| YRDC | 3 | 108698228 | A>ATCAAGAACGCCACCGGCT | 0/1 | 0/0 | 0/0 | 0/0 | 0/0 | ENSBTAT00000008529.2 | c.484_485insTCAAGAACGCCACCGGCT | p.Asn162delinsIleLysAsnAlaThrGlyTyr |
| YRDC | 3 | 108698230 | T>TGACATGCGCCAGCTGTTTATCGGTGCCGAAGGCAC | 0/1 | 0/0 | 0/0 | 0/0 | 0/0 | ENSBTAT00000008529.2 | c.486_487insGACATGCGCCAGCTGTTTATCGGTGCCGAAGGCAC | p.Pro163fs |
| CLSPN | 3 | 110628584 | G>GCGCTGGGCCTTGGTGGTCGCGACTTTCACAATGCGCCAGTTGCGCAGGATGAACTCGTGGATTTCCGCCTTGATCCAGTGC | 0/1 | 0/0 | 0/0 | 0/0 | 0/0 | ENSBTAT00000003659.4 | c.3484_3485insCGCTGGGCCTTGGTGGTCGCGACTTTCACAATGCGCCAGTTGCGCAGGATGAACTCGTGGATTTCCGCCTTGATCCAGTGC | p.Asp1162delinsAlaLeuGlyLeuGlyGlyArgAspPheHisAsnAlaProValAlaGlnAspGluLeuValAspPheArgLeuAspProValHis |
| ENSBTAG00000048275 | 3 | 113943082 | GA>G | 0/1 | 0/0 | 0/0 | 0/0 | 0/0 | ENSBTAT00000064518.1 | c.74delA | p.Lys25fs |
| PRLH | 3 | 117646646 | CG>C | 0/1 | 0/0 | 0/0 | 0/0 | 0/0 | ENSBTAT00000023421.2 | c.267delG | p.Arg90fs |
| ESPNL | 3 | 118095411 | C>CGGTGCGCAGATCCCGGGTACGGGCCTCGACCAGTTGTTCAAGTTCTGCCCGATTGCGCTGGCGCAAACGGGCGAGCCGCCAGCGCTGGAGTAAAAACAGCCCCAGAAATACCAGCGTCAG | 0/1 | 0/0 | 0/0 | 0/0 | 0/0 | ENSBTAT00000063447.1 | c.1509_1510insGGTGCGCAGATCCCGGGTACGGGCCTCGACCAGTTGTTCAAGTTCTGCCCGATTGCGCTGGCGCAAACGGGCGAGCCGCCAGCGCTGGAGTAAAAACAGCCCCAGAAATACCAGCGTCAG | p.Gly503_Pro504insGlyAlaGlnIleProGlyThrGlyLeuAspGlnLeuPheLysPheCysProIleAlaLeuAlaGlnThrGlyGluProProAlaLeuGluTerLysGlnProGlnLysTyrGlnArgGln |
| PASK | 3 | 120841368 | G>GTACGTACGGGTCA | 0/1 | 0/0 | 0/0 | 0/0 | 0/0 | ENSBTAT00000001044.3 | c.765_766insTGACCCGTACGTA | p.Leu256fs |
| PASK | 3 | 120841370 | C>CGAAAAGCTTGGTATTCCTTA | 0/1 | 0/0 | 0/0 | 0/0 | 0/0 | ENSBTAT00000001044.3 | c.763_764insTAAGGAATACCAAGCTTTTC | p.Gly255fs |
| **Gene** | **BTA** | **Position** | **Base change** | **Genotype** | | | | | **Transcript** | **cDNA** | **Protein** |
|  |  |  |  | **Case** | **Sire** | **Dam** | **a** | **b** |  |  |  |
| PASK | 3 | 120841373 | T>TGTTCAAGGCCAGTTTCGACAAGGCCAATCGTTCATCTGTCAGCTCTTA | 0/1 | 0/0 | 0/0 | 0/0 | 0/0 | ENSBTAT00000001044.3 | c.760_761insTAAGAGCTGACAGATGAACGATTGGCCTTGTCGAAACTGGCCTTGAAC | p.Asp254delinsValArgAlaAspArgTerThrIleGlyLeuValGluThrGlyLeuGluHis |
| AHR | 4 | 25785352 | G>GGAATAT | 0/1 | 0/0 | 0/0 | 0/0 | 0/0 | ENSBTAT00000010187.3 | c.14_15insGAATAT | p.Ser5delinsArgAsnIle |
| AHR | 4 | 25785353 | C>CGCCACAAAACCGAATCGGTGATCCTCGCCACCGGCTACA | 0/1 | 0/0 | 0/0 | 0/0 | 0/0 | ENSBTAT00000010187.3 | c.17_18insCACAAAACCGAATCGGTGATCCTCGCCACCGGCTACAGC | p.Ala6_Asn7insThrLysProAsnArgTerSerSerProProAlaThrAla |
| DNAH11 | 4 | 30688483 | T>TCGACGCCC | 0/1 | 0/0 | 0/0 | 0/0 | 0/0 | ENSBTAT00000061103.2 | c.10316_10317insCGACGCCC | p.Val3440fs |
| DNAH11 | 4 | 30688485 | G>GCGTGACGAAGGATTACGTCTCGCCGGGCTGGCTCGCCAACAGCCCTTAACCTTGCTGTATGCCGCCAAAGATACCCGGCAAAACCATGCGCTGGTGC | 0/1 | 0/0 | 0/0 | 0/0 | 0/0 | ENSBTAT00000061103.2 | c.10318_10319insCGTGACGAAGGATTACGTCTCGCCGGGCTGGCTCGCCAACAGCCCTTAACCTTGCTGTATGCCGCCAAAGATACCCGGCAAAACCATGCGCTGGTGC | p.Val3440fs |
| PCLO | 4 | 37636774 | A>ACTCCGGCTCGACCCGACGCCGATCGCGCACCGCACCCACGGCCTGACGCCGGGTAACCTCAACAAATACGATGCACGTATTGCGGCGATTGATTACACCCTCGCCCATGACGAC | 0/1 | 0/0 | 0/0 | 0/0 | 0/0 | ENSBTAT00000033801.4 | c.705_706insCTCCGGCTCGACCCGACGCCGATCGCGCACCGCACCCACGGCCTGACGCCGGGTAACCTCAACAAATACGATGCACGTATTGCGGCGATTGATTACACCCTCGCCCATGACGAC | p.Glu235_Gly236insLeuArgLeuAspProThrProIleAlaHisArgThrHisGlyLeuThrProGlyAsnLeuAsnLysTyrAspAlaArgIleAlaAlaIleAspTyrThrLeuAlaHisAspAsp |
| LAMB4 | 4 | 49392557 | TG>T | 0/1 | 0/0 | 0/0 | 0/0 | 0/0 | ENSBTAT00000047111.2 | c.4450delC | p.His1484fs |
| LAMB4 | 4 | 49392561 | C>A | 0/1 | 0/0 | 0/0 | 0/0 | 0/0 | ENSBTAT00000047111.2 | c.4447G>T | p.Val1483Phe |
| NEUROD6 | 4 | 65491379 | TCA>T | 0/1 | 0/0 | 0/0 | 0/0 | 0/0 | ENSBTAT00000000728.2 | c.606_607delAC | p.Pro203fs |
| CPED1 | 4 | 86368610 | C>A | 0/1 | 0/0 | 0/0 | 0/0 | 0/0 | ENSBTAT00000063644.1 | c.884C>A | p.Pro295Gln |
| GCC1 | 4 | 92617230 | G>GTCAACAACACT | 0/1 | 0/0 | 0/0 | 0/0 | 0/0 | ENSBTAT00000004036.2 | c.2206_2207insAGTGTTGTTGA | p.Thr736fs |
| GCC1 | 4 | 92617232 | G>GA | 0/1 | 0/0 | 0/0 | 0/0 | 0/0 | ENSBTAT00000004036.2 | c.2204_2205insT | p.Thr736fs |
| GCC1 | 4 | 92617234 | C>CATGGGTGG | 0/1 | 0/0 | 0/0 | 0/0 | 0/0 | ENSBTAT00000004036.2 | c.2202_2203insCCACCCAT | p.Asp735fs |
| **Gene** | **BTA** | **Position** | **Base change** | **Genotype** | | | | | **Transcript** | **cDNA** | **Protein** |
|  |  |  |  | **Case** | **Sire** | **Dam** | **a** | **b** |  |  |  |
| GCC1 | 4 | 92617239 | A>AATTCCGCGGCGCGGCAACACGCGGCGGATCAGTTGATCGATCAGCTGGCTGGCGCCGACGTTCTCGCCTTCCTGCC | 0/1 | 0/0 | 0/0 | 0/0 | 0/0 | ENSBTAT00000004036.2 | c.2197_2198insGGCAGGAAGGCGAGAACGTCGGCGCCAGCCAGCTGATCGATCAACTGATCCGCCGCGTGTTGCCGCGCCGCGGAAT | p.Leu733fs |
| CCDC136 | 4 | 93593616 | G>T | 0/1 | 0/0 | 0/0 | 0/0 | 0/0 | ENSBTAT00000014612.5 | c.2603G>T | p.Ser868Ile |
| CCDC136 | 4 | 93593628 | A>AGCTTCTT | 0/1 | 0/0 | 0/0 | 0/0 | 0/0 | ENSBTAT00000014612.5 | c.2615_2616insGCTTCTT | p.His872fs |
| CCDC136 | 4 | 93593630 | A>ATT | 0/1 | 0/0 | 0/0 | 0/0 | 0/0 | ENSBTAT00000014612.5 | c.2617_2618insTT | p.Thr873fs |
| AGBL3 | 4 | 99679070 | AG>A | 0/1 | 0/0 | 0/0 | 0/0 | 0/0 | ENSBTAT00000033530.4 | c.2370delG | p.Ile791fs |
| AGBL3 | 4 | 99679072 | G>T | 0/1 | 0/0 | 0/0 | 0/0 | 0/0 | ENSBTAT00000033530.4 | c.2370G>T | p.Arg790Ser |
| ZC3HAV1 | 4 | 103489383 | C>G | 0/1 | 0/0 | 0/0 | 0/0 | 0/0 | ENSBTAT00000028806.4 | c.1535G>C | p.Ser512Thr |
| UBN2 | 4 | 103688722 | AC>A | 0/1 | 0/0 | 0/0 | 0/0 | 0/0 | ENSBTAT00000005889.4 | c.4022delC | p.Pro1341fs |
| MGAM | 4 | 106194611 | C>T | 0/1 | 0/0 | 0/0 | 0/0 | 0/0 | ENSBTAT00000065670.1 | c.4834C>T | p.Pro1612Ser |
| PDIA4 | 4 | 112991477 | C>CCGCCATCGGTGAGCTCAACGATCACATCCTGAACCGGCTTATCATAATCCTTCGGGTTGATAAAATCGGTCGCCCCCATCTCTTTGGCGAGGG | 0/1 | 0/0 | 0/0 | 0/0 | 0/0 | ENSBTAT00000022782.4 | c.1252_1253insCCCTCGCCAAAGAGATGGGGGCGACCGATTTTATCAACCCGAAGGATTATGATAAGCCGGTTCAGGATGTGATCGTTGAGCTCACCGATGGCG | p.Ser418delinsThrLeuAlaLysGluMetGlyAlaThrAspPheIleAsnProLysAspTyrAspLysProValGlnAspValIleValGluLeuThrAspGlyGly |
| PDIA4 | 4 | 112991480 | T>A | 0/1 | 0/0 | 0/0 | 0/0 | 0/0 | ENSBTAT00000022782.4 | c.1250A>T | p.Tyr417Phe |
| PDIA4 | 4 | 112991482 | G>T | 0/1 | 0/0 | 0/0 | 0/0 | 0/0 | ENSBTAT00000022782.4 | c.1248C>A | p.Tyr416* |
| NOS3 | 4 | 114393710 | C>CGCTTCGGTGATAAGTCTGCCGCTGACGGTGGATTTCGAAACCGGTTATGGCGCTGACCGTTGCGATAATCTGAGCGCCCTTTTGCAGGCAGGCGTGGTGGGAA | 0/1 | 0/0 | 0/0 | 0/0 | 0/0 | ENSBTAT00000023515.4 | c.3396_3397insGCTTCGGTGATAAGTCTGCCGCTGACGGTGGATTTCGAAACCGGTTATGGCGCTGACCGTTGCGATAATCTGAGCGCCCTTTTGCAGGCAGGCGTGGTGGGAA | p.Leu1133fs |
| ABCB8 | 4 | 114412637 | CG>C | 0/1 | 0/0 | 0/0 | 0/0 | 0/0 | ENSBTAT00000064324.1 | c.1175delG | p.Gly392fs |
| **Gene** | **BTA** | **Position** | **Base change** | **Genotype** | | | | | **Transcript** | **cDNA** | **Protein** |
|  |  |  |  | **Case** | **Sire** | **Dam** | **a** | **b** |  |  |  |
| ZDHHC17 | 5 | 6186093 | TA>T | 0/1 | 0/0 | 0/0 | 0/0 | 0/0 | ENSBTAT00000028999.3 | c.119delA | p.Asn40fs |
| KITLG | 5 | 18342975 | C>CATTTACTTGGGCGAAT | 0/1 | 0/0 | 0/0 | 0/0 | 0/0 | ENSBTAT00000023349.5 | c.202_203insATTCGCCCAAGTAAAT | p.Cys68fs |
| KITLG | 5 | 18342979 | G>GC | 0/1 | 0/0 | 0/0 | 0/0 | 0/0 | ENSBTAT00000023349.5 | c.198_199insG | p.His67fs |
| KITLG | 5 | 18342981 | C>CGTGACAAAGCTGCCGTGCATGTAATCATTGTAGGGCTATCCAGCCAATCCAAAATGCATCGTCTCTATCAATTCGTCGAAGGGGAGTGGCATAG | 0/1 | 0/0 | 0/0 | 0/0 | 0/0 | ENSBTAT00000023349.5 | c.196_197insCTATGCCACTCCCCTTCGACGAATTGATAGAGACGATGCATTTTGGATTGGCTGGATAGCCCTACAATGATTACATGCACGGCAGCTTTGTCAC | p.Ser66fs |
| GTSF1 | 5 | 25738429 | CAAGCTGTGATGACAA>C | 0/1 | 0/0 | 0/0 | 0/0 | 0/0 | ENSBTAT00000047442.1 | c.214_228delGATGACAAAAGCTGT | p.Asp72_Cys76del |
| ESPL1 | 5 | 26895888 | C>G | 0/1 | 0/0 | 0/0 | 0/0 | 0/0 | ENSBTAT00000011761.4 | c.5321G>C | p.Arg1774Pro |
| CSAD | 5 | 27010711 | G>T | 0/1 | 0/0 | 0/0 | 0/0 | 0/0 | ENSBTAT00000010303.5 | c.1309G>T | p.Asp437Tyr |
| WNT1 | 5 | 31001305 | C>CAGCACCACAAAGTTAATCAGGCTGGCCGCCGCCGGCAGGCCCACCAGGACAAACAGTTCGACGAACGGGCTCTTGGTCGGCACCACGGAGCTCCACG | 0/1 | 0/0 | 0/0 | 0/0 | 0/0 | ENSBTAT00000020414.2 | c.522_523insCGTGGAGCTCCGTGGTGCCGACCAAGAGCCCGTTCGTCGAACTGTTTGTCCTGGTGGGCCTGCCGGCGGCGGCCAGCCTGATTAACTTTGTGGTGCT | p.Asp175fs |
| IRAK4 | 5 | 36896354 | CT>C | 0/1 | 0/0 | 0/0 | 0/0 | 0/0 | ENSBTAT00000028115.4 | c.457delA | p.Ser153fs |
| MARS | 5 | 56290712 | G>A | 0/1 | 0/0 | 0/0 | 0/0 | 0/0 | ENSBTAT00000024487.3 | c.2377C>T | p.His793Tyr |
| LRP1 | 5 | 56576632 | G>A | 0/1 | 0/0 | 0/0 | 0/0 | 0/0 | ENSBTAT00000017556.5 | c.9083C>T | p.Pro3028Leu |
| LRP1 | 5 | 56576635 | T>A | 0/1 | 0/0 | 0/0 | 0/0 | 0/0 | ENSBTAT00000017556.5 | c.9080A>T | p.Glu3027Val |
| LRP1 | 5 | 56576638 | T>TGGAGCTGAGCCGCGATATCGCGCAGCGCTTCAACGCCCTTTACGGCGAGGTGTTTAAAGTGCCTGAGC | 0/1 | 0/0 | 0/0 | 0/0 | 0/0 | ENSBTAT00000017556.5 | c.9076_9077insGCTCAGGCACTTTAAACACCTCGCCGTAAAGGGCGTTGAAGCGCTGCGCGATATCGCGGCTCAGCTCC | p.Glu3026fs |
| ATP5B | 5 | 57124334 | A>AACC | 0/1 | 0/0 | 0/0 | 0/0 | 0/0 | ENSBTAT00000017710.5 | c.1344_1345insACC | p.Glu448_Glu449insThr |
| ATP5B | 5 | 57124335 | G>GAC | 0/1 | 0/0 | 0/0 | 0/0 | 0/0 | ENSBTAT00000017710.5 | c.1346_1347insCA | p.Glu449fs |
| **Gene** | **BTA** | **Position** | **Base change** | **Genotype** | | | | | **Transcript** | **cDNA** | **Protein** |
|  |  |  |  | **Case** | **Sire** | **Dam** | **a** | **b** |  |  |  |
| ENSBTAG00000045752 | 5 | 59372236 | TAA>T | 0/1 | 0/0 | 0/0 | 0/0 | 0/0 | ENSBTAT00000064157.1 | c.896_897delTT | p.Phe299fs |
| UHRF1BP1L | 5 | 64433503 | CCT>C | 0/1 | 0/0 | 0/0 | 0/0 | 0/0 | ENSBTAT00000007936.5 | c.3792_3793delAG | p.Gly1265fs |
| UHRF1BP1L | 5 | 64440275 | C>A | 0/1 | 0/0 | 0/0 | 0/0 | 0/0 | ENSBTAT00000007936.5 | c.2201G>T | p.Gly734Val |
| GAS2L3 | 5 | 64853175 | CT>C | 0/1 | 0/0 | 0/0 | 0/0 | 0/0 | ENSBTAT00000018363.5 | c.716delT | p.Leu239fs |
| ENSBTAG00000027899 | 5 | 67925717 | C>T | 0/1 | 0/0 | 0/0 | 0/0 | 0/0 | ENSBTAT00000040243.4 | c.958G>A | p.Asp320Asn |
| FGD4 | 5 | 77673153 | TA>T | 0/1 | 0/0 | 0/0 | 0/0 | 0/0 | ENSBTAT00000007175.5 | c.260delT | p.Leu87fs |
| ENSBTAG00000039041 | 5 | 102725800 | TC>T | 0/1 | 0/0 | 0/0 | 0/0 | 0/0 | ENSBTAT00000056482.2 | c.1723delC | p.His575fs |
| RBP5 | 5 | 103613486 | A>ACGCCATGCCAAGTTACGATCCCTTTGTTGATGCGCTGAGCGAAGCCCGTGAAGCCGCGTGGCGGCACGATACGCTGCTGACCGTCTATGATTATG | 0/1 | 0/0 | 0/0 | 0/0 | 0/0 | ENSBTAT00000010685.4 | c.113_114insCGCCATGCCAAGTTACGATCCCTTTGTTGATGCGCTGAGCGAAGCCCGTGAAGCCGCGTGGCGGCACGATACGCTGCTGACCGTCTATGATTATG | p.Lys38fs |
| P3H3 | 5 | 103972884 | TG>T | 0/1 | 0/0 | 0/0 | 0/0 | 0/0 | ENSBTAT00000037738.4 | c.1803delC | p.Ser602fs |
| LPAR5 | 5 | 104172105 | T>TGGGTATTGCCGCCTTCATGAACTTTGGCGGCTACCATACCTTTGCCGAAACATTCCCGATTGCAGAGGCCGTTGCGGCTAATCG | 0/1 | 0/0 | 0/0 | 0/0 | 0/0 | ENSBTAT00000005968.5 | c.107_108insGGGTATTGCCGCCTTCATGAACTTTGGCGGCTACCATACCTTTGCCGAAACATTCCCGATTGCAGAGGCCGTTGCGGCTAATCG | p.Leu36_Pro37insGlyIleAlaAlaPheMetAsnPheGlyGlyTyrHisThrPheAlaGluThrPheProIleAlaGluAlaValAlaAlaAsnArg |
| VWF | 5 | 104682010 | GC>G | 0/1 | 0/0 | 0/0 | 0/0 | 0/0 | ENSBTAT00000016273.5 | c.5133delC | p.Lys1712fs |
| IQSEC3 | 5 | 107626291 | A>ATTGATGGCTTCCAGCGGGCGCACAGTCAGATGATCCAGCGCGCGCAGCATCAGATTGCCGAGATTGTGGCCATCCAGCTCGCCCTGACCGCTGAAGC | 0/1 | 0/0 | 0/0 | 0/0 | 0/0 | ENSBTAT00000025372.5 | c.2515_2516insTTGATGGCTTCCAGCGGGCGCACAGTCAGATGATCCAGCGCGCGCAGCATCAGATTGCCGAGATTGTGGCCATCCAGCTCGCCCTGACCGCTGAAGC | p.Arg839fs |
| ENSBTAG00000003367 | 5 | 114990168 | A>G | 0/1 | 0/0 | 0/0 | 0/0 | 0/0 | ENSBTAT00000004368.5 | c.95T>C | p.Ile32Thr |
| PARVG | 5 | 115588843 | GA>G | 0/1 | 0/0 | 0/0 | 0/0 | 0/0 | ENSBTAT00000042594.3 | c.377delA | p.Trp126fs |
| **Gene** | **BTA** | **Position** | **Base change** | **Genotype** | | | | | **Transcript** | **cDNA** | **Protein** |
|  |  |  |  | **Case** | **Sire** | **Dam** | **a** | **b** |  |  |  |
| FBLN1 | 5 | 116645561 | GA>G | 0/1 | 0/0 | 0/0 | 0/0 | 0/0 | ENSBTAT00000016040.5 | c.654delA | p.Asp219fs |
| FBLN1 | 5 | 116645564 | G>T | 0/1 | 0/0 | 0/0 | 0/0 | 0/0 | ENSBTAT00000016040.5 | c.655G>T | p.Asp219Tyr |
| GRAMD4 | 5 | 118008590 | A>ACCGGCATATGCAGCCAGCGTTTGAGCATCCGGCTGCCCATCGGCGTCACGGTGCAGTCGAGCACCGACGCCAGCGTATTGTCGAAACCACCGGCCAGGTTCT | 0/1 | 0/0 | 0/0 | 0/0 | 0/0 | ENSBTAT00000029061.4 | c.1285_1286insCCGGCATATGCAGCCAGCGTTTGAGCATCCGGCTGCCCATCGGCGTCACGGTGCAGTCGAGCACCGACGCCAGCGTATTGTCGAAACCACCGGCCAGGTTCT | p.Arg429delinsThrGlyIleCysSerGlnArgLeuSerIleArgLeuProIleGlyValThrValGlnSerSerThrAspAlaSerValLeuSerLysProProAlaArgPheTrp |
| CAMK2D | 6 | 13267802 | A>ACGCCGCTCGCTCCAGTCCAGACAGGCGCAGCATACCCTGCGCCGGTTCCCGGCCGGCAAG | 0/1 | 0/0 | 0/0 | 0/0 | 0/0 | ENSBTAT00000039835.2 | c.1412_1413insCGCCGCTCGCTCCAGTCCAGACAGGCGCAGCATACCCTGCGCCGGTTCCCGGCCGGCAAG | p.Lys471delinsAsnAlaAlaArgSerSerProAspArgArgSerIleProCysAlaGlySerArgProAlaArg |
| ENPEP | 6 | 16130171 | A>ACG | 0/1 | 0/0 | 0/0 | 0/0 | 0/0 | ENSBTAT00000010972.4 | c.877_878insCG | p.Val293fs |
| ENPEP | 6 | 16130174 | G>GAAGTGATAAACCGCAGACCCGCGATCGGCAATATTCGGCCCCAGCGGCTTCATATCCGGTTTCGCCGCGCTTTTTC | 0/1 | 0/0 | 0/0 | 0/0 | 0/0 | ENSBTAT00000010972.4 | c.874_875insGAAAAAGCGCGGCGAAACCGGATATGAAGCCGCTGGGGCCGAATATTGCCGATCGCGGGTCTGCGGTTTATCACTT | p.Ala292fs |
| SMARCAD1 | 6 | 31761953 | A>ATGAGTTCCGCCCGCGCGGAAACCCGAATCTGCGTTCCTGACTGTGCAGGTGATCAAGGAAGCAGAAAATTCATAAAAAATAATCATATCAGTGATTGCACTCCGCCGT | 0/1 | 0/0 | 0/0 | 0/0 | 0/0 | ENSBTAT00000047936.3 | c.1022_1023insACGGCGGAGTGCAATCACTGATATGATTATTTTTTATGAATTTTCTGCTTCCTTGATCACCTGCACAGTCAGGAACGCAGATTCGGGTTTCCGCGCGGGCGGAACTCA | p.Gly341_Phe342insArgArgSerAlaIleThrAspMetIleIlePheTyrGluPheSerAlaSerLeuIleThrCysThrValArgAsnAlaAspSerGlyPheArgAlaGlyGlyThrHis |
| UBA6 | 6 | 85056112 | TA>T | 0/1 | 0/0 | 0/0 | 0/0 | 0/0 | ENSBTAT00000008985.5 | c.2822delT | p.Val941fs |
| ANKRD17 | 6 | 89958077 | T>TGTCG | 0/1 | 0/0 | 0/0 | 0/0 | 0/0 | ENSBTAT00000006467.5 | c.6692_6693insCGAC | p.Pro2232fs |
| ANKRD17 | 6 | 89958078 | A>AGGAACGGCACGCGGTGGATGCGCGCGCTGAGTTTCTCGATTTCCGCCTCGATGTCCTGGATATCGCCGAAGTTGTCCGGCTCTTCACGTTTCAGGCGGGCCAATTCTTCCTTG | 0/1 | 0/0 | 0/0 | 0/0 | 0/0 | ENSBTAT00000006467.5 | c.6691_6692insCAAGGAAGAATTGGCCCGCCTGAAACGTGAAGAGCCGGACAACTTCGGCGATATCCAGGACATCGAGGCGGAAATCGAGAAACTCAGCGCGCGCATCCACCGCGTGCCGTTCC | p.Leu2231fs |
| **Gene** | **BTA** | **Position** | **Base change** | **Genotype** | | | | | **Transcript** | **cDNA** | **Protein** |
|  |  |  |  | **Case** | **Sire** | **Dam** | **a** | **b** |  |  |  |
| ART3 | 6 | 92709211 | A>AGAACGTGCGGCTGACGTGCGTGACATCGGTAAACGTCTTCTGCGCAACATTCTGGACATGGCGATTGTCGATC | 0/1 | 0/0 | 0/0 | 0/0 | 0/0 | ENSBTAT00000054479.2 | c.126_127insGAACGTGCGGCTGACGTGCGTGACATCGGTAAACGTCTTCTGCGCAACATTCTGGACATGGCGATTGTCGATC | p.Cys43fs |
| COPS4 | 6 | 99636951 | TG>T | 0/1 | 0/0 | 0/0 | 0/0 | 0/0 | ENSBTAT00000009128.4 | c.775delG | p.Ala259fs |
| WDFY3 | 6 | 101290151 | G>A | 0/1 | 0/0 | 0/0 | 0/0 | 0/0 | ENSBTAT00000061443.2 | c.10573C>T | p.Arg3525Trp |
| STX18 | 6 | 106365347 | G>GA | 0/1 | 0/0 | 0/0 | 0/0 | 0/0 | ENSBTAT00000007503.5 | c.35_36insA | p.Ser12fs |
| STX18 | 6 | 106365349 | G>GATGGCTGCGCAACATGCCGGCGGTGATCAACAACGCCAGGCTCATCACCAGCAACACGGTGATCAGGGCGATCCC | 0/1 | 0/0 | 0/0 | 0/0 | 0/0 | ENSBTAT00000007503.5 | c.37_38insATGGCTGCGCAACATGCCGGCGGTGATCAACAACGCCAGGCTCATCACCAGCAACACGGTGATCAGGGCGATCCC | p.Val13delinsAspGlyCysAlaThrCysArgArgTerSerThrThrProGlySerSerProAlaThrArgTerSerGlyArgSerLeu |
| DOK7 | 6 | 107252573 | A>AAAAACCGGCAAAGGTCCAGCGGATGGCCTTGTGACCGCGCCAGCCGAGGTGGTGACGGCCCCACAGCAACACGCTGAATACGATCCAGGCCAGGCAGGCCAGCAGG | 0/1 | 0/0 | 0/0 | 0/0 | 0/0 | ENSBTAT00000044179.3 | c.18_19insCCTGCTGGCCTGCCTGGCCTGGATCGTATTCAGCGTGTTGCTGTGGGGCCGTCACCACCTCGGCTGGCGCGGTCACAAGGCCATCCGCTGGACCTTTGCCGGTTTT | p.Cys7fs |
| SLC26A1 | 6 | 109090883 | T>TTGTCATATTCGCCGCACAACCGGTTATGGTTGGCCGCCCCTTCATCACC | 0/1 | 0/0 | 0/0 | 0/0 | 0/0 | ENSBTAT00000009210.2 | c.1615_1616insGGTGATGAAGGGGCGGCCAACCATAACCGGTTGTGCGGCGAATATGACA | p.Glu539fs |
| FLT4 | 7 | 418079 | GC>G | 0/1 | 0/0 | 0/0 | 0/0 | 0/0 | ENSBTAT00000040219.4 | c.280delC | p.His94fs |
| GFPT2 | 7 | 808838 | CG>C | 0/1 | 0/0 | 0/0 | 0/0 | 0/0 | ENSBTAT00000002867.4 | c.1319delG | p.Arg440fs |
| TBC1D9B | 7 | 1302680 | G>GTTC | 0/1 | 0/0 | 0/0 | 0/0 | 0/0 | ENSBTAT00000029796.4 | c.2105_2106insTCT | p.Val702_Ala703insLeu |
| TBC1D9B | 7 | 1302684 | C>CCCACGCCCGGGAGGT | 0/1 | 0/0 | 0/0 | 0/0 | 0/0 | ENSBTAT00000029796.4 | c.2109_2110insCACGCCCGGGAGGTC | p.Ala703_Leu704insHisAlaArgGluVal |
| HAPLN4 | 7 | 3940618 | TG>T | 0/1 | 0/0 | 0/0 | 0/0 | 0/0 | ENSBTAT00000002313.3 | c.504delG | p.Arg169fs |
| HAPLN4 | 7 | 3940620 | CGCG>C | 0/1 | 0/0 | 0/0 | 0/0 | 0/0 | ENSBTAT00000002313.3 | c.506_508delGCG | p.Arg169_Asp170delinsHis |
| **Gene** | **BTA** | **Position** | **Base change** | **Genotype** | | | | | **Transcript** | **cDNA** | **Protein** |
|  |  |  |  | **Case** | **Sire** | **Dam** | **a** | **b** |  |  |  |
| HAPLN4 | 7 | 3940625 | CGGCT>C | 0/1 | 0/0 | 0/0 | 0/0 | 0/0 | ENSBTAT00000002313.3 | c.511_514delGGCT | p.Gly171fs |
| HAPLN4 | 7 | 3940632 | GTGCA>G | 0/1 | 0/0 | 0/0 | 0/0 | 0/0 | ENSBTAT00000002313.3 | c.518_521delTGCA | p.Val173fs |
| NOTCH3 | 7 | 8943273 | G>GACA | 0/1 | 0/0 | 0/0 | 0/0 | 0/0 | ENSBTAT00000060960.2 | c.2176_2177insCAA | p.Trp725_Ser726insThr |
| NOTCH3 | 7 | 8943275 | GT>G | 0/1 | 0/0 | 0/0 | 0/0 | 0/0 | ENSBTAT00000060960.2 | c.2178delT | p.Ser726fs |
| MAST1 | 7 | 13808182 | A>T | 0/1 | 0/0 | 0/0 | 0/0 | 0/0 | ENSBTAT00000007991.5 | c.895T>A | p.Tyr299Asn |
| ENSBTAG00000037693 | 7 | 14365197 | G>A | 0/1 | 0/0 | 0/0 | 0/0 | 0/0 | ENSBTAT00000065546.1 | c.440C>T | p.Thr147Ile |
| ENSBTAG00000046564 | 7 | 15092259 | C>CT | 0/1 | 0/0 | 0/0 | 0/0 | 0/0 | ENSBTAT00000053279.2 | c.534dupT | p.Asn179fs |
| ENSBTAG00000046564 | 7 | 15092262 | A>T | 0/1 | 0/0 | 0/0 | 0/0 | 0/0 | ENSBTAT00000053279.2 | c.536A>T | p.Asn179Ile |
| ENSBTAG00000045635 | 7 | 15660749 | G>C | 0/1 | 0/0 | 0/0 | 0/0 | 0/0 | ENSBTAT00000063762.1 | c.1550C>G | p.Thr517Ser |
| TYK2 | 7 | 16107564 | G>T | 0/1 | 0/0 | 0/0 | 0/0 | 0/0 | ENSBTAT00000015532.5 | c.3078C>A | p.Asn1026Lys |
| TYK2 | 7 | 16107569 | G>GC | 0/1 | 0/0 | 0/0 | 0/0 | 0/0 | ENSBTAT00000015532.5 | c.3072dupG | p.Arg1025fs |
| TYK2 | 7 | 16107571 | G>GGT | 0/1 | 0/0 | 0/0 | 0/0 | 0/0 | ENSBTAT00000015532.5 | c.3070_3071insAC | p.Ala1024fs |
| TYK2 | 7 | 16107579 | G>T | 0/1 | 0/0 | 0/0 | 0/0 | 0/0 | ENSBTAT00000015532.5 | c.3063C>A | p.Asp1021Glu |
| TYK2 | 7 | 16107581 | C>CGATT | 0/1 | 0/0 | 0/0 | 0/0 | 0/0 | ENSBTAT00000015532.5 | c.3060_3061insAATC | p.Asp1021fs |
| TYK2 | 7 | 16107583 | C>T | 0/1 | 0/0 | 0/0 | 0/0 | 0/0 | ENSBTAT00000015532.5 | c.3059G>A | p.Arg1020Gln |
| TYK2 | 7 | 16107587 | G>GTCTTCCATCGCCTGCACAATACGCTCGCGTGGCGT | 0/1 | 0/0 | 0/0 | 0/0 | 0/0 | ENSBTAT00000015532.5 | c.3054_3055insACGCCACGCGAGCGTATTGTGCAGGCGATGGAAGA | p.His1019fs |
| DOCK6 | 7 | 16858547 | TC>T | 0/1 | 0/0 | 0/0 | 0/0 | 0/0 | ENSBTAT00000012587.5 | c.4668delG | p.Lys1557fs |
| DOCK6 | 7 | 16858552 | G>A | 0/1 | 0/0 | 0/0 | 0/0 | 0/0 | ENSBTAT00000012587.5 | c.4664C>T | p.Ala1555Val |
| PRKCSH | 7 | 17043701 | A>G | 0/1 | 0/0 | 0/0 | 0/0 | 0/0 | ENSBTAT00000010787.3 | c.665A>G | p.Asp222Gly |
| **Gene** | **BTA** | **Position** | **Base change** | **Genotype** | | | | | **Transcript** | **cDNA** | **Protein** |
|  |  |  |  | **Case** | **Sire** | **Dam** | **a** | **b** |  |  |  |
| PRKCSH | 7 | 17043705 | C>CGTTGGGGTAACGCCTGCGGTATTCGGCAAATAGCCCGGCGAACAACGTATCACCGGCCAGCAGCGGCAAGCCCAGGCGCAATTCGCCGCGGGCCAGTTGCTGCATGTCGTCCA | 0/1 | 0/0 | 0/0 | 0/0 | 0/0 | ENSBTAT00000010787.3 | c.669_670insGTTGGGGTAACGCCTGCGGTATTCGGCAAATAGCCCGGCGAACAACGTATCACCGGCCAGCAGCGGCAAGCCCAGGCGCAATTCGCCGCGGGCCAGTTGCTGCATGTCGTCCA | p.Met224fs |
| ZNF653 | 7 | 17084941 | G>T | 0/1 | 0/0 | 0/0 | 0/0 | 0/0 | ENSBTAT00000019173.5 | c.119C>A | p.Thr40Lys |
| EVI5L | 7 | 17863200 | A>ACGT | 0/1 | 0/0 | 0/0 | 0/0 | 0/0 | ENSBTAT00000052616.2 | c.2009_2010insCGT | p.Glu670delinsAspVal |
| EVI5L | 7 | 17863203 | T>TCCCACCGGCCAACC | 0/1 | 0/0 | 0/0 | 0/0 | 0/0 | ENSBTAT00000052616.2 | c.2012_2013insCCCACCGGCCAACC | p.Met671fs |
| EVI5L | 7 | 17863207 | G>GTCGCTAT | 0/1 | 0/0 | 0/0 | 0/0 | 0/0 | ENSBTAT00000052616.2 | c.2016_2017insTCGCTAT | p.Gln673fs |
| EVI5L | 7 | 17863210 | G>GGGGCTGTT | 0/1 | 0/0 | 0/0 | 0/0 | 0/0 | ENSBTAT00000052616.2 | c.2019_2020insGGGCTGTT | p.Arg674fs |
| EVI5L | 7 | 17863212 | G>GGCGGAGTATTCGGCCTGGCGACGGTGATCGGGCCGCT | 0/1 | 0/0 | 0/0 | 0/0 | 0/0 | ENSBTAT00000052616.2 | c.2021_2022insGCGGAGTATTCGGCCTGGCGACGGTGATCGGGCCGCT | p.Ile675fs |
| FBN3 | 7 | 18021126 | AC>A | 0/1 | 0/0 | 0/0 | 0/0 | 0/0 | ENSBTAT00000035613.3 | c.6725delG | p.Gly2242fs |
| DPP9 | 7 | 20662815 | AC>A | 0/1 | 0/0 | 0/0 | 0/0 | 0/0 | ENSBTAT00000035885.4 | c.1101delC | p.Asp367fs |
| DPP9 | 7 | 20662817 | G>T | 0/1 | 0/0 | 0/0 | 0/0 | 0/0 | ENSBTAT00000035885.4 | c.1102G>T | p.Gly368Cys |
| APBA3 | 7 | 21441733 | A>G | 0/1 | 0/0 | 0/0 | 0/0 | 0/0 | ENSBTAT00000011048.2 | c.158A>G | p.Asp53Gly |
| APBA3 | 7 | 21441735 | G>A | 0/1 | 0/0 | 0/0 | 0/0 | 0/0 | ENSBTAT00000011048.2 | c.160G>A | p.Gly54Arg |
| THOP1 | 7 | 22131751 | CG>C | 0/1 | 0/0 | 0/0 | 0/0 | 0/0 | ENSBTAT00000027246.5 | c.1371delC | p.Asp458fs |
| SRFBP1 | 7 | 33128657 | T>TTGTTGCCATAAAGGCGTGCCAGTTTGAGCGCCGCTTCGTTG | 0/1 | 0/0 | 0/0 | 0/0 | 0/0 | ENSBTAT00000053358.2 | c.1022_1023insCAACGAAGCGGCGCTCAAACTGGCACGCCTTTATGGCAACA | p.Ser342fs |
| SRFBP1 | 7 | 33128663 | C>CGCCCCCGAGTTA | 0/1 | 0/0 | 0/0 | 0/0 | 0/0 | ENSBTAT00000053358.2 | c.1016_1017insTAACTCGGGGGC | p.Trp339delinsCysAsnSerGlyAla |
| SRFBP1 | 7 | 33128665 | AT>A | 0/1 | 0/0 | 0/0 | 0/0 | 0/0 | ENSBTAT00000053358.2 | c.1014delA | p.Trp339fs |
| SRFBP1 | 7 | 33128669 | A>AAAACACCTTGTCGGCAAAGGT | 0/1 | 0/0 | 0/0 | 0/0 | 0/0 | ENSBTAT00000053358.2 | c.1010_1011insACCTTTGCCGACAAGGTGTTT | p.Pro337_Ser338insProLeuProThrArgCysPhe |
| **Gene** | **BTA** | **Position** | **Base change** | **Genotype** | | | | | **Transcript** | **cDNA** | **Protein** |
|  |  |  |  | **Case** | **Sire** | **Dam** | **a** | **b** |  |  |  |
| FGFR4 | 7 | 39939663 | GCC>G | 0/1 | 0/0 | 0/0 | 0/0 | 0/0 | ENSBTAT00000013942.5 | c.107_108delCC | p.Pro36fs |
| PGBD2 | 7 | 44065797 | G>A | 0/1 | 0/0 | 0/0 | 0/0 | 0/0 | ENSBTAT00000026396.5 | c.658G>A | p.Asp220Asn |
| NEUROG1 | 7 | 48620742 | GTGC>G | 0/1 | 0/0 | 0/0 | 0/0 | 0/0 | ENSBTAT00000004322.4 | c.65_67delGCA | p.Ser22del |
| KDM3B | 7 | 51382463 | GC>G | 0/1 | 0/0 | 0/0 | 0/0 | 0/0 | ENSBTAT00000013278.4 | c.783delC | p.Ser261fs |
| KDM3B | 7 | 51382465 | G>T | 0/1 | 0/0 | 0/0 | 0/0 | 0/0 | ENSBTAT00000013278.4 | c.784G>T | p.Glu262* |
| ENSBTAG00000010871 | 7 | 53392290 | AC>A | 0/1 | 0/0 | 0/0 | 0/0 | 0/0 | ENSBTAT00000014440.5 | c.6354delC | p.Ser2119fs |
| PCDHGB2 | 7 | 54127397 | T>G | 0/1 | 0/0 | 0/0 | 0/0 | 0/0 | ENSBTAT00000057448.1 | c.1630T>G | p.Ser544Ala |
| PCDHGA8 | 7 | 54154407 | G>A | 0/1 | 0/0 | 0/0 | 0/0 | 0/0 | ENSBTAT00000023076.4 | c.1756G>A | p.Val586Ile |
| G3BP1 | 7 | 64995918 | CT>C | 0/1 | 0/0 | 0/0 | 0/0 | 0/0 | ENSBTAT00000027067.5 | c.423delT | p.Phe141fs |
| WWC1 | 7 | 82769096 | C>CGGTCATGAAAAACAGGTTGTAGTACGGCAGTTCACGCATTTGCTTGCTGGCCGCTTCAGCCAGCTCGTCACGGCCATAGCCGATGGCAACACACCACAGGCCGGCCATGCCGTCGAGA | 0/1 | 0/0 | 0/0 | 0/0 | 0/0 | ENSBTAT00000019472.5 | c.2598_2599insGGTCATGAAAAACAGGTTGTAGTACGGCAGTTCACGCATTTGCTTGCTGGCCGCTTCAGCCAGCTCGTCACGGCCATAGCCGATGGCAACACACCACAGGCCGGCCATGCCGTCGAGA | p.Thr867fs |
| RASGRF2 | 7 | 83541718 | A>AG | 0/1 | 0/0 | 0/0 | 0/0 | 0/0 | ENSBTAT00000035048.4 | c.3238_3239insG | p.Thr1080fs |
| RASGRF2 | 7 | 83541725 | A>AG | 0/1 | 0/0 | 0/0 | 0/0 | 0/0 | ENSBTAT00000035048.4 | c.3245_3246insG | p.Gly1083fs |
| RASGRF2 | 7 | 83541727 | GGCC>G | 0/1 | 0/0 | 0/0 | 0/0 | 0/0 | ENSBTAT00000035048.4 | c.3248_3250delGCC | p.Gly1083_Leu1084delinsVal |
| RASGRF2 | 7 | 83541735 | C>CGAG | 0/1 | 0/0 | 0/0 | 0/0 | 0/0 | ENSBTAT00000035048.4 | c.3255_3256insGAG | p.Val1085_Asn1086insGlu |
| RASGRF2 | 7 | 83541737 | A>ACGCTCACTGCGTCG | 0/1 | 0/0 | 0/0 | 0/0 | 0/0 | ENSBTAT00000035048.4 | c.3257_3258insCGCTCACTGCGTCG | p.Phe1087fs |
| RASGRF2 | 7 | 83541740 | T>TA | 0/1 | 0/0 | 0/0 | 0/0 | 0/0 | ENSBTAT00000035048.4 | c.3260_3261insA | p.Phe1087fs |
| RASGRF2 | 7 | 83541742 | T>A | 0/1 | 0/0 | 0/0 | 0/0 | 0/0 | ENSBTAT00000035048.4 | c.3262T>A | p.Ser1088Thr |
| RASGRF2 | 7 | 83541743 | C>G | 0/1 | 0/0 | 0/0 | 0/0 | 0/0 | ENSBTAT00000035048.4 | c.3263C>G | p.Ser1088Cys |
| RASGRF2 | 7 | 83541744 | C>CAACGGA | 0/1 | 0/0 | 0/0 | 0/0 | 0/0 | ENSBTAT00000035048.4 | c.3266_3267insCGGAAA | p.Ser1088_Lys1089insAsnGly |
| **Gene** | **BTA** | **Position** | **Base change** | **Genotype** | | | | | **Transcript** | **cDNA** | **Protein** |
|  |  |  |  | **Case** | **Sire** | **Dam** | **a** | **b** |  |  |  |
| RHOBTB3 | 7 | 97436769 | GC>G | 0/1 | 0/0 | 0/0 | 0/0 | 0/0 | ENSBTAT00000061559.2 | c.1296delC | p.Glu433fs |
| ANXA10 | 8 | 544188 | G>T | 0/1 | 0/0 | 0/0 | 0/0 | 0/0 | ENSBTAT00000006504.5 | c.930G>T | p.Glu310Asp |
| ENSBTAG00000025954 | 8 | 7135634 | G>C | 0/1 | 0/0 | 0/0 | 0/0 | 0/0 | ENSBTAT00000036791.4 | c.1840G>C | p.Ala614Pro |
| ENSBTAG00000025954 | 8 | 7135637 | A>G | 0/1 | 0/0 | 0/0 | 0/0 | 0/0 | ENSBTAT00000036791.4 | c.1843A>G | p.Asn615Asp |
| ENSBTAG00000025954 | 8 | 7135639 | T>A | 0/1 | 0/0 | 0/0 | 0/0 | 0/0 | ENSBTAT00000036791.4 | c.1845T>A | p.Asn615Lys |
| ENSBTAG00000025954 | 8 | 7135643 | A>G | 0/1 | 0/0 | 0/0 | 0/0 | 0/0 | ENSBTAT00000036791.4 | c.1849A>G | p.Ile617Val |
| ENSBTAG00000025954 | 8 | 7135653 | A>G | 0/1 | 0/0 | 0/0 | 0/0 | 0/0 | ENSBTAT00000036791.4 | c.1859A>G | p.Glu620Gly |
| ENSBTAG00000048007 | 8 | 8255112 | G>C | 0/1 | 0/0 | 0/0 | 0/0 | 0/0 | ENSBTAT00000063176.1 | c.382C>G | p.Leu128Val |
| ENSBTAG00000048007 | 8 | 8255114 | A>G | 0/1 | 0/0 | 0/0 | 0/0 | 0/0 | ENSBTAT00000063176.1 | c.380T>C | p.Val127Ala |
| ENSBTAG00000048007 | 8 | 8255116 | G>GCCGGCCAGGTGATCGAGGAAAAGCCGCC | 0/1 | 0/0 | 0/0 | 0/0 | 0/0 | ENSBTAT00000063176.1 | c.377_378insGGCGGCTTTTCCTCGATCACCTGGCCGG | p.Phe126fs |
| RP1L1 | 8 | 8516179 | G>GCACTTCATCCTGCGT | 0/1 | 0/0 | 0/0 | 0/0 | 0/0 | ENSBTAT00000064867.1 | c.4383_4384insCACTTCATCCTGCGT | p.Leu1461_Asp1462insHisPheIleLeuArg |
| RP1L1 | 8 | 8516181 | A>AGCGCG | 0/1 | 0/0 | 0/0 | 0/0 | 0/0 | ENSBTAT00000064867.1 | c.4385_4386insGCGCG | p.Asp1462fs |
| RP1L1 | 8 | 8516184 | A>ATCTTTGGCCCACTGAACGCGACCGTCTTTATCCAGCACCACAATCGCT | 0/1 | 0/0 | 0/0 | 0/0 | 0/0 | ENSBTAT00000064867.1 | c.4388_4389insTCTTTGGCCCACTGAACGCGACCGTCTTTATCCAGCACCACAATCGCT | p.Gln1463delinsHisLeuTrpProThrGluArgAspArgLeuTyrProAlaProGlnSerLeu |
| ENSBTAG00000047195 | 8 | 22729514 | TCC>T | 0/1 | 0/0 | 0/0 | 0/0 | 0/0 | ENSBTAT00000048617.2 | c.382_383delGG | p.Gly128fs |
| ACER2 | 8 | 24834849 | TCA>T | 0/1 | 0/0 | 0/0 | 0/0 | 0/0 | ENSBTAT00000014354.2 | c.507_508delTG | p.Cys169fs |
| HAUS6 | 8 | 25169583 | TA>T | 0/1 | 0/0 | 0/0 | 0/0 | 0/0 | ENSBTAT00000031479.4 | c.916delA | p.Thr306fs |
| NFIB | 8 | 30029990 | T>TCCCGGGTCAATAAATTCAAC | 0/1 | 0/0 | 0/0 | 0/0 | 0/0 | ENSBTAT00000011234.5 | c.253_254insCGGGTCAATAAATTCAACCC | p.Arg85fs |
| NFIB | 8 | 30029994 | C>CTGTCGATCATTCCCACGCTCCGCGTGGGAATGCCGCCTCGGACGCTTCGCGTCCCGATCTTCATGA | 0/1 | 0/0 | 0/0 | 0/0 | 0/0 | ENSBTAT00000011234.5 | c.255_256insTGTCGATCATTCCCACGCTCCGCGTGGGAATGCCGCCTCGGACGCTTCGCGTCCCGATCTTCATGA | p.Arg85_Gln86insCysArgSerPheProArgSerAlaTrpGluCysArgLeuGlyArgPheAlaSerArgSerSerTer |
| **Gene** | **BTA** | **Position** | **Base change** | **Genotype** | | | | | **Transcript** | **cDNA** | **Protein** |
|  |  |  |  | **Case** | **Sire** | **Dam** | **a** | **b** |  |  |  |
| NFIB | 8 | 30029997 | G>GTTTTGTTTCAATCCCGCGTTCAGCTATGCGAT | 0/1 | 0/0 | 0/0 | 0/0 | 0/0 | ENSBTAT00000011234.5 | c.258_259insTTTTGTTTCAATCCCGCGTTCAGCTATGCGAT | p.Glu87fs |
| RIC1 | 8 | 39231886 | TA>T | 0/1 | 0/0 | 0/0 | 0/0 | 0/0 | ENSBTAT00000001692.5 | c.962delT | p.Val321fs |
| ENSBTAG00000045900 | 8 | 60510853 | GC>G | 0/1 | 0/0 | 0/0 | 0/0 | 0/0 | ENSBTAT00000063153.1 | c.615delG | p.Leu205fs |
| DPYSL2 | 8 | 75094769 | T>C | 0/1 | 0/0 | 0/0 | 0/0 | 0/0 | ENSBTAT00000024448.4 | c.173T>C | p.Ile58Thr |
| SEMA4D | 8 | 90235110 | G>T | 0/1 | 0/0 | 0/0 | 0/0 | 0/0 | ENSBTAT00000002525.5 | c.547G>T | p.Gly183* |
| FKBP15 | 8 | 104119468 | T>TG | 0/1 | 0/0 | 0/0 | 0/0 | 0/0 | ENSBTAT00000006746.5 | c.2509dupC | p.Gln837fs |
| FKBP15 | 8 | 104119470 | C>CCGCGCGCAAAAGCGCCGATCCTGCCAGCTCCTACACGGCGAAGCTCTATGCCAGCGGCACCAAGCGCATCGCCCAGAAGGTGGG | 0/1 | 0/0 | 0/0 | 0/0 | 0/0 | ENSBTAT00000006746.5 | c.2507_2508insCCCACCTTCTGGGCGATGCGCTTGGTGCCGCTGGCATAGAGCTTCGCCGTGTAGGAGCTGGCAGGATCGGCGCTTTTGCGCGCG | p.Gln836delinsHisProProSerGlyArgCysAlaTrpCysArgTrpHisArgAlaSerProCysArgSerTrpGlnAspArgArgPheCysAlaArg |
| C5 | 8 | 112298389 | T>TCACGTCGATGGCGTCAATC | 0/1 | 0/0 | 0/0 | 0/0 | 0/0 | ENSBTAT00000016204.5 | c.2205_2206insGATTGACGCCATCGACGTG | p.Ser736fs |
| C5 | 8 | 112298392 | C>CAACGCTCGCGGGT | 0/1 | 0/0 | 0/0 | 0/0 | 0/0 | ENSBTAT00000016204.5 | c.2202_2203insACCCGCGAGCGTT | p.Ala735fs |
| C5 | 8 | 112298394 | A>ATTCTGATGCTGGTGACGGCGC | 0/1 | 0/0 | 0/0 | 0/0 | 0/0 | ENSBTAT00000016204.5 | c.2200_2201insGCGCCGTCACCAGCATCAGAA | p.Ala733_Ile734insSerAlaValThrSerIleArg |
| LCA5 | 9 | 19363114 | TAG>T | 0/1 | 0/0 | 0/0 | 0/0 | 0/0 | ENSBTAT00000061609.2 | c.212_213delCT | p.Pro71fs |
| TTK | 9 | 19988194 | C>T | 0/1 | 0/0 | 0/0 | 0/0 | 0/0 | ENSBTAT00000066037.1 | c.2215C>T | p.Gln739* |
| TTK | 9 | 19988204 | C>T | 0/1 | 0/0 | 0/0 | 0/0 | 0/0 | ENSBTAT00000066037.1 | c.2225C>T | p.Pro742Leu |
| RWDD1 | 9 | 34581963 | TA>T | 0/1 | 0/0 | 0/0 | 0/0 | 0/0 | ENSBTAT00000012779.5 | c.308delT | p.Leu103fs |
| CDK19 | 9 | 40117646 | TC>T | 0/1 | 0/0 | 0/0 | 0/0 | 0/0 | ENSBTAT00000009583.4 | c.1104delC | p.Asn369fs |
| ENSBTAG00000045624 | 9 | 49838975 | C>T | 0/1 | 0/0 | 0/0 | 0/0 | 0/0 | ENSBTAT00000064018.1 | c.979C>T | p.Arg327Trp |
| ENSBTAG00000045624 | 9 | 49838985 | A>T | 0/1 | 0/0 | 0/0 | 0/0 | 0/0 | ENSBTAT00000064018.1 | c.989A>T | p.Asp330Val |
| KLHL32 | 9 | 53392490 | TGACAG>T | 0/1 | 0/0 | 0/0 | 0/0 | 0/0 | ENSBTAT00000006977.4 | c.951_955delCTGTC | p.Cys318fs |
| SAMD3 | 9 | 69309745 | AG>A | 0/1 | 0/0 | 0/0 | 0/0 | 0/0 | ENSBTAT00000028479.4 | c.218delC | p.Pro73fs |
| **Gene** | **BTA** | **Position** | **Base change** | **Genotype** | | | | | **Transcript** | **cDNA** | **Protein** |
|  |  |  |  | **Case** | **Sire** | **Dam** | **a** | **b** |  |  |  |
| TMEM200A | 9 | 69513990 | GCT>G | 0/1 | 0/0 | 0/0 | 0/0 | 0/0 | ENSBTAT00000053029.2 | c.743_744delCT | p.Ser248fs |
| HIVEP2 | 9 | 81266176 | TG>T | 0/1 | 0/0 | 0/0 | 0/0 | 0/0 | ENSBTAT00000001526.4 | c.317delC | p.Pro106fs |
| SYNE1 | 9 | 90384595 | C>CGCACTCGGCTCGTGGTCCAGCTTATGGGACAACTCACGTGCGGTACGCAGATAGACGTTCAGCTCTTTGACAATATGGATAGGTAAACGGATGGTACGGGTTTGGTTCATG | 0/1 | 0/0 | 0/0 | 0/0 | 0/0 | ENSBTAT00000012328.5 | c.21551_21552insCATGAACCAAACCCGTACCATCCGTTTACCTATCCATATTGTCAAAGAGCTGAACGTCTATCTGCGTACCGCACGTGAGTTGTCCCATAAGCTGGACCACGAGCCGAGTGC | p.Leu7184_Glu7185insMetAsnGlnThrArgThrIleArgLeuProIleHisIleValLysGluLeuAsnValTyrLeuArgThrAlaArgGluLeuSerHisLysLeuAspHisGluProSerAla |
| PNLDC1 | 9 | 97506537 | TA>T | 0/1 | 0/0 | 0/0 | 0/0 | 0/0 | ENSBTAT00000020180.4 | c.255delA | p.Ala86fs |
| FRMD1 | 9 | 103992925 | T>A | 0/1 | 0/0 | 0/0 | 0/0 | 0/0 | ENSBTAT00000018684.5 | c.668A>T | p.Tyr223Phe |
| FAM120B | 9 | 105546041 | TGCG>T | 0/1 | 0/0 | 0/0 | 0/0 | 0/0 | ENSBTAT00000005065.5 | c.204_206delCGG | p.Gly69del |
| IGDCC4 | 10 | 12277850 | C>CGCGCAGACCCAAATCGCGCAAACCATCGAGCAGGCCGAGTACCAGAAAATCGCTGGCGGCGAACGCGGCGGTCGGGCGTTTTGTGTGGCTGAACAGCTTGTGCAAGGCG | 0/1 | 0/0 | 0/0 | 0/0 | 0/0 | ENSBTAT00000008442.4 | c.975_976insCGCCTTGCACAAGCTGTTCAGCCACACAAAACGCCCGACCGCCGCGTTCGCCGCCAGCGATTTTCTGGTACTCGGCCTGCTCGATGGTTTGCGCGATTTGGGTCTGCGC | p.Ala326fs |
| NFATC4 | 10 | 20639302 | C>CCGA | 0/1 | 0/0 | 0/0 | 0/0 | 0/0 | ENSBTAT00000039158.3 | c.412_413insTCG | p.Ser138delinsIleGly |
| NFATC4 | 10 | 20639304 | G>GACCACCGTGGGCGCCTCGGGCTGGGCCTGTCGTCGGTGATCGCCGACAACCTGCGCGGCCGC | 0/1 | 0/0 | 0/0 | 0/0 | 0/0 | ENSBTAT00000039158.3 | c.410_411insGCGGCCGCGCAGGTTGTCGGCGATCACCGACGACAGGCCCAGCCCGAGGCGCCCACGGTGGT | p.Ser138fs |
| RNF212B | 10 | 21477771 | G>A | 0/1 | 0/0 | 0/0 | 0/0 | 0/0 | ENSBTAT00000036401.1 | c.617C>T | p.Pro206Leu |
| OSGEP | 10 | 26715515 | T>G | 0/1 | 0/0 | 0/0 | 0/0 | 0/0 | ENSBTAT00000003557.2 | c.805T>G | p.Leu269Val |
| ZNF106 | 10 | 37913373 | TC>T | 0/1 | 0/0 | 0/0 | 0/0 | 0/0 | ENSBTAT00000005329.5 | c.1702delG | p.Glu568fs |
| TTBK2 | 10 | 38163220 | T>A | 0/1 | 0/0 | 0/0 | 0/0 | 0/0 | ENSBTAT00000008483.2 | c.2336A>T | p.Glu779Val |
| SNX1 | 10 | 45933155 | GA>G | 0/1 | 0/0 | 0/0 | 0/0 | 0/0 | ENSBTAT00000002609.4 | c.7delT | p.Ser3fs |
| **Gene** | **BTA** | **Position** | **Base change** | **Genotype** | | | | | **Transcript** | **cDNA** | **Protein** |
|  |  |  |  | **Case** | **Sire** | **Dam** | **a** | **b** |  |  |  |
| FAM63B | 10 | 51570864 | C>CACGGTGGCAAAAGGCGGGCTGGCGACCACGTTGCTCTGATCGGCATACTTCTCGTTAAAATTCTCCTGCTTCCAGGCCAG | 0/1 | 0/0 | 0/0 | 0/0 | 0/0 | ENSBTAT00000016444.4 | c.45_46insCTGGCCTGGAAGCAGGAGAATTTTAACGAGAAGTATGCCGATCAGAGCAACGTGGTCGCCAGCCCGCCTTTTGCCACCGT | p.Ala16fs |
| PRTG | 10 | 54834913 | C>A | 0/1 | 0/0 | 0/0 | 0/0 | 0/0 | ENSBTAT00000061599.2 | c.2624C>A | p.Thr875Asn |
| USP8 | 10 | 59988219 | TTG>T | 0/1 | 0/0 | 0/0 | 0/0 | 0/0 | ENSBTAT00000033814.4 | c.2721_2722delCA | p.His907fs |
| DAAM1 | 10 | 71868264 | T>G | 0/1 | 0/0 | 0/0 | 0/0 | 0/0 | ENSBTAT00000007855.5 | c.779T>G | p.Leu260* |
| PCNX4 | 10 | 72587244 | A>T | 0/1 | 0/0 | 0/0 | 0/0 | 0/0 | ENSBTAT00000018737.5 | c.1691A>T | p.Glu564Val |
| ESR2 | 10 | 76737875 | TG>T | 0/1 | 0/0 | 0/0 | 0/0 | 0/0 | ENSBTAT00000005899.4 | c.846delC | p.Ser283fs |
| SIPA1L1 | 10 | 83589287 | C>CGCTGGCGAGTTCATCGCGCTGGTGCGTCGACTTGATCTCGGCGGGCAACTCAAACAGCAATTGAGTGATGCGCTGCGCGGCAA | 0/1 | 0/0 | 0/0 | 0/0 | 0/0 | ENSBTAT00000035650.4 | c.4005_4006insGCTGGCGAGTTCATCGCGCTGGTGCGTCGACTTGATCTCGGCGGGCAACTCAAACAGCAATTGAGTGATGCGCTGCGCGGCAA | p.Ser1336fs |
| PAPLN | 10 | 85116529 | TG>T | 0/1 | 0/0 | 0/0 | 0/0 | 0/0 | ENSBTAT00000015505.5 | c.2331delG | p.Trp777fs |
| SNW1 | 10 | 89923841 | CT>C | 0/1 | 0/0 | 0/0 | 0/0 | 0/0 | ENSBTAT00000010697.5 | c.1427delA | p.Lys476fs |
| ALMS1 | 11 | 10963963 | T>A | 0/1 | 0/0 | 0/0 | 0/0 | 0/0 | ENSBTAT00000047267.3 | c.5169A>T | p.Leu1723Phe |
| ALMS1 | 11 | 10963970 | T>C | 0/1 | 0/0 | 0/0 | 0/0 | 0/0 | ENSBTAT00000047267.3 | c.5164-2A>G |  |
| EIF2AK2 | 11 | 19591562 | TC>T | 0/1 | 0/0 | 0/0 | 0/0 | 0/0 | ENSBTAT00000011469.3 | c.1357delG | p.Asp453fs |
| SPTBN1 | 11 | 37196647 | TC>T | 0/1 | 0/0 | 0/0 | 0/0 | 0/0 | ENSBTAT00000009208.5 | c.1339delC | p.Gln447fs |
| IL36B | 11 | 46579117 | A>T | 0/1 | 0/0 | 0/0 | 0/0 | 0/0 | ENSBTAT00000002696.4 | c.99A>T | p.Glu33Asp |
| RNF103 | 11 | 48149398 | TG>T | 0/1 | 0/0 | 0/0 | 0/0 | 0/0 | ENSBTAT00000027899.5 | c.92delG | p.Gly31fs |
| SPDYA | 11 | 70937490 | A>ACAAACGACCTGCTAGACCAGGTCGGCACGTGTCCAAATGTGTCTGAACCGTTGTGTCGTCACACTAGACCCACGCAGTGATAAAC | 0/1 | 0/0 | 0/0 | 0/0 | 0/0 | ENSBTAT00000044419.3 | c.397_398insGTTTATCACTGCGTGGGTCTAGTGTGACGACACAACGGTTCAGACACATTTGGACACGTGCCGACCTGGTCTAGCAGGTCGTTTG | p.Phe133fs |
| **Gene** | **BTA** | **Position** | **Base change** | **Genotype** | | | | | **Transcript** | **cDNA** | **Protein** |
|  |  |  |  | **Case** | **Sire** | **Dam** | **a** | **b** |  |  |  |
| CAD | 11 | 72394484 | T>TGTTGGTCAGGTTGAAGACGCCGTCCACGGACAGCCCCGCGCGGAACGCATCG | 0/1 | 0/0 | 0/0 | 0/0 | 0/0 | ENSBTAT00000023783.5 | c.2496_2497insCGATGCGTTCCGCGCGGGGCTGTCCGTGGACGGCGTCTTCAACCTGACCAAC | p.Ile833fs |
| CAD | 11 | 72394488 | AG>A | 0/1 | 0/0 | 0/0 | 0/0 | 0/0 | ENSBTAT00000023783.5 | c.2492delC | p.Thr831fs |
| CAD | 11 | 72394491 | G>GTACC | 0/1 | 0/0 | 0/0 | 0/0 | 0/0 | ENSBTAT00000023783.5 | c.2489_2490insGGTA | p.Thr831fs |
| CAD | 11 | 72394493 | G>GATACGCTCGGCGCCGGCATCTTTCAGCTCACGGCGAATT | 0/1 | 0/0 | 0/0 | 0/0 | 0/0 | ENSBTAT00000023783.5 | c.2487_2488insAATTCGCCGTGAGCTGAAAGATGCCGGCGCCGAGCGTAT | p.Glu829_Leu830insAsnSerProTerAlaGluArgCysArgArgArgAlaTyr |
| ADCY3 | 11 | 74446192 | AC>A | 0/1 | 0/0 | 0/0 | 0/0 | 0/0 | ENSBTAT00000032950.3 | c.1268delC | p.Thr423fs |
| NCOA1 | 11 | 74569910 | T>C | 0/1 | 0/0 | 0/0 | 0/0 | 0/0 | ENSBTAT00000064008.1 | c.455A>G | p.Tyr152Cys |
| WDCP | 11 | 75073157 | T>A | 0/1 | 0/0 | 0/0 | 0/0 | 0/0 | ENSBTAT00000022591.4 | c.1479T>A | p.Ser493Arg |
| ATP6V1C2 | 11 | 86867144 | CT>C | 0/1 | 0/0 | 0/0 | 0/0 | 0/0 | ENSBTAT00000032875.3 | c.812delA | p.Lys271fs |
| TAF1B | 11 | 87634182 | A>G | 0/1 | 0/0 | 0/0 | 0/0 | 0/0 | ENSBTAT00000043676.1 | c.1658T>C | p.Ile553Thr |
| HMCN2 | 11 | 100746840 | AC>A | 0/1 | 0/0 | 0/0 | 0/0 | 0/0 | ENSBTAT00000007111.5 | c.11055delC | p.Thr3686fs |
| TTF1 | 11 | 102498930 | A>G | 0/1 | 0/0 | 0/0 | 0/0 | 0/0 | ENSBTAT00000024905.5 | c.1606T>C | p.Ser536Pro |
| ABO | 11 | 104232542 | C>T | 0/1 | 0/0 | 0/0 | 0/0 | 0/0 | ENSBTAT00000042758.3 | c.838G>A | p.Gly280Arg |
| GRIN1 | 11 | 105937555 | G>GACGGTCGAATTTCTCGCGCTCGGCAGAAATCAGCCCGCAGGAAAGCCCAAACCGGGTGGCATTCGCGAGGGTAAT | 0/1 | 0/0 | 0/0 | 0/0 | 0/0 | ENSBTAT00000065439.1 | c.681_682insATTACCCTCGCGAATGCCACCCGGTTTGGGCTTTCCTGCGGGCTGATTTCTGCCGAGCGCGAGAAATTCGACCGT | p.Ala227_Leu228insIleThrLeuAlaAsnAlaThrArgPheGlyLeuSerCysGlyLeuIleSerAlaGluArgGluLysPheAspArg |
| GRIN1 | 11 | 105937559 | C>CTCATCAAAATCGTCATAGCGCCAGACGCT | 0/1 | 0/0 | 0/0 | 0/0 | 0/0 | ENSBTAT00000065439.1 | c.677_678insAGCGTCTGGCGCTATGACGATTTTGATGA | p.Leu228fs |
| CCDC183 | 11 | 106370402 | G>GGC | 0/1 | 0/0 | 0/0 | 0/0 | 0/0 | ENSBTAT00000042795.2 | c.522_523insGC | p.Leu175fs |
| **Gene** | **BTA** | **Position** | **Base change** | **Genotype** | | | | | **Transcript** | **cDNA** | **Protein** |
|  |  |  |  | **Case** | **Sire** | **Dam** | **a** | **b** |  |  |  |
| CCDC183 | 11 | 106370404 | T>TCCCGGGGTACCCACAACAACTTGTGGACCTTGACGCAGTGCGCGCAGCTGAACGTCATAGCGCTGG | 0/1 | 0/0 | 0/0 | 0/0 | 0/0 | ENSBTAT00000042795.2 | c.520_521insCCAGCGCTATGACGTTCAGCTGCGCGCACTGCGTCAAGGTCCACAAGTTGTTGTGGGTACCCCGGG | p.Met173_Asp174insAlaSerAlaMetThrPheSerCysAlaHisCysValLysValHisLysLeuLeuTrpValProArg |
| TNFSF11 | 12 | 12748575 | CTG>C | 0/1 | 0/0 | 0/0 | 0/0 | 0/0 | ENSBTAT00000011745.4 | c.308_309delTG | p.Leu103fs |
| CYSLTR2 | 12 | 18528001 | TC>T | 0/1 | 0/0 | 0/0 | 0/0 | 0/0 | ENSBTAT00000023275.3 | c.213delC | p.Thr72fs |
| CDADC1 | 12 | 18974948 | A>G | 0/1 | 0/0 | 0/0 | 0/0 | 0/0 | ENSBTAT00000002932.5 | c.556A>G | p.Ser186Gly |
| SMAD9 | 12 | 24794932 | AC>A | 0/1 | 0/0 | 0/0 | 0/0 | 0/0 | ENSBTAT00000009982.5 | c.419delC | p.Thr140fs |
| N4BP2L2 | 12 | 28560344 | ACC>A | 0/1 | 0/0 | 0/0 | 0/0 | 0/0 | ENSBTAT00000009870.4 | c.1651_1652delCC | p.Pro551fs |
| SHISA2 | 12 | 33578352 | G>GGTCACCGGTCTTATCGTCAGCCTGTTGCAGGCGGCGACGCAAATCAACGAACAAACGCTGTCCTTCATTCCGAAGATC | 0/1 | 0/0 | 0/0 | 0/0 | 0/0 | ENSBTAT00000024243.4 | c.60_61insGTCACCGGTCTTATCGTCAGCCTGTTGCAGGCGGCGACGCAAATCAACGAACAAACGCTGTCCTTCATTCCGAAGATC | p.Leu20_Leu21insValThrGlyLeuIleValSerLeuLeuGlnAlaAlaThrGlnIleAsnGluGlnThrLeuSerPheIleProLysIle |
| ENSBTAG00000032603 | 12 | 70048477 | T>G | 0/1 | 0/0 | 0/0 | 0/0 | 0/0 | ENSBTAT00000013298.5 | c.3226A>C | p.Lys1076Gln |
| ENSBTAG00000032603 | 12 | 70048480 | C>CTG | 0/1 | 0/0 | 0/0 | 0/0 | 0/0 | ENSBTAT00000013298.5 | c.3222_3223insCA | p.Gly1075fs |
| ENSBTAG00000032603 | 12 | 70048482 | TCA>T | 0/1 | 0/0 | 0/0 | 0/0 | 0/0 | ENSBTAT00000013298.5 | c.3219_3220delTG | p.Glu1074fs |
| ENSBTAG00000046041 | 12 | 73617686 | G>A | 0/1 | 0/0 | 0/0 | 0/0 | 0/0 | ENSBTAT00000030481.4 | c.1588G>A | p.Glu530Lys |
| ENSBTAG00000026070 | 12 | 75487893 | T>G | 0/1 | 0/0 | 0/0 | 0/0 | 0/0 | ENSBTAT00000036965.4 | c.1034T>G | p.Leu345Arg |
| ENSBTAG00000039714 | 12 | 75686694 | G>A | 0/1 | 0/0 | 0/0 | 0/0 | 0/0 | ENSBTAT00000035484.4 | c.616G>A | p.Asp206Asn |
| ENSBTAG00000039714 | 12 | 75686700 | G>A | 0/1 | 0/0 | 0/0 | 0/0 | 0/0 | ENSBTAT00000035484.4 | c.621+1G>A |  |
| **ZIC2** | **12** | **80722845** | **TC>T** | **0/1** | **0/0** | **0/0** | **0/0** | **0/0** | **ENSBTAT00000035414.4** | **c.1628delC** | **p.Pro543fs** |
| MCF2L | 12 | 90486528 | A>AGCTGCATT | 0/1 | 0/0 | 0/0 | 0/0 | 0/0 | ENSBTAT00000035322.3 | c.1017_1018insTGCATTGC | p.Leu340fs |
| MCF2L | 12 | 90486533 | G>GTCGGATTAATCGCCGTCTCGCGCCGCTGTCCGAGCCGCT | 0/1 | 0/0 | 0/0 | 0/0 | 0/0 | ENSBTAT00000035322.3 | c.1020_1021insTCGGATTAATCGCCGTCTCGCGCCGCTGTCCGAGCCGCT | p.Leu340_Ala341insSerAspTerSerProSerArgAlaAlaValArgAlaAla |
| MCF2L | 12 | 90486536 | A>AAAGCGGCTCCACGGTGGAGATCGTCAGCGCACCGGGCG | 0/1 | 0/0 | 0/0 | 0/0 | 0/0 | ENSBTAT00000035322.3 | c.1023_1024insAAGCGGCTCCACGGTGGAGATCGTCAGCGCACCGGGCG | p.His342fs |
| **Gene** | **BTA** | **Position** | **Base change** | **Genotype** | | | | | **Transcript** | **cDNA** | **Protein** |
|  |  |  |  | **Case** | **Sire** | **Dam** | **a** | **b** |  |  |  |
| JAG1 | 13 | 3835850 | TG>T | 0/1 | 0/0 | 0/0 | 0/0 | 0/0 | ENSBTAT00000009631.5 | c.2925delC | p.Thr976fs |
| MASTL | 13 | 18030312 | G>GCCATACAGCGGCAGTTCGGCGAGCGCGCTGTCGATTTTCTGCTCTTCGCTTTCGCCCATGACGCGCGGCTTAATCACCCCGCCCCACACTATTGCGCCGCCCACAATCAGCAC | 0/1 | 0/0 | 0/0 | 0/0 | 0/0 | ENSBTAT00000022950.5 | c.1681_1682insCCATACAGCGGCAGTTCGGCGAGCGCGCTGTCGATTTTCTGCTCTTCGCTTTCGCCCATGACGCGCGGCTTAATCACCCCGCCCCACACTATTGCGCCGCCCACAATCAGCAC | p.Asp561fs |
| ZNF438 | 13 | 34806472 | CCAT>C | 0/1 | 0/0 | 0/0 | 0/0 | 0/0 | ENSBTAT00000021674.5 | c.2262_2264delTCA | p.His754del |
| ADARB2 | 13 | 46569246 | TC>T | 0/1 | 0/0 | 0/0 | 0/0 | 0/0 | ENSBTAT00000018870.4 | c.207delC | p.Lys70fs |
| SIGLEC1 | 13 | 51959450 | CG>C | 0/1 | 0/0 | 0/0 | 0/0 | 0/0 | ENSBTAT00000017523.4 | c.4582delG | p.Val1528fs |
| C20orf194 | 13 | 52331923 | CAA>C | 0/1 | 0/0 | 0/0 | 0/0 | 0/0 | ENSBTAT00000061472.2 | c.901_902delAA | p.Asn301fs |
| PRPF6 | 13 | 54308754 | C>CGCAACCGTGGTGCGCGCCAGTCGTCGTTTAGGCTTCGAAGGCTATCCGGCGCTGCGGCTGGCGTTAGCCGCTGAACTGCCGCGCAATGCCCCGGCGGGAGAACCGCTGGTGGCCGACATCGGCG | 0/1 | 0/0 | 0/0 | 0/0 | 0/0 | ENSBTAT00000015480.2 | c.2789_2790insCGCCGATGTCGGCCACCAGCGGTTCTCCCGCCGGGGCATTGCGCGGCAGTTCAGCGGCTAACGCCAGCCGCAGCGCCGGATAGCCTTCGAAGCCTAAACGACGACTGGCGCGCACCACGGTTGC | p.Val931fs |
| DNAJC5 | 13 | 54383679 | ACTCACCCCTCTCATC>A | 0/1 | 0/0 | 0/0 | 0/0 | 0/0 | ENSBTAT00000039334.3 | c.484_493+5delGATGAGAGGGGTGAG | p.Asp162fs |
| DNAJC5 | 13 | 54383697 | ACT>A | 0/1 | 0/0 | 0/0 | 0/0 | 0/0 | ENSBTAT00000039334.3 | c.479_480delAG | p.Gln160fs |
| DNAJC5 | 13 | 54383700 | GC>G | 0/1 | 0/0 | 0/0 | 0/0 | 0/0 | ENSBTAT00000039334.3 | c.477delG | p.Gln160fs |
| DNAJC5 | 13 | 54383705 | T>A | 0/1 | 0/0 | 0/0 | 0/0 | 0/0 | ENSBTAT00000039334.3 | c.473A>T | p.Gln158Leu |
| DNAJC5 | 13 | 54383708 | GCC>G | 0/1 | 0/0 | 0/0 | 0/0 | 0/0 | ENSBTAT00000039334.3 | c.468_469delGG | p.Glu156fs |
| DNAJC5 | 13 | 54383713 | CA>C | 0/1 | 0/0 | 0/0 | 0/0 | 0/0 | ENSBTAT00000039334.3 | c.464delT | p.Leu155fs |
| YTHDF1 | 13 | 54812938 | A>T | 0/1 | 0/0 | 0/0 | 0/0 | 0/0 | ENSBTAT00000013703.5 | c.1136A>T | p.Tyr379Phe |
| **Gene** | **BTA** | **Position** | **Base change** | **Genotype** | | | | | **Transcript** | **cDNA** | **Protein** |
|  |  |  |  | **Case** | **Sire** | **Dam** | **a** | **b** |  |  |  |
| YTHDF1 | 13 | 54812942 | C>CCTGCTGGACGCGCGTAAAGCCATCTCCGTCACCGAGCGTCAGCGCTACATTCTGCGCATTCGCACCCTGACCAAAGCCGTGGCAGAAGCGTACTACGCTTCCCGTGAAG | 0/1 | 0/0 | 0/0 | 0/0 | 0/0 | ENSBTAT00000013703.5 | c.1141_1142insTGCTGGACGCGCGTAAAGCCATCTCCGTCACCGAGCGTCAGCGCTACATTCTGCGCATTCGCACCCTGACCAAAGCCGTGGCAGAAGCGTACTACGCTTCCCGTGAAGC | p.Pro381fs |
| CABLES2 | 13 | 55346131 | TGG>T | 0/1 | 0/0 | 0/0 | 0/0 | 0/0 | ENSBTAT00000027714.4 | c.85_86delGG | p.Gly29fs |
| CABLES2 | 13 | 55346133 | G>GTTC | 0/1 | 0/0 | 0/0 | 0/0 | 0/0 | ENSBTAT00000027714.4 | c.86_87insTTC | p.Gly29_Ala30insSer |
| CABLES2 | 13 | 55346135 | G>GCCA | 0/1 | 0/0 | 0/0 | 0/0 | 0/0 | ENSBTAT00000027714.4 | c.89_90insCAC | p.Ala30_Pro31insThr |
| CABLES2 | 13 | 55346141 | G>T | 0/1 | 0/0 | 0/0 | 0/0 | 0/0 | ENSBTAT00000027714.4 | c.94G>T | p.Gly32Cys |
| CABLES2 | 13 | 55346145 | C>G | 0/1 | 0/0 | 0/0 | 0/0 | 0/0 | ENSBTAT00000027714.4 | c.98C>G | p.Thr33Arg |
| CABLES2 | 13 | 55346147 | TCCTCC>T | 0/1 | 0/0 | 0/0 | 0/0 | 0/0 | ENSBTAT00000027714.4 | c.101_105delCCTCC | p.Ser34fs |
| CABLES2 | 13 | 55346154 | GCCCC>G | 0/1 | 0/0 | 0/0 | 0/0 | 0/0 | ENSBTAT00000027714.4 | c.108_111delCCCC | p.Thr38fs |
| CABLES2 | 13 | 55346159 | A>ATG | 0/1 | 0/0 | 0/0 | 0/0 | 0/0 | ENSBTAT00000027714.4 | c.112_113insTG | p.Thr38fs |
| CABLES2 | 13 | 55346163 | C>A | 0/1 | 0/0 | 0/0 | 0/0 | 0/0 | ENSBTAT00000027714.4 | c.116C>A | p.Ala39Asp |
| CABLES2 | 13 | 55346164 | CA>C | 0/1 | 0/0 | 0/0 | 0/0 | 0/0 | ENSBTAT00000027714.4 | c.118delA | p.Arg40fs |
| CABLES2 | 13 | 55346171 | G>GCA | 0/1 | 0/0 | 0/0 | 0/0 | 0/0 | ENSBTAT00000027714.4 | c.125_126insAC | p.Pro43fs |
| CABLES2 | 13 | 55346176 | A>ATT | 0/1 | 0/0 | 0/0 | 0/0 | 0/0 | ENSBTAT00000027714.4 | c.129_130insTT | p.Gln44fs |
| CABLES2 | 13 | 55346177 | C>G | 0/1 | 0/0 | 0/0 | 0/0 | 0/0 | ENSBTAT00000027714.4 | c.130C>G | p.Gln44Glu |
| CABLES2 | 13 | 55346180 | G>GATATTC | 0/1 | 0/0 | 0/0 | 0/0 | 0/0 | ENSBTAT00000027714.4 | c.133_134insATATTC | p.Ala45delinsAspIlePro |
| RAD21L1 | 13 | 60423318 | A>G | 0/1 | 0/0 | 0/0 | 0/0 | 0/0 | ENSBTAT00000001812.5 | c.1037T>C | p.Ile346Thr |
| RAD21L1 | 13 | 60423324 | G>A | 0/1 | 0/0 | 0/0 | 0/0 | 0/0 | ENSBTAT00000001812.5 | c.1031C>T | p.Ala344Val |
| RAD21L1 | 13 | 60423402 | G>C | 0/1 | 0/0 | 0/0 | 0/0 | 0/0 | ENSBTAT00000001812.5 | c.953C>G | p.Ser318Cys |
| HCK | 13 | 62128084 | TC>T | 0/1 | 0/0 | 0/0 | 0/0 | 0/0 | ENSBTAT00000056914.1 | c.216delC | p.Thr73fs |
| ENSBTAG00000011704 | 13 | 63150970 | TG>T | 0/1 | 0/0 | 0/0 | 0/0 | 0/0 | ENSBTAT00000015544.3 | c.315delG | p.Tyr106fs |
| **Gene** | **BTA** | **Position** | **Base change** | **Genotype** | | | | | **Transcript** | **cDNA** | **Protein** |
|  |  |  |  | **Case** | **Sire** | **Dam** | **a** | **b** |  |  |  |
| ENSBTAG00000011704 | 13 | 63150973 | ACCCTTCGAGAATTT>A | 0/1 | 0/0 | 0/0 | 0/0 | 0/0 | ENSBTAT00000015544.3 | c.318_331delCCCTTCGAGAATTT | p.Tyr106fs |
| KIAA1755 | 13 | 67769247 | AT>A | 0/1 | 0/0 | 0/0 | 0/0 | 0/0 | ENSBTAT00000025819.5 | c.1322delA | p.Asn441fs |
| KIAA1755 | 13 | 67769249 | T>A | 0/1 | 0/0 | 0/0 | 0/0 | 0/0 | ENSBTAT00000025819.5 | c.1321A>T | p.Asn441Tyr |
| JPH2 | 13 | 73349370 | GC>G | 0/1 | 0/0 | 0/0 | 0/0 | 0/0 | ENSBTAT00000061391.2 | c.1133delG | p.Arg378fs |
| JPH2 | 13 | 73349372 | G>T | 0/1 | 0/0 | 0/0 | 0/0 | 0/0 | ENSBTAT00000061391.2 | c.1132C>A | p.Arg378Ser |
| KCNS1 | 13 | 74195571 | G>GCGGGCGCGGCATTGGCCGAGATCACTCACGCCGTGGCACAAATGCGCGA | 0/1 | 0/0 | 0/0 | 0/0 | 0/0 | ENSBTAT00000011681.5 | c.323_324insTCGCGCATTTGTGCCACGGCGTGAGTGATCTCGGCCAATGCCGCGCCCG | p.Phe109fs |
| KCNS1 | 13 | 74195575 | C>CAACACCCAGATTG | 0/1 | 0/0 | 0/0 | 0/0 | 0/0 | ENSBTAT00000011681.5 | c.319_320insCAATCTGGGTGTT | p.Gly107fs |
| ENSBTAG00000037824 | 14 | 2775036 | T>G | 0/1 | 0/0 | 0/0 | 0/0 | 0/0 | ENSBTAT00000006034.5 | c.1053T>G | p.Tyr351* |
| ENSBTAG00000026340 | 14 | 2931653 | CCCTA>C | 0/1 | 0/0 | 0/0 | 0/0 | 0/0 | ENSBTAT00000037390.4 | c.1818-5_1818-2delCCTA |  |
| ENSBTAG00000026340 | 14 | 2931659 | G>GGTA | 0/1 | 0/0 | 0/0 | 0/0 | 0/0 | ENSBTAT00000037390.4 | c.1818_1819insGTA | p.Arg606_Thr607insVal |
| ENSBTAG00000026340 | 14 | 2931661 | CATG>C | 0/1 | 0/0 | 0/0 | 0/0 | 0/0 | ENSBTAT00000037390.4 | c.1821_1823delATG | p.Cys608del |
| ENSBTAG00000026340 | 14 | 2931670 | A>G | 0/1 | 0/0 | 0/0 | 0/0 | 0/0 | ENSBTAT00000037390.4 | c.1829A>G | p.Glu610Gly |
| ENSBTAG00000026340 | 14 | 2931671 | G>GTAATTCT | 0/1 | 0/0 | 0/0 | 0/0 | 0/0 | ENSBTAT00000037390.4 | c.1830_1831insTAATTCT | p.Ala611fs |
| ENSBTAG00000026340 | 14 | 2931676 | T>G | 0/1 | 0/0 | 0/0 | 0/0 | 0/0 | ENSBTAT00000037390.4 | c.1835T>G | p.Leu612Arg |
| ENSBTAG00000026340 | 14 | 2931678 | A>ACGGAAC | 0/1 | 0/0 | 0/0 | 0/0 | 0/0 | ENSBTAT00000037390.4 | c.1837_1838insCGGAAC | p.Lys613delinsThrGluGln |
| ENSBTAG00000026340 | 14 | 2931681 | G>GAGAAGACT | 0/1 | 0/0 | 0/0 | 0/0 | 0/0 | ENSBTAT00000037390.4 | c.1840_1841insAGAAGACT | p.Gly614fs |
| TG | 14 | 9495178 | TCA>T | 0/1 | 0/0 | 0/0 | 0/0 | 0/0 | ENSBTAT00000010295.4 | c.903_904delTG | p.Cys301fs |
| ENSBTAG00000032812 | 14 | 45039311 | TCA>T | 0/1 | 0/0 | 0/0 | 0/0 | 0/0 | ENSBTAT00000046579.3 | c.59_60delTG | p.Val20fs |
| CSMD3 | 14 | 54290721 | A>AGGCCATGCGTACGATCCTCACTGGCATGACAGCTATTTAGTCGGCGTGACCCAATACGGATTGCAGCAGTTCAGCTGTCGTCGTCAGAAACATCAGAGCCTGCCGGGGAAAGTCTTT | 0/1 | 0/0 | 0/0 | 0/0 | 0/0 | ENSBTAT00000027766.5 | c.4726_4727insGGCCATGCGTACGATCCTCACTGGCATGACAGCTATTTAGTCGGCGTGACCCAATACGGATTGCAGCAGTTCAGCTGTCGTCGTCAGAAACATCAGAGCCTGCCGGGGAAAGTCTTT | p.Asn1576delinsArgProCysValArgSerSerLeuAlaTerGlnLeuPheSerArgArgAspProIleArgIleAlaAlaValGlnLeuSerSerSerGluThrSerGluProAlaGlyGluSerLeuTyr |
| RIMS2 | 14 | 62807396 | A>ACGACGGGCGG | 0/1 | 0/0 | 0/0 | 0/0 | 0/0 | ENSBTAT00000060962.2 | c.1868_1869insCCGCCCGTCG | p.Glu625fs |
| **Gene** | **BTA** | **Position** | **Base change** | **Genotype** | | | | | **Transcript** | **cDNA** | **Protein** |
|  |  |  |  | **Case** | **Sire** | **Dam** | **a** | **b** |  |  |  |
| RIMS2 | 14 | 62807398 | A>AAAGACGCCGTCGCAGGTATCGGCGAGGCCATCGAGGTGAAAACCACCGGTCAGCAGCGCCAGCGCTAAAATGCAAAACAG | 0/1 | 0/0 | 0/0 | 0/0 | 0/0 | ENSBTAT00000060962.2 | c.1866_1867insCTGTTTTGCATTTTAGCGCTGGCGCTGCTGACCGGTGGTTTTCACCTCGATGGCCTCGCCGATACCTGCGACGGCGTCTT | p.Phe623fs |
| ENSBTAG00000048317 | 14 | 77387192 | TGAA>T | 0/1 | 0/0 | 0/0 | 0/0 | 0/0 | ENSBTAT00000065964.1 | c.1476_1478delAGA | p.Glu493del |
| BIRC3 | 15 | 6653394 | AG>A | 0/1 | 0/0 | 0/0 | 0/0 | 0/0 | ENSBTAT00000034719.4 | c.621delC | p.Cys208fs |
| BIRC3 | 15 | 6653396 | G>T | 0/1 | 0/0 | 0/0 | 0/0 | 0/0 | ENSBTAT00000034719.4 | c.620C>A | p.Ala207Asp |
| RAB39A | 15 | 17798258 | G>GT | 0/1 | 0/0 | 0/0 | 0/0 | 0/0 | ENSBTAT00000024288.4 | c.261dupT | p.Gly88fs |
| RAB39A | 15 | 17798262 | G>GGTGCGGATTATGGTTATACCAATGGTTACCGCGATGCGAATGGCGTAAACAGCAATGTTGGCAGCGCCAGCCTGCAATTAACGCAGACCCTTTTTGATATGTCAAAA | 0/1 | 0/0 | 0/0 | 0/0 | 0/0 | ENSBTAT00000024288.4 | c.263_264insGTGCGGATTATGGTTATACCAATGGTTACCGCGATGCGAATGGCGTAAACAGCAATGTTGGCAGCGCCAGCCTGCAATTAACGCAGACCCTTTTTGATATGTCAAAA | p.Gly89fs |
| ACAT1 | 15 | 18021465 | A>G | 0/1 | 0/0 | 0/0 | 0/0 | 0/0 | ENSBTAT00000017122.4 | c.427A>G | p.Met143Val |
| ZW10 | 15 | 24644700 | GA>G | 0/1 | 0/0 | 0/0 | 0/0 | 0/0 | ENSBTAT00000026733.5 | c.397delT | p.Ser133fs |
| APOA5 | 15 | 27872279 | G>C | 0/1 | 0/0 | 0/0 | 0/0 | 0/0 | ENSBTAT00000026336.4 | c.699C>G | p.Ser233Arg |
| APOA5 | 15 | 27872281 | TGGAGAG>T | 0/1 | 0/0 | 0/0 | 0/0 | 0/0 | ENSBTAT00000026336.4 | c.691_696delCTCTCC | p.Leu231_Ser232del |
| C11orf63 | 15 | 34121567 | G>C | 0/1 | 0/0 | 0/0 | 0/0 | 0/0 | ENSBTAT00000054834.2 | c.2245G>C | p.Asp749His |
| ABCC8 | 15 | 35580891 | T>G | 0/1 | 0/0 | 0/0 | 0/0 | 0/0 | ENSBTAT00000046676.3 | c.822+2T>G |  |
| WEE1 | 15 | 43670885 | G>GGCCAAGAGCGGAGGCGGGTCTGAGTGAAGCGAAAGCGGGCATTGCAGCAATTCCTAAGATTTAACGGTTGTGGCGCAAACGCGGATTGGCAACCAGGGCCAT | 0/1 | 0/0 | 0/0 | 0/0 | 0/0 | ENSBTAT00000006114.4 | c.1528_1529insATGGCCCTGGTTGCCAATCCGCGTTTGCGCCACAACCGTTAAATCTTAGGAATTGCTGCAATGCCCGCTTTCGCTTCACTCAGACCCGCCTCCGCTCTTGGC | p.Ala510delinsAspGlyProGlyCysGlnSerAlaPheAlaProGlnProLeuAsnLeuArgAsnCysCysAsnAlaArgPheArgPheThrGlnThrArgLeuArgSerTrpPro |
| WEE1 | 15 | 43670890 | TA>T | 0/1 | 0/0 | 0/0 | 0/0 | 0/0 | ENSBTAT00000006114.4 | c.1523delT | p.Val508fs |
| DNHD1 | 15 | 47147515 | TCA>T | 0/1 | 0/0 | 0/0 | 0/0 | 0/0 | ENSBTAT00000019733.5 | c.2320+2_2320+3delTG |  |
| ENSBTAG00000032259 | 15 | 48046108 | T>G | 0/1 | 0/0 | 0/0 | 0/0 | 0/0 | ENSBTAT00000025810.1 | c.131T>G | p.Val44Gly |
| MADD | 15 | 78347836 | CG>C | 0/1 | 0/0 | 0/0 | 0/0 | 0/0 | ENSBTAT00000028927.5 | c.789delG | p.Gln264fs |
| MADD | 15 | 78347839 | G>T | 0/1 | 0/0 | 0/0 | 0/0 | 0/0 | ENSBTAT00000028927.5 | c.788G>T | p.Gly263Val |
| MADD | 15 | 78377083 | A>AAAAAT | 0/1 | 0/0 | 0/0 | 0/0 | 0/0 | ENSBTAT00000028927.5 | c.4406_4407insAAATA | p.Asn1469fs |
| **Gene** | **BTA** | **Position** | **Base change** | **Genotype** | | | | | **Transcript** | **cDNA** | **Protein** |
|  |  |  |  | **Case** | **Sire** | **Dam** | **a** | **b** |  |  |  |
| MADD | 15 | 78377085 | C>CGCTGGATGCGGTACTCAGTTATCTGGCGCTGCTGCAAACCGGCGCGCGTCTGCTGCCGCTTAATCCGCAACTGCCGCAGGCGTTATGCGAAGCATTACTGCCGCAGCTCGAT | 0/1 | 0/0 | 0/0 | 0/0 | 0/0 | ENSBTAT00000028927.5 | c.4407_4408insGCTGGATGCGGTACTCAGTTATCTGGCGCTGCTGCAAACCGGCGCGCGTCTGCTGCCGCTTAATCCGCAACTGCCGCAGGCGTTATGCGAAGCATTACTGCCGCAGCTCGAT | p.Ile1470fs |
| ENSBTAG00000039348 | 15 | 80686694 | G>T | 0/1 | 0/0 | ./. | 0/0 | 0/0 | ENSBTAT00000055245.2 | c.827G>T | p.Ser276Ile |
| ENSBTAG00000039348 | 15 | 80686700 | C>G | 0/1 | 0/0 | ./. | 0/0 | 0/0 | ENSBTAT00000055245.2 | c.833C>G | p.Ala278Gly |
| ENSBTAG00000039348 | 15 | 80686702 | A>T | 0/1 | 0/0 | ./. | 0/0 | 0/0 | ENSBTAT00000055245.2 | c.835A>T | p.Thr279Ser |
| ENSBTAG00000039348 | 15 | 80686721 | T>C | 0/1 | 0/0 | ./. | 0/0 | 0/0 | ENSBTAT00000055245.2 | c.854T>C | p.Val285Ala |
| ENSBTAG00000039348 | 15 | 80686729 | A>T | 0/1 | 0/0 | 0/0 | 0/0 | 0/0 | ENSBTAT00000055245.2 | c.862A>T | p.Ile288Phe |
| ENSBTAG00000039348 | 15 | 80686736 | G>C | 0/1 | 0/0 | 0/0 | 0/0 | 0/0 | ENSBTAT00000055245.2 | c.869G>C | p.Ser290Thr |
| ENSBTAG00000039348 | 15 | 80686739 | T>C | 0/1 | 0/0 | 0/0 | 0/0 | 0/0 | ENSBTAT00000055245.2 | c.872T>C | p.Ile291Thr |
| OR5J2 | 15 | 80828040 | TGG>T | 0/1 | 0/0 | 0/0 | 0/0 | 0/0 | ENSBTAT00000064409.1 | c.854_855delCC | p.Pro285fs |
| DTX4 | 15 | 83663013 | CAG>C | 0/1 | 0/0 | 0/0 | 0/0 | 0/0 | ENSBTAT00000005289.5 | c.1523_1524delAG | p.Glu508fs |
| OR4D6 | 15 | 83995153 | A>AGGTTGAACGGCAGCATCAGCTGTTCGCGGTACTGCTGCGAGAAATCGAGCCAGCCGCGAGCAAAACCATTGGTGGTATCCGGTGAAAAGGGAGGAAAGATAAAT | 0/1 | 0/0 | 0/0 | 0/0 | 0/0 | ENSBTAT00000039492.4 | c.847_848insGGTTGAACGGCAGCATCAGCTGTTCGCGGTACTGCTGCGAGAAATCGAGCCAGCCGCGAGCAAAACCATTGGTGGTATCCGGTGAAAAGGGAGGAAAGATAAAT | p.Asn283fs |
| PPP1R15B | 16 | 1971448 | C>CAGAAGGTGCGCCGCCGAGCTGCCCGACCGCCACCTGACTGTTTTGCGCTTTGATCGCGGTGACCACATCGG | 0/1 | 0/0 | 0/0 | 0/0 | 0/0 | ENSBTAT00000017382.4 | c.714_715insCCGATGTGGTCACCGCGATCAAAGCGCAAAACAGTCAGGTGGCGGTCGGGCAGCTCGGCGGCGCACCTTCT | p.Val239fs |
| CD55 | 16 | 5120185 | T>C | 0/1 | 0/0 | 0/0 | 0/0 | 0/0 | ENSBTAT00000009178.5 | c.479A>G | p.Glu160Gly |
| CD55 | 16 | 5120193 | CT>C | 0/1 | 0/0 | 0/0 | 0/0 | 0/0 | ENSBTAT00000009178.5 | c.470delA | p.Lys157fs |
| AHCTF1 | 16 | 31198284 | AG>A | 0/1 | 0/0 | 0/0 | 0/0 | 0/0 | ENSBTAT00000018538.5 | c.1099delG | p.Glu367fs |
| AHCTF1 | 16 | 31198286 | G>T | 0/1 | 0/0 | 0/0 | 0/0 | 0/0 | ENSBTAT00000018538.5 | c.1098G>T | p.Arg366Ser |
| CEP170 | 16 | 34728926 | TC>T | 0/1 | 0/0 | 0/0 | 0/0 | 0/0 | ENSBTAT00000009955.5 | c.602delC | p.Pro201fs |
| F5 | 16 | 37987143 | TC>T | 0/1 | 0/0 | 0/0 | 0/0 | 0/0 | ENSBTAT00000023573.5 | c.4993delG | p.Glu1665fs |
| F5 | 16 | 37987146 | T>A | 0/1 | 0/0 | 0/0 | 0/0 | 0/0 | ENSBTAT00000023573.5 | c.4991A>T | p.Lys1664Met |
| UBE2J2 | 16 | 52449313 | C>A | 0/1 | 0/0 | 0/0 | 0/0 | 0/0 | ENSBTAT00000021109.4 | c.669C>A | p.Asn223Lys |
| UBE2J2 | 16 | 52449319 | C>G | 0/1 | 0/0 | 0/0 | 0/0 | 0/0 | ENSBTAT00000021109.4 | c.675C>G | p.His225Gln |
| **Gene** | **BTA** | **Position** | **Base change** | **Genotype** | | | | | **Transcript** | **cDNA** | **Protein** |
|  |  |  |  | **Case** | **Sire** | **Dam** | **a** | **b** |  |  |  |
| UBE2J2 | 16 | 52449321 | A>ACCGTCATACCGCGAATCTCATCGAGAATGAGTTCATAAATGCGCTCGTCGCGACTGCCGGGT | 0/1 | 0/0 | 0/0 | 0/0 | 0/0 | ENSBTAT00000021109.4 | c.677_678insCCGTCATACCGCGAATCTCATCGAGAATGAGTTCATAAATGCGCTCGTCGCGACTGCCGGGT | p.Gly227fs |
| ZBTB17 | 16 | 52876870 | GA>G | 0/1 | 0/0 | 0/0 | 0/0 | 0/0 | ENSBTAT00000044658.3 | c.419delA | p.Glu140fs |
| FHAD1 | 16 | 53419877 | AC>A | 0/1 | 0/0 | 0/0 | 0/0 | 0/0 | ENSBTAT00000003121.4 | c.1024delG | p.Val342fs |
| FHAD1 | 16 | 53419879 | C>A | 0/1 | 0/0 | 0/0 | 0/0 | 0/0 | ENSBTAT00000003121.4 | c.1023G>T | p.Glu341Asp |
| XPR1 | 16 | 63449175 | T>TCCAGCA | 0/1 | 0/0 | 0/0 | 0/0 | 0/0 | ENSBTAT00000012084.5 | c.699_700insCCAGCA | p.Thr233_Thr234insProAla |
| XPR1 | 16 | 63449177 | C>CGCATCCAGATGAAACGGCACGCCGCCCCAATGGTGGGCGGTACGCATCACCAGCGCGG | 0/1 | 0/0 | 0/0 | 0/0 | 0/0 | ENSBTAT00000012084.5 | c.701_702insGCATCCAGATGAAACGGCACGCCGCCCCAATGGTGGGCGGTACGCATCACCAGCGCGG | p.Phe235fs |
| ELF3 | 16 | 70828106 | TCCA>T | 0/1 | 0/0 | 0/0 | 0/0 | 0/0 | ENSBTAT00000011536.5 | c.597_599delCAC | p.Thr200del |
| GPR37L1 | 16 | 70887115 | AC>A | 0/1 | 0/0 | 0/0 | 0/0 | 0/0 | ENSBTAT00000019957.3 | c.238delC | p.Gln80fs |
| LPGAT1 | 16 | 73577461 | GT>G | 0/1 | 0/0 | 0/0 | 0/0 | 0/0 | ENSBTAT00000006773.4 | c.651+2delT |  |
| ASPM | 16 | 77947077 | A>AGT | 0/1 | 0/0 | 0/0 | 0/0 | 0/0 | ENSBTAT00000010340.5 | c.4929_4930insAC | p.Cys1644fs |
| ASPM | 16 | 77947080 | C>CATGCAGG | 0/1 | 0/0 | 0/0 | 0/0 | 0/0 | ENSBTAT00000010340.5 | c.4926_4927insCCTGCAT | p.Ala1643fs |
| ASPM | 16 | 77947084 | C>A | 0/1 | 0/0 | 0/0 | 0/0 | 0/0 | ENSBTAT00000010340.5 | c.4923G>T | p.Gln1641His |
| ZNF281 | 16 | 81040764 | TG>T | 0/1 | 0/0 | 0/0 | 0/0 | 0/0 | ENSBTAT00000065279.1 | c.1378delC | p.Gln460fs |
| RBM46 | 17 | 2565208 | G>GTACTGGCGGTGCGTGCGCTGGATGCCCTGCTGGATTACCAGGACTACCCGATTCTGGCGGCTAAACGCGGCGCGATGGGCCGTCGTACGCTGGGCATTGGCGTAAT | 0/1 | 0/0 | 0/0 | 0/0 | 0/0 | ENSBTAT00000002223.5 | c.1038_1039insATTACGCCAATGCCCAGCGTACGACGGCCCATCGCGCCGCGTTTAGCCGCCAGAATCGGGTAGTCCTGGTAATCCAGCAGGGCATCCAGCGCACGCACCGCCAGTA | p.Pro347fs |
| DCHS2 | 17 | 3189446 | AGC>A | 0/1 | 0/0 | 0/0 | 0/0 | 0/0 | ENSBTAT00000017605.5 | c.2172_2173delGC | p.His725fs |
| DCHS2 | 17 | 3316726 | C>CGGACTGCGCCTGCGTTGTGATCGGCGGCAGTATCGGTCTTGCAGGCGGCTACCTGCCGCGGGTTGAGAAGTACTTGTCACAAGAACCTG | 0/1 | 0/0 | 0/0 | 0/0 | 0/0 | ENSBTAT00000017605.5 | c.5996_5997insGGACTGCGCCTGCGTTGTGATCGGCGGCAGTATCGGTCTTGCAGGCGGCTACCTGCCGCGGGTTGAGAAGTACTTGTCACAAGAACCTG | p.Leu2000fs |
| ARFIP1 | 17 | 4788143 | TC>T | 0/1 | 0/0 | 0/0 | 0/0 | 0/0 | ENSBTAT00000029827.4 | c.367delG | p.Glu123fs |
| ABCE1 | 17 | 13344991 | C>T | 0/1 | 0/0 | 0/0 | 0/0 | 0/0 | ENSBTAT00000048747.3 | c.874G>A | p.Val292Ile |
| INPP4B | 17 | 15863875 | G>GTCGTCTACCAGGATCATA | 0/1 | 0/0 | 0/0 | 0/0 | 0/0 | ENSBTAT00000018761.4 | c.25_26insTCTACCAGGATCATATCG | p.Val8_Gly9insValTyrGlnAspHisIle |
| **Gene** | **BTA** | **Position** | **Base change** | **Genotype** | | | | | **Transcript** | **cDNA** | **Protein** |
|  |  |  |  | **Case** | **Sire** | **Dam** | **a** | **b** |  |  |  |
| INPP4B | 17 | 15863880 | C>CAGATTCTTTGGTGACCTCCGTCCCTTTTATCCCCATCGCAATACCGACGTTAGCCTGCTTCAGCGCGGGAGCGTCGTTTACGCCGTCGCCGGTCATCCCG | 0/1 | 0/0 | 0/0 | 0/0 | 0/0 | ENSBTAT00000018761.4 | c.28_29insGATTCTTTGGTGACCTCCGTCCCTTTTATCCCCATCGCAATACCGACGTTAGCCTGCTTCAGCGCGGGAGCGTCGTTTACGCCGTCGCCGGTCATCCCGA | p.Thr10fs |
| FZD10 | 17 | 47936032 | T>C | 0/1 | 0/0 | 0/0 | 0/0 | 0/0 | ENSBTAT00000009267.4 | c.1352A>G | p.Tyr451Cys |
| KSR2 | 17 | 59844919 | G>GATATTCGGGTTCGCGCCGTGGCCGCTGTAGCCGGTCACCGCAATCACCAGCCCGACAAACGCGGCGCTGGAACCGAGATAACTGGGCACACGCCCGCCAACAACAAGGAAGAA | 0/1 | 0/0 | 0/0 | 0/0 | 0/0 | ENSBTAT00000061442.2 | c.1626_1627insATATTCGGGTTCGCGCCGTGGCCGCTGTAGCCGGTCACCGCAATCACCAGCCCGACAAACGCGGCGCTGGAACCGAGATAACTGGGCACACGCCCGCCAACAACAAGGAAGAA | p.Pro543fs |
| CFAP73 | 17 | 63445860 | TC>T | 0/1 | 0/0 | 0/0 | 0/0 | 0/0 | ENSBTAT00000029529.4 | c.840delC | p.Glu281fs |
| CFAP73 | 17 | 63445862 | G>T | 0/1 | 0/0 | 0/0 | 0/0 | 0/0 | ENSBTAT00000029529.4 | c.841G>T | p.Glu281* |
| SGSM1 | 17 | 67297397 | G>GCCTTATGAACAGGAGCTGGAACGGGTACAGACACAGATCGATCACTTCCGCCTTATGCTGGAAGGCCGGGCCGCCAGCGCCATTGCGC | 0/1 | 0/0 | 0/0 | 0/0 | 0/0 | ENSBTAT00000008658.5 | c.750_751insCCTTATGAACAGGAGCTGGAACGGGTACAGACACAGATCGATCACTTCCGCCTTATGCTGGAAGGCCGGGCCGCCAGCGCCATTGCGC | p.Asn251fs |
| KIAA1671 | 17 | 67415424 | T>TGCTCAA | 0/1 | 0/0 | 0/0 | 0/0 | 0/0 | ENSBTAT00000061095.2 | c.3579_3580insCTCAAG | p.Met1193_Ala1194insLeuLys |
| KIAA1671 | 17 | 67415428 | C>CGGGCT | 0/1 | 0/0 | 0/0 | 0/0 | 0/0 | ENSBTAT00000061095.2 | c.3583_3584insGGCTG | p.Glu1195fs |
| KIAA1671 | 17 | 67415430 | A>AAAAACTGATGC | 0/1 | 0/0 | 0/0 | 0/0 | 0/0 | ENSBTAT00000061095.2 | c.3584_3585insAAAACTGATGC | p.Tyr1196fs |
| KIAA1671 | 17 | 67415437 | G>GC | 0/1 | 0/0 | 0/0 | 0/0 | 0/0 | ENSBTAT00000061095.2 | c.3591_3592insC | p.Arg1198fs |
| KIAA1671 | 17 | 67415438 | A>ACTCGCTCAAGCGCAAAAAGAT | 0/1 | 0/0 | 0/0 | 0/0 | 0/0 | ENSBTAT00000061095.2 | c.3592_3593insCTCGCTCAAGCGCAAAAAGAT | p.Arg1198delinsThrArgSerSerAlaLysArgTrp |
| PRR14L | 17 | 72482697 | TA>T | 0/1 | 0/0 | 0/0 | 0/0 | 0/0 | ENSBTAT00000011858.4 | c.3353delT | p.Val1118fs |
| ZNF280B | 17 | 73002885 | C>G | 0/1 | 0/0 | 0/0 | 0/0 | 0/0 | ENSBTAT00000001332.4 | c.1159G>C | p.Asp387His |
| SMPD4 | 17 | 74409242 | C>CG | 0/1 | 0/0 | 0/0 | 0/0 | 0/0 | ENSBTAT00000029373.4 | c.1149_1150insG | p.Leu384fs |
| SMPD4 | 17 | 74409243 | C>CGGCGACCGCGTGTTCGAGAATCGTTTTGTTACCGATTGAGAGATATTGTTTCGGGCATTCCGTTTGCA | 0/1 | 0/0 | 0/0 | 0/0 | 0/0 | ENSBTAT00000029373.4 | c.1150_1151insGGCGACCGCGTGTTCGAGAATCGTTTTGTTACCGATTGAGAGATATTGTTTCGGGCATTCCGTTTGCA | p.Leu384fs |
| CDC45 | 17 | 74744009 | CCCT>C | 0/1 | 0/0 | 0/0 | 0/0 | 0/0 | ENSBTAT00000005623.5 | c.1469_1471delCCT | p.Pro490del |
| CDC45 | 17 | 74744014 | G>GCCCGCGGC | 0/1 | 0/0 | 0/0 | 0/0 | 0/0 | ENSBTAT00000005623.5 | c.1473_1474insCCCGCGGC | p.Val492fs |
| CDC45 | 17 | 74744018 | A>AAAC | 0/1 | 0/0 | 0/0 | 0/0 | 0/0 | ENSBTAT00000005623.5 | c.1477_1478insAAC | p.Met493delinsLysLeu |
| **Gene** | **BTA** | **Position** | **Base change** | **Genotype** | | | | | **Transcript** | **cDNA** | **Protein** |
|  |  |  |  | **Case** | **Sire** | **Dam** | **a** | **b** |  |  |  |
| CDC45 | 17 | 74744019 | T>TGTC | 0/1 | 0/0 | 0/0 | 0/0 | 0/0 | ENSBTAT00000005623.5 | c.1479_1480insTCG | p.Met493_Ala494insSer |
| CDC45 | 17 | 74744022 | C>G | 0/1 | 0/0 | 0/0 | 0/0 | 0/0 | ENSBTAT00000005623.5 | c.1481C>G | p.Ala494Gly |
| CDC45 | 17 | 74744024 | G>GA | 0/1 | 0/0 | 0/0 | 0/0 | 0/0 | ENSBTAT00000005623.5 | c.1483_1484insA | p.Ala495fs |
| CDC45 | 17 | 74744025 | C>CA | 0/1 | 0/0 | 0/0 | 0/0 | 0/0 | ENSBTAT00000005623.5 | c.1484_1485insA | p.Pro496fs |
| CDC45 | 17 | 74744027 | C>CG | 0/1 | 0/0 | 0/0 | 0/0 | 0/0 | ENSBTAT00000005623.5 | c.1486_1487insG | p.Pro496fs |
| CDC45 | 17 | 74744028 | C>A | 0/1 | 0/0 | 0/0 | 0/0 | 0/0 | ENSBTAT00000005623.5 | c.1487C>A | p.Pro496His |
| CDC45 | 17 | 74744032 | G>GTTCGCGCGTAACGCGAA | 0/1 | 0/0 | 0/0 | 0/0 | 0/0 | ENSBTAT00000005623.5 | c.1491_1492insTTCGCGCGTAACGCGAA | p.Ser498fs |
| CDC45 | 17 | 74744037 | C>CCTATCGT | 0/1 | 0/0 | 0/0 | 0/0 | 0/0 | ENSBTAT00000005623.5 | c.1496_1497insCTATCGT | p.Glu500fs |
| CDC45 | 17 | 74744039 | G>GCA | 0/1 | 0/0 | 0/0 | 0/0 | 0/0 | ENSBTAT00000005623.5 | c.1498_1499insCA | p.Glu500fs |
| RFWD3 | 18 | 2117173 | C>CGGCAGGCCGCCCGCA | 0/1 | 0/0 | 0/0 | 0/0 | 0/0 | ENSBTAT00000005847.4 | c.2150_2151insTGCGGGCGGCCTGCC | p.Glu717delinsAspAlaGlyGlyLeuPro |
| RFWD3 | 18 | 2117177 | G>GCGACC | 0/1 | 0/0 | 0/0 | 0/0 | 0/0 | ENSBTAT00000005847.4 | c.2146_2147insGGTCG | p.Pro716fs |
| RFWD3 | 18 | 2117179 | ATT>A | 0/1 | 0/0 | 0/0 | 0/0 | 0/0 | ENSBTAT00000005847.4 | c.2143_2144delAA | p.Asn715fs |
| RFWD3 | 18 | 2117184 | GGAA>G | 0/1 | 0/0 | 0/0 | 0/0 | 0/0 | ENSBTAT00000005847.4 | c.2137_2139delTTC | p.Phe713del |
| RFWD3 | 18 | 2117190 | T>TC | 0/1 | 0/0 | 0/0 | 0/0 | 0/0 | ENSBTAT00000005847.4 | c.2133_2134insG | p.Ile712fs |
| RFWD3 | 18 | 2117193 | CATTCTTG>C | 0/1 | 0/0 | 0/0 | 0/0 | 0/0 | ENSBTAT00000005847.4 | c.2124_2130delCAAGAAT | p.Lys709fs |
| RFWD3 | 18 | 2117202 | T>A | 0/1 | 0/0 | 0/0 | 0/0 | 0/0 | ENSBTAT00000005847.4 | c.2122A>T | p.Thr708Ser |
| RFWD3 | 18 | 2117206 | CAATTTGCA>C | 0/1 | 0/0 | 0/0 | 0/0 | 0/0 | ENSBTAT00000005847.4 | c.2110_2117delTGCAAATT | p.Cys704fs |
| RFWD3 | 18 | 2117217 | A>C | 0/1 | 0/0 | 0/0 | 0/0 | 0/0 | ENSBTAT00000005847.4 | c.2107T>G | p.Ser703Ala |
| RFWD3 | 18 | 2117219 | G>GGAC | 0/1 | 0/0 | 0/0 | 0/0 | 0/0 | ENSBTAT00000005847.4 | c.2104_2105insGTC | p.Gly701_Pro702insArg |
| RFWD3 | 18 | 2117222 | C>A | 0/1 | 0/0 | 0/0 | 0/0 | 0/0 | ENSBTAT00000005847.4 | c.2102G>T | p.Gly701Val |
| ENSBTAG00000009805 | 18 | 2781758 | TGC>T | 0/1 | 0/0 | 0/0 | 0/0 | 0/0 | ENSBTAT00000012929.4 | c.572_573delGC | p.Arg191fs |
| GAS8 | 18 | 14836496 | T>TTTTGCCGATCCTTTCCCCTGAGCTAACGAC | 0/1 | 0/0 | 0/0 | 0/0 | 0/0 | ENSBTAT00000009333.4 | c.92_93insTTTGCCGATCCTTTCCCCTGAGCTAACGAC | p.Val31_Glu32insLeuProIleLeuSerProGluLeuThrThr |
| SIAH1 | 18 | 16838496 | T>A | 0/1 | 0/0 | 0/0 | 0/0 | 0/0 | ENSBTAT00000053788.2 | c.260A>T | p.Gln87Leu |
| SIAH1 | 18 | 16838502 | T>A | 0/1 | 0/0 | 0/0 | 0/0 | 0/0 | ENSBTAT00000053788.2 | c.254A>T | p.Gln85Leu |
| MMP2 | 18 | 23837645 | G>A | 0/1 | 0/0 | 0/0 | 0/0 | 0/0 | ENSBTAT00000025657.2 | c.865G>A | p.Gly289Arg |
| CIAPIN1 | 18 | 25514109 | C>CACTTCGATTTCAACCGGCTTGCCAGGGGCAA | 0/1 | 0/0 | 0/0 | 0/0 | 0/0 | ENSBTAT00000002418.5 | c.721_722insTTGCCCCTGGCAAGCCGGTTGAAATCGAAGT | p.Arg241fs |
| **Gene** | **BTA** | **Position** | **Base change** | **Genotype** | | | | | **Transcript** | **cDNA** | **Protein** |
|  |  |  |  | **Case** | **Sire** | **Dam** | **a** | **b** |  |  |  |
| CIAPIN1 | 18 | 25514112 | T>TTGTGCGCGGCACTGATGGCTTGCGCGATACCGCCGCAGGCAGCGATATGG | 0/1 | 0/0 | 0/0 | 0/0 | 0/0 | ENSBTAT00000002418.5 | c.718_719insCCATATCGCTGCCTGCGGCGGTATCGCGCAAGCCATCAGTGCCGCGCACA | p.Lys240fs |
| COG8 | 18 | 36600730 | GC>G | 0/1 | 0/0 | 0/0 | 0/0 | 0/0 | ENSBTAT00000002179.5 | c.1821delG | p.Gln608fs |
| COG8 | 18 | 36600735 | G>A | 0/1 | 0/0 | 0/0 | 0/0 | 0/0 | ENSBTAT00000002179.5 | c.1817C>T | p.Ala606Val |
| HAUS5 | 18 | 46530254 | C>CGTGCTGTTACTCGAAGCCGGTGGCCCGGATTACCGTCTGGATTTCCGCACACAAATGCCTGCGGCGCTGGCGT | 0/1 | 0/0 | 0/0 | 0/0 | 0/0 | ENSBTAT00000017877.5 | c.427_428insGTGCTGTTACTCGAAGCCGGTGGCCCGGATTACCGTCTGGATTTCCGCACACAAATGCCTGCGGCGCTGGCGT | p.Leu143fs |
| SUPT5H | 18 | 49421663 | T>C | 0/1 | 0/0 | 0/0 | 0/0 | 0/0 | ENSBTAT00000044229.3 | c.1069T>C | p.Phe357Leu |
| SPTBN4 | 18 | 50112027 | C>CATGTGGAGTTCAATACTTTGACGGTGGTGAATGCGGAAAACGTCAAATGGCCGTTGGCAATCTACCGCTTTCTTAAGCAGATCGGCAGTCGCTATAT | 0/1 | 0/0 | 0/0 | 0/0 | 0/0 | ENSBTAT00000007434.5 | c.3718_3719insATGTGGAGTTCAATACTTTGACGGTGGTGAATGCGGAAAACGTCAAATGGCCGTTGGCAATCTACCGCTTTCTTAAGCAGATCGGCAGTCGCTATAT | p.Arg1240fs |
| CIC | 18 | 51335364 | A>AGTTTTGCTTCTGAGATTTTAGCGATGTCTTCG | 0/1 | 0/0 | 0/0 | 0/0 | 0/0 | ENSBTAT00000064364.1 | c.661_662insCGAAGACATCGCTAAAATCTCAGAAGCAAAAC | p.Val221fs |
| CIC | 18 | 51335366 | C>CGTCG | 0/1 | 0/0 | 0/0 | 0/0 | 0/0 | ENSBTAT00000064364.1 | c.659_660insCGAC | p.Val221fs |
| CIC | 18 | 51335367 | A>AT | 0/1 | 0/0 | 0/0 | 0/0 | 0/0 | ENSBTAT00000064364.1 | c.658_659insA | p.Val220fs |
| CIC | 18 | 51335370 | G>GCACTTCGCCGCGGGTGGGCTCTATCAGACGATTGAGAAGGCGTACCATGGTGGACTTACCGGAA | 0/1 | 0/0 | 0/0 | 0/0 | 0/0 | ENSBTAT00000064364.1 | c.655_656insTTCCGGTAAGTCCACCATGGTACGCCTTCTCAATCGTCTGATAGAGCCCACCCGCGGCGAAGTG | p.Ala219fs |
| NKPD1 | 18 | 53211742 | CTG>C | 0/1 | 0/0 | 0/0 | 0/0 | 0/0 | ENSBTAT00000053125.2 | c.2227_2228delCA | p.Gln743fs |
| ENSBTAG00000011119 | 18 | 55410242 | G>A | 0/1 | 0/0 | 0/0 | 0/0 | 0/0 | ENSBTAT00000014771.5 | c.251C>T | p.Thr84Ile |
| PPP1R15A | 18 | 55928321 | TC>T | 0/1 | 0/0 | 0/0 | 0/0 | 0/0 | ENSBTAT00000001702.4 | c.1525delC | p.Arg509fs |
| PPP1R15A | 18 | 55928324 | G>A | 0/1 | 0/0 | 0/0 | 0/0 | 0/0 | ENSBTAT00000001702.4 | c.1526G>A | p.Arg509Gln |
| POLD1 | 18 | 57022206 | G>GGAGCAGCT | 0/1 | 0/0 | 0/0 | 0/0 | 0/0 | ENSBTAT00000014714.5 | c.1665_1666insGAGCAGCT | p.Ser556fs |
| POLD1 | 18 | 57022208 | C>CT | 0/1 | 0/0 | 0/0 | 0/0 | 0/0 | ENSBTAT00000014714.5 | c.1667_1668insT | p.Gln557fs |
| POLD1 | 18 | 57022213 | C>CGTTTCAT | 0/1 | 0/0 | 0/0 | 0/0 | 0/0 | ENSBTAT00000014714.5 | c.1672_1673insGTTTCAT | p.Leu558fs |
| POLD1 | 18 | 57022218 | G>GAT | 0/1 | 0/0 | 0/0 | 0/0 | 0/0 | ENSBTAT00000014714.5 | c.1677_1678insAT | p.Arg560fs |
| POLD1 | 18 | 57022219 | C>CT | 0/1 | 0/0 | 0/0 | 0/0 | 0/0 | ENSBTAT00000014714.5 | c.1678_1679insT | p.Arg560fs |
| KLK11 | 18 | 57458045 | C>A | 0/1 | 0/0 | 0/0 | 0/0 | 0/0 | ENSBTAT00000002912.5 | c.181G>T | p.Ala61Ser |
| ENSBTAG00000045985 | 18 | 60233205 | A>C | 0/1 | 0/0 | 0/0 | 0/0 | 0/0 | ENSBTAT00000054265.2 | c.1324T>G | p.Cys442Gly |
| ENSBTAG00000045985 | 18 | 60233207 | T>TTC | 0/1 | 0/0 | 0/0 | 0/0 | 0/0 | ENSBTAT00000054265.2 | c.1321_1322insGA | p.Lys441fs |
| **Gene** | **BTA** | **Position** | **Base change** | **Genotype** | | | | | **Transcript** | **cDNA** | **Protein** |
|  |  |  |  | **Case** | **Sire** | **Dam** | **a** | **b** |  |  |  |
| PRKCG | 18 | 62046650 | TA>T | 0/1 | 0/0 | 0/0 | 0/0 | 0/0 | ENSBTAT00000018020.4 | c.2094delA | p.Ter698fs |
| TMEM150B | 18 | 62610148 | C>CCTCGACGGCCTGGCGATCATGCCTACCGTGCTGGTGGCGTCAGGCTTTGCGCTGGTGGCCTGGGCAACCGGCAACATGAACTTTGCCAGCTATCTGCATA | 0/1 | 0/0 | 0/0 | 0/0 | 0/0 | ENSBTAT00000055412.1 | c.114_115insCTCGACGGCCTGGCGATCATGCCTACCGTGCTGGTGGCGTCAGGCTTTGCGCTGGTGGCCTGGGCAACCGGCAACATGAACTTTGCCAGCTATCTGCATA | p.Phe39fs |
| ENSBTAG00000048184 | 18 | 62915280 | C>T | 0/1 | 0/0 | 0/0 | 0/0 | 0/0 | ENSBTAT00000065209.1 | c.637+1G>A |  |
| TTYH1 | 18 | 63091046 | C>CT | 0/1 | 0/0 | 0/0 | 0/0 | 0/0 | ENSBTAT00000004515.5 | c.28dupT | p.Ser10fs |
| ENSBTAG00000037830 | 18 | 63215678 | C>A | 0/1 | 0/0 | 0/0 | 0/0 | 0/0 | ENSBTAT00000056991.2 | c.451C>A | p.Leu151Met |
| ENSBTAG00000000930 | 18 | 63226611 | G>T | 0/1 | 0/0 | 0/0 | 0/0 | 0/0 | ENSBTAT00000001231.5 | c.302G>T | p.Ser101Ile |
| SUPT4H1 | 19 | 9545482 | GC>G | 0/1 | 0/0 | 0/0 | 0/0 | 0/0 | ENSBTAT00000004389.3 | c.155delG | p.Cys52fs |
| ACACA | 19 | 13881001 | C>CAGCAGCAGCGAGCCGGTGCCGACGATGCAAAACTGATGCACCGCACACAATGCGCCGAG | 0/1 | 0/0 | 0/0 | 0/0 | 0/0 | ENSBTAT00000023364.5 | c.3515_3516insCTCGGCGCATTGTGTGCGGTGCATCAGTTTTGCATCGTCGGCACCGGCTCGCTGCTGCT | p.Lys1172fs |
| ACACA | 19 | 13881003 | T>TGCGAATAATCCCC | 0/1 | 0/0 | 0/0 | 0/0 | 0/0 | ENSBTAT00000023364.5 | c.3513_3514insGGGGATTATTCGC | p.Lys1172fs |
| CCL1 | 19 | 16111059 | G>GTGGAGCGGGTCCGCACCGAAGCCCGCGCCAGCTTCGCCAGTACCATCAGCGGCACCGACCTGATCGTGGGTGCCCGCTCGGGCTCGGTCAATCTGCTGCTGTATTCGGTGTTCCGCATC | 0/1 | 0/0 | 0/0 | 0/0 | 0/0 | ENSBTAT00000047653.1 | c.43_44insTGGAGCGGGTCCGCACCGAAGCCCGCGCCAGCTTCGCCAGTACCATCAGCGGCACCGACCTGATCGTGGGTGCCCGCTCGGGCTCGGTCAATCTGCTGCTGTATTCGGTGTTCCGCATC | p.Gly15fs |
| IFT20 | 19 | 20375982 | C>CCG | 0/1 | 0/0 | 0/0 | 0/0 | 0/0 | ENSBTAT00000010668.3 | c.96_97insCG | p.Ala33fs |
| IFT20 | 19 | 20375984 | T>TGGCGCCGCGACGGCACCCAGGCGCTGGGCGTAGACCGTTTGATAGCGCTCGCGGTCCGAACCTTCGTCCGGGCGGTCGATATAGGGCGGCAATGGCATATGCCCGACACGGTC | 0/1 | 0/0 | 0/0 | 0/0 | 0/0 | ENSBTAT00000010668.3 | c.94_95insGACCGTGTCGGGCATATGCCATTGCCGCCCTATATCGACCGCCCGGACGAAGGTTCGGACCGCGAGCGCTATCAAACGGTCTACGCCCAGCGCCTGGGTGCCGTCGCGGCGCC | p.Glu32fs |
| INCA1 | 19 | 27051724 | TG>T | 0/1 | 0/0 | 0/0 | 0/0 | 0/0 | ENSBTAT00000011332.5 | c.424delG | p.Ala142fs |
| INCA1 | 19 | 27051728 | G>T | 0/1 | 0/0 | 0/0 | 0/0 | 0/0 | ENSBTAT00000011332.5 | c.424G>T | p.Ala142Ser |
| PELP1 | 19 | 27299064 | T>TTTCA | 0/1 | 0/0 | 0/0 | 0/0 | 0/0 | ENSBTAT00000028337.5 | c.958_959insTTCA | p.Ser320fs |
| PELP1 | 19 | 27299066 | T>TTG | 0/1 | 0/0 | 0/0 | 0/0 | 0/0 | ENSBTAT00000028337.5 | c.960_961insTG | p.Leu321fs |
| **Gene** | **BTA** | **Position** | **Base change** | **Genotype** | | | | | **Transcript** | **cDNA** | **Protein** |
|  |  |  |  | **Case** | **Sire** | **Dam** | **a** | **b** |  |  |  |
| PELP1 | 19 | 27299068 | T>TGAAGTTCTTTTTCCAGCACGCGCGAACGTTCGATCAGGGATCGGATCTTATCCGTAATACTGTTGACATCACCTTTTACCAGATGTGCGACATCTTGCA | 0/1 | 0/0 | 0/0 | 0/0 | 0/0 | ENSBTAT00000028337.5 | c.963_964insAAGTTCTTTTTCCAGCACGCGCGAACGTTCGATCAGGGATCGGATCTTATCCGTAATACTGTTGACATCACCTTTTACCAGATGTGCGACATCTTGCAG | p.Leu321_His322insLysPhePhePheGlnHisAlaArgThrPheAspGlnGlySerAspLeuIleArgAsnThrValAspIleThrPheTyrGlnMetCysAspIleLeuGln |
| C19H17orf49 | 19 | 27438793 | T>TACCGTCAGCGACGAGGGCGCGCGGCTGCTGCCGATGGTCAAGGCGCTGTTGCAG | 0/1 | 0/0 | 0/0 | 0/0 | 0/0 | ENSBTAT00000029238.3 | c.56_57insACCGTCAGCGACGAGGGCGCGCGGCTGCTGCCGATGGTCAAGGCGCTGTTGCAG | p.Phe19delinsLeuProSerAlaThrArgAlaArgGlyCysCysArgTrpSerArgArgCysCysSer |
| ZBTB4 | 19 | 27770121 | C>T | 0/1 | 0/0 | 0/0 | 0/0 | 0/0 | ENSBTAT00000010907.5 | c.2551G>A | p.Gly851Arg |
| ZBTB4 | 19 | 27770383 | AG>A | 0/1 | 0/0 | 0/0 | 0/0 | 0/0 | ENSBTAT00000010907.5 | c.2288delC | p.Pro763fs |
| DNAH2 | 19 | 28103753 | C>A | 0/1 | 0/0 | 0/0 | 0/0 | 0/0 | ENSBTAT00000006277.4 | c.8704C>A | p.Gln2902Lys |
| PFAS | 19 | 28499943 | C>CCGCCG | 0/1 | 0/0 | 0/0 | 0/0 | 0/0 | ENSBTAT00000044654.3 | c.432_433insGCCGC | p.Leu145fs |
| PFAS | 19 | 28499946 | T>TATCACTTTGGCGCCGTGCTCGAAGGCTTGCTGGGCAACGTCAAGAAATATGAGCTGTGGGTGCTCGGTGCCCTGCTCCTGATCGGCCTGTGCCTGTGGGCCCGACGCC | 0/1 | 0/0 | 0/0 | 0/0 | 0/0 | ENSBTAT00000044654.3 | c.434_435insATCACTTTGGCGCCGTGCTCGAAGGCTTGCTGGGCAACGTCAAGAAATATGAGCTGTGGGTGCTCGGTGCCCTGCTCCTGATCGGCCTGTGCCTGTGGGCCCGACGCC | p.Leu145_His146insSerLeuTrpArgArgAlaArgArgLeuAlaGlyGlnArgGlnGluIleTerAlaValGlyAlaArgCysProAlaProAspArgProValProValGlyProThrPro |
| EPN2 | 19 | 34721248 | G>GT | 0/1 | 0/0 | 0/0 | 0/0 | 0/0 | ENSBTAT00000023644.5 | c.1674_1675insA | p.Pro559fs |
| EPN2 | 19 | 34721250 | T>TTGAACACATTCAACGTGCACGCGACAACCAGCAAGGCGTGTTGTTAATCGGTGTCCACTTCCTCACCCTCGAGCTTGGTGCGCGTATCTTTGGTATCCATAATCCGGGTGTCGGCGTCTACCGACC | 0/1 | 0/0 | 0/0 | 0/0 | 0/0 | ENSBTAT00000023644.5 | c.1672_1673insGGTCGGTAGACGCCGACACCCGGATTATGGATACCAAAGATACGCGCACCAAGCTCGAGGGTGAGGAAGTGGACACCGATTAACAACACGCCTTGCTGGTTGTCGCGTGCACGTTGAATGTGTTCA | p.Gln558delinsArgSerValAspAlaAspThrArgIleMetAspThrLysAspThrArgThrLysLeuGluGlyGluGluValAspThrAspTerGlnHisAlaLeuLeuValValAlaCysThrLeuAsnValPheLys |
| ITGA3 | 19 | 37230861 | GC>G | 0/1 | 0/0 | 0/0 | 0/0 | 0/0 | ENSBTAT00000025974.4 | c.94delG | p.Ala32fs |
| ERBB2 | 19 | 40730566 | C>CCGTGGCGGGATCGACGTCAAGGTCACCCATATCGACGCTGGCTACGACGTGGCCG | 0/1 | 0/0 | 0/0 | 0/0 | 0/0 | ENSBTAT00000029056.5 | c.363_364insCGTGGCGGGATCGACGTCAAGGTCACCCATATCGACGCTGGCTACGACGTGGCCG | p.Asn122fs |
| TOP2A | 19 | 41299691 | TA>T | 0/1 | 0/0 | 0/0 | 0/0 | 0/0 | ENSBTAT00000061367.2 | c.1458delT | p.Arg487fs |
| SMARCE1 | 19 | 41491926 | C>CGCAGCGAACA | 0/1 | 0/0 | 0/0 | 0/0 | 0/0 | ENSBTAT00000020356.5 | c.937_938insTGTTCGCTGC | p.Arg313fs |
| SMARCE1 | 19 | 41491929 | T>TGCTGAA | 0/1 | 0/0 | 0/0 | 0/0 | 0/0 | ENSBTAT00000020356.5 | c.934_935insTTCAGC | p.Glu312delinsValGlnGln |
| SMARCE1 | 19 | 41491934 | T>TATA | 0/1 | 0/0 | 0/0 | 0/0 | 0/0 | ENSBTAT00000020356.5 | c.929_930insTAT | p.Gln310delinsHisIle |
| SMARCE1 | 19 | 41491935 | TGC>T | 0/1 | 0/0 | 0/0 | 0/0 | 0/0 | ENSBTAT00000020356.5 | c.927_928delGC | p.Gln310fs |
| **Gene** | **BTA** | **Position** | **Base change** | **Genotype** | | | | | **Transcript** | **cDNA** | **Protein** |
|  |  |  |  | **Case** | **Sire** | **Dam** | **a** | **b** |  |  |  |
| SMARCE1 | 19 | 41491943 | T>TG | 0/1 | 0/0 | 0/0 | 0/0 | 0/0 | ENSBTAT00000020356.5 | c.920dupC | p.Ala308fs |
| SMARCE1 | 19 | 41491947 | T>G | 0/1 | 0/0 | 0/0 | 0/0 | 0/0 | ENSBTAT00000020356.5 | c.917A>C | p.Glu306Ala |
| SMARCE1 | 19 | 41491973 | TCG>T | 0/1 | 0/0 | 0/0 | 0/0 | 0/0 | ENSBTAT00000020356.5 | c.889_890delCG | p.Arg297fs |
| ENSBTAG00000024839 | 19 | 41948100 | A>G | 0/1 | 0/0 | 0/0 | 0/0 | 0/0 | ENSBTAT00000023872.5 | c.173A>G | p.Lys58Arg |
| CNTNAP1 | 19 | 43386192 | GC>G | 0/1 | 0/0 | 0/0 | 0/0 | 0/0 | ENSBTAT00000025424.5 | c.1489delC | p.His497fs |
| CNTNAP1 | 19 | 43386195 | A>T | 0/1 | 0/0 | 0/0 | 0/0 | 0/0 | ENSBTAT00000025424.5 | c.1490A>T | p.His497Leu |
| VAT1 | 19 | 43694416 | C>CTGATGTGGTGGGCATGCGCGATGAACAGCGGGATCA | 0/1 | 0/0 | 0/0 | 0/0 | 0/0 | ENSBTAT00000009715.5 | c.367_368insTGATCCCGCTGTTCATCGCGCATGCCCACCACATCA | p.Gly123delinsValIleProLeuPheIleAlaHisAlaHisHisIleSer |
| MAP3K3 | 19 | 48616073 | G>GGCGCTCCAACTGGCGCTCCAACTCTTCGCGCAGGTCGGTGAGGCGGGCGAGGTT | 0/1 | 0/0 | 0/0 | 0/0 | 0/0 | ENSBTAT00000010716.5 | c.981_982insGCGCTCCAACTGGCGCTCCAACTCTTCGCGCAGGTCGGTGAGGCGGGCGAGGTT | p.Gln327_Tyr328insAlaLeuGlnLeuAlaLeuGlnLeuPheAlaGlnValGlyGluAlaGlyGluVal |
| MAP3K3 | 19 | 48616075 | A>T | 0/1 | 0/0 | 0/0 | 0/0 | 0/0 | ENSBTAT00000010716.5 | c.983A>T | p.Tyr328Phe |
| ENSBTAG00000039765 | 19 | 50880208 | A>T | 0/1 | 0/0 | 0/0 | 0/0 | 0/0 | ENSBTAT00000054772.2 | c.463A>T | p.Asn155Tyr |
| CEP131 | 19 | 52111075 | C>T | 0/1 | 0/0 | 0/0 | 0/0 | 0/0 | ENSBTAT00000001166.5 | c.131C>T | p.Ser44Phe |
| GAA | 19 | 53104404 | GC>G | 0/1 | 0/0 | 0/0 | 0/0 | 0/0 | ENSBTAT00000021325.5 | c.2066delG | p.Arg689fs |
| GAA | 19 | 53104406 | G>A | 0/1 | 0/0 | 0/0 | 0/0 | 0/0 | ENSBTAT00000021325.5 | c.2065C>T | p.Arg689Cys |
| RBFOX3 | 19 | 53941435 | C>CTGTTGTGAGTGTTGAGGTAAAGCTCGTAGTTGACCTACCATTTTTAATGGAGCATCACAGGCAATTGCGAAAGATCA | 0/1 | 0/0 | 0/0 | 0/0 | 0/0 | ENSBTAT00000008240.5 | c.732_733insTGTTGTGAGTGTTGAGGTAAAGCTCGTAGTTGACCTACCATTTTTAATGGAGCATCACAGGCAATTGCGAAAGATCA | p.Thr245fs |
| ENGASE | 19 | 53957137 | TCTC>T | 0/1 | 0/0 | 0/0 | 0/0 | 0/0 | ENSBTAT00000008236.5 | c.1759_1761delGAG | p.Glu587del |
| CD300A | 19 | 57621221 | C>T | 0/1 | 0/0 | 0/0 | 0/0 | 0/0 | ENSBTAT00000040026.4 | c.17G>A | p.Arg6Gln |
| SREK1 | 20 | 13416642 | A>ACCGCTGG | 0/1 | 0/0 | 0/0 | 0/0 | 0/0 | ENSBTAT00000009630.5 | c.685_686insCCAGCGG | p.Val229fs |
| SREK1 | 20 | 13416645 | G>GCCCAGATAATAACCGCAGCCGCCGCCACGATGCCGACAATGCGTCCGCCCATCGGGCCGCGTGGTCCCTGAGAGCTGCTACCGCTCCC | 0/1 | 0/0 | 0/0 | 0/0 | 0/0 | ENSBTAT00000009630.5 | c.682_683insGGGAGCGGTAGCAGCTCTCAGGGACCACGCGGCCCGATGGGCGGACGCATTGTCGGCATCGTGGCGGCGGCTGCGGTTATTATCTGGG | p.Ala228fs |
| MAP3K1 | 20 | 22368640 | C>CTCCACCGACTGGTGCGTTTCCCAGGGCCAGCTCGGCGGCGAACAGAAGGTGCAGCATGTTGATAAGCCCCGGTGGCAGGGAAAAACGGCGTTTAAAGATACCGTT | 0/1 | 0/0 | 0/0 | 0/0 | 0/0 | ENSBTAT00000018325.3 | c.2759_2760insAACGGTATCTTTAAACGCCGTTTTTCCCTGCCACCGGGGCTTATCAACATGCTGCACCTTCTGTTCGCCGCCGAGCTGGCCCTGGGAAACGCACCAGTCGGTGGA | p.Leu920_Ser921insThrValSerLeuAsnAlaValPheProCysHisArgGlyLeuSerThrCysCysThrPheCysSerProProSerTrpProTrpGluThrHisGlnSerValGlu |
| **Gene** | **BTA** | **Position** | **Base change** | **Genotype** | | | | | **Transcript** | **cDNA** | **Protein** |
|  |  |  |  | **Case** | **Sire** | **Dam** | **a** | **b** |  |  |  |
| MCIDAS | 20 | 23937346 | AG>A | 0/1 | 0/0 | 0/0 | 0/0 | 0/0 | ENSBTAT00000064796.1 | c.1064delG | p.Gly355fs |
| C6 | 20 | 33382720 | T>A | 0/1 | 0/0 | 0/0 | 0/0 | 0/0 | ENSBTAT00000018845.4 | c.1987T>A | p.Ser663Thr |
| C7 | 20 | 33561340 | C>CCACCGAGGATATCACGAGCCGTGTTCTCTACCGGTCCACGCAGCGGCAACTTAACGGTTTTGCCTTCCGCAGCGCGATACTGCGCAACGCCGCCAACGTGGCGCGTCATGGCGG | 0/1 | 0/0 | 0/0 | 0/0 | 0/0 | ENSBTAT00000056663.1 | c.1887_1888insCCGCCATGACGCGCCACGTTGGCGGCGTTGCGCAGTATCGCGCTGCGGAAGGCAAAACCGTTAAGTTGCCGCTGCGTGGACCGGTAGAGAACACGGCTCGTGATATCCTCGGTG | p.Ile629_Ala630insProProTerArgAlaThrLeuAlaAlaLeuArgSerIleAlaLeuArgLysAlaLysProLeuSerCysArgCysValAspArgTerArgThrArgLeuValIleSerSerVal |
| DROSHA | 20 | 42072138 | CTG>C | 0/1 | 0/0 | 0/0 | 0/0 | 0/0 | ENSBTAT00000023346.5 | c.1010_1011delTG | p.Val337fs |
| ENSBTAG00000005491 | 20 | 55752688 | G>C | 0/1 | 0/0 | 0/0 | 0/0 | 0/0 | ENSBTAT00000007219.4 | c.553C>G | p.Leu185Val |
| PAPD7 | 20 | 66570731 | AGCATC>A | 0/1 | 0/0 | 0/0 | 0/0 | 0/0 | ENSBTAT00000004384.4 | c.1625_*3delGATGC | p.Ter542fs |
| PAPD7 | 20 | 66570745 | A>AACAGACG | 0/1 | 0/0 | 0/0 | 0/0 | 0/0 | ENSBTAT00000004384.4 | c.1615_1616insCGTCTGT | p.Leu539fs |
| PAPD7 | 20 | 66570748 | C>CG | 0/1 | 0/0 | 0/0 | 0/0 | 0/0 | ENSBTAT00000004384.4 | c.1612_1613insC | p.Ser538fs |
| PAPD7 | 20 | 66570750 | GAC>G | 0/1 | 0/0 | 0/0 | 0/0 | 0/0 | ENSBTAT00000004384.4 | c.1609_1610delGT | p.Val537fs |
| PAPD7 | 20 | 66570756 | C>CGGGTTT | 0/1 | 0/0 | 0/0 | 0/0 | 0/0 | ENSBTAT00000004384.4 | c.1604_1605insAAACCC | p.Leu535_Pro536insAsnPro |
| PAPD7 | 20 | 66570757 | A>AAAT | 0/1 | 0/0 | 0/0 | 0/0 | 0/0 | ENSBTAT00000004384.4 | c.1603_1604insATT | p.Ser534_Leu535insHis |
| PAPD7 | 20 | 66570762 | G>GCGGAAA | 0/1 | 0/0 | 0/0 | 0/0 | 0/0 | ENSBTAT00000004384.4 | c.1598_1599insTTTCCG | p.Asp533_Ser534insPheArg |
| PAPD7 | 20 | 66570763 | T>TCAGGACGCG | 0/1 | 0/0 | 0/0 | 0/0 | 0/0 | ENSBTAT00000004384.4 | c.1597_1598insCGCGTCCTG | p.Arg532_Asp533insAlaArgPro |
| PAPD7 | 20 | 66570767 | T>TTCCCCCA | 0/1 | 0/0 | 0/0 | 0/0 | 0/0 | ENSBTAT00000004384.4 | c.1593_1594insTGGGGGA | p.Arg532fs |
| PAPD7 | 20 | 66570769 | GT>G | 0/1 | 0/0 | 0/0 | 0/0 | 0/0 | ENSBTAT00000004384.4 | c.1591delA | p.Thr531fs |
| ENSBTAG00000038494 | 21 | 20357686 | TG>T | 0/1 | 0/0 | 0/0 | 0/0 | 0/0 | ENSBTAT00000052975.2 | c.439delC | p.Gln147fs |
| BNC1 | 21 | 25248000 | T>TCACAGGCTGG | 0/1 | 0/0 | 0/0 | 0/0 | 0/0 | ENSBTAT00000020545.5 | c.748_749insCACAGGCTGG | p.Phe250fs |
| BNC1 | 21 | 25248002 | C>CACTCAGCGCCCCCTTGGGGCCTGTGGGCG | 0/1 | 0/0 | 0/0 | 0/0 | 0/0 | ENSBTAT00000020545.5 | c.750_751insACTCAGCGCCCCCTTGGGGCCTGTGGGCG | p.Glu251fs |
| BNC1 | 21 | 25248004 | A>ACCCGGGCCCCT | 0/1 | 0/0 | 0/0 | 0/0 | 0/0 | ENSBTAT00000020545.5 | c.752_753insCCCGGGCCCCT | p.Glu251fs |
| BNC1 | 21 | 25248006 | A>ACTCTGAGGCGCTCCCACCCGGCGGGACCCTGGGCCCCTCCGCGC | 0/1 | 0/0 | 0/0 | 0/0 | 0/0 | ENSBTAT00000020545.5 | c.754_755insCTCTGAGGCGCTCCCACCCGGCGGGACCCTGGGCCCCTCCGCGC | p.Asn252fs |
| TRPM1 | 21 | 28036842 | A>T | 0/1 | 0/0 | 0/0 | 0/0 | 0/0 | ENSBTAT00000010233.5 | c.1595T>A | p.Ile532Asn |
| OTUD7A | 21 | 30705912 | GAC>G | 0/1 | 0/0 | 0/0 | 0/0 | 0/0 | ENSBTAT00000009023.4 | c.473_474delAC | p.Asp158fs |
| OTUD7A | 21 | 30705915 | G>T | 0/1 | 0/0 | 0/0 | 0/0 | 0/0 | ENSBTAT00000009023.4 | c.475G>T | p.Gly159Trp |
| **Gene** | **BTA** | **Position** | **Base change** | **Genotype** | | | | | **Transcript** | **cDNA** | **Protein** |
|  |  |  |  | **Case** | **Sire** | **Dam** | **a** | **b** |  |  |  |
| IDH3A | 21 | 31076738 | G>GCAGTGGTTCGAAGCCGCAGACCTGATCGTCAAAGGCAT | 0/1 | 0/0 | 0/0 | 0/0 | 0/0 | ENSBTAT00000008177.3 | c.954_955insCAGTGGTTCGAAGCCGCAGACCTGATCGTCAAAGGCAT | p.Gly319fs |
| ISL2 | 21 | 32110991 | CA>C | 0/1 | 0/0 | 0/0 | 0/0 | 0/0 | ENSBTAT00000022147.4 | c.734delA | p.Lys245fs |
| ISL2 | 21 | 32110994 | G>T | 0/1 | 0/0 | 0/0 | 0/0 | 0/0 | ENSBTAT00000022147.4 | c.735G>T | p.Lys245Asn |
| PEAK1 | 21 | 32837211 | TA>T | 0/1 | 0/0 | 0/0 | 0/0 | 0/0 | ENSBTAT00000006782.5 | c.2037delT | p.Ser680fs |
| PML | 21 | 34971132 | TC>T | 0/1 | 0/0 | 0/0 | 0/0 | 0/0 | ENSBTAT00000020952.5 | c.2436delG | p.Lys813fs |
| PSMA6 | 21 | 45991370 | T>C | 0/1 | 0/0 | 0/0 | 0/0 | 0/0 | ENSBTAT00000012773.4 | c.110T>C | p.Leu37Pro |
| PSMA6 | 21 | 45991372 | A>T | 0/1 | 0/0 | 0/0 | 0/0 | 0/0 | ENSBTAT00000012773.4 | c.112A>T | p.Thr38Ser |
| ODC1 | 21 | 50323275 | T>A | 0/1 | 0/0 | 0/0 | 0/0 | 0/0 | ENSBTAT00000023840.4 | c.908A>T | p.Asn303Ile |
| LCMT2 | 21 | 55602343 | G>T | 0/1 | 0/0 | 0/0 | 0/0 | 0/0 | ENSBTAT00000019239.3 | c.645C>A | p.Phe215Leu |
| MAP1A | 21 | 55826420 | G>GCGGCTGCGTCAGCACATCCGGCAGCAGGTACGTCCGGAGATCAATTTGCGCCAGCGCGAAA | 0/1 | 0/0 | 0/0 | 0/0 | 0/0 | ENSBTAT00000063231.1 | c.5874_5875insCGGCTGCGTCAGCACATCCGGCAGCAGGTACGTCCGGAGATCAATTTGCGCCAGCGCGAAA | p.Glu1960fs |
| C14orf159 | 21 | 56534598 | TCTG>T | 0/1 | 0/0 | 0/0 | 0/0 | 0/0 | ENSBTAT00000061238.2 | c.91_93delCTG | p.Leu31del |
| CCDC88C | 21 | 56643230 | T>TCGTCC | 0/1 | 0/0 | 0/0 | 0/0 | 0/0 | ENSBTAT00000061454.2 | c.4602_4603insGGACG | p.Thr1535fs |
| CCDC88C | 21 | 56643233 | T>TTCCTGATTCTGAACCTGTTTATCCGTAAACCGCGTCCGACCGGGTCGGTATCCGGTCTGTTCCTGATTGGCTACGGCCTGTTCCGCATTATCGTGGAGTTCTTCCGCC | 0/1 | 0/0 | 0/0 | 0/0 | 0/0 | ENSBTAT00000061454.2 | c.4599_4600insGGCGGAAGAACTCCACGATAATGCGGAACAGGCCGTAGCCAATCAGGAACAGACCGGATACCGACCCGGTCGGACGCGGTTTACGGATAAACAGGTTCAGAATCAGGA | p.Thr1533_Thr1534insGlyGlyArgThrProArgTerCysGlyThrGlyArgSerGlnSerGlyThrAspArgIleProThrArgSerAspAlaValTyrGlyTerThrGlySerGluSerGly |
| ENSBTAG00000007041 | 21 | 60996489 | TC>T | 0/1 | 0/0 | 0/0 | 0/0 | 0/0 | ENSBTAT00000009261.4 | c.501delC | p.Phe167fs |
| DYNC1H1 | 21 | 68535444 | A>AGTCCATATGTCGCAGTGCCAGCCCGACGGCGGGAGCAAAAGCGCCGGGGCTGACCGGTAGCGGTGCAGATAACTGACCGATCGCGCTCAGCGGCGACCAGCTTTGCAGTGACGG | 0/1 | 0/0 | 0/0 | 0/0 | 0/0 | ENSBTAT00000044652.2 | c.4714_4715insGTCCATATGTCGCAGTGCCAGCCCGACGGCGGGAGCAAAAGCGCCGGGGCTGACCGGTAGCGGTGCAGATAACTGACCGATCGCGCTCAGCGGCGACCAGCTTTGCAGTGACGG | p.Thr1572delinsSerProTyrValAlaValProAlaArgArgArgGluGlnLysArgArgGlyTerProValAlaValGlnIleThrAspArgSerArgSerAlaAlaThrSerPheAlaValThrAla |
| EOMES | 22 | 2114203 | GT>G | 0/1 | 0/0 | 0/0 | 0/0 | 0/0 | ENSBTAT00000061448.2 | c.1185delA | p.Arg395fs |
| SCN5A | 22 | 12056470 | GT>G | 0/1 | 0/0 | 0/0 | 0/0 | 0/0 | ENSBTAT00000012068.5 | c.2795delA | p.Asn932fs |
| OXTR | 22 | 17818295 | G>GAGTGCCTGACGGATAATGGCGTGC | 0/1 | 0/0 | 0/0 | 0/0 | 0/0 | ENSBTAT00000026346.4 | c.516_517insAGTGCCTGACGGATAATGGCGTGC | p.Val172_His173insSerAlaTerArgIleMetAlaCys |
| **Gene** | **BTA** | **Position** | **Base change** | **Genotype** | | | | | **Transcript** | **cDNA** | **Protein** |
|  |  |  |  | **Case** | **Sire** | **Dam** | **a** | **b** |  |  |  |
| OXTR | 22 | 17818297 | A>ACGCCTCGGGTGACGGTTGCGGGCCGTGCTGTGCCAGTCCCGCCAGCAGCCCGGCAACAAACG | 0/1 | 0/0 | 0/0 | 0/0 | 0/0 | ENSBTAT00000026346.4 | c.519_520insGCCTCGGGTGACGGTTGCGGGCCGTGCTGTGCCAGTCCCGCCAGCAGCCCGGCAACAAACGC | p.Ile174fs |
| CNTN6 | 22 | 25081067 | TG>T | 0/1 | 0/0 | 0/0 | 0/0 | 0/0 | ENSBTAT00000004672.4 | c.1782delC | p.Ser595fs |
| LRIG1 | 22 | 35179503 | C>T | 0/1 | 0/0 | 0/0 | 0/0 | 0/0 | ENSBTAT00000030750.4 | c.2546C>T | p.Thr849Met |
| TKT | 22 | 48264669 | C>CTGATCAGCATTGTAGAAATGCTGCAGCTGGCGCTCGACGACATCCCCCGTCACACCTTCCATAGCCCCTGCAATATTGT | 0/1 | 0/0 | 0/0 | 0/0 | 0/0 | ENSBTAT00000004892.4 | c.29_30insTTGTAGAAATGCTGCAGCTGGCGCTCGACGACATCCCCCGTCACACCTTCCATAGCCCCTGCAATATTGTTGATCAGCA | p.Gln10fs |
| PARP3 | 22 | 49539549 | T>TCTGGCTCATA | 0/1 | 0/0 | 0/0 | 0/0 | 0/0 | ENSBTAT00000045795.3 | c.1218_1219insTATGAGCCAG | p.Ile407fs |
| PARP3 | 22 | 49539551 | T>TGGAGTAAGCGAGTACGCGTTTGATGTCGGTCTGAACCAGCGCGGCAAAGCCTGCCAGCACCAGCGTCACCGCCCCGACGATACCGACCA | 0/1 | 0/0 | 0/0 | 0/0 | 0/0 | ENSBTAT00000045795.3 | c.1216_1217insTGGTCGGTATCGTCGGGGCGGTGACGCTGGTGCTGGCAGGCTTTGCCGCGCTGGTTCAGACCGACATCAAACGCGTACTCGCTTACTCC | p.His406fs |
| CDHR4 | 22 | 51008383 | TC>T | 0/1 | 0/0 | 0/0 | 0/0 | 0/0 | ENSBTAT00000007530.5 | c.847delC | p.Arg283fs |
| CDHR4 | 22 | 51008386 | G>T | 0/1 | 0/0 | 0/0 | 0/0 | 0/0 | ENSBTAT00000007530.5 | c.848G>T | p.Arg283Leu |
| PLXNB1 | 22 | 52026993 | A>G | 0/1 | 0/0 | 0/0 | 0/0 | 0/0 | ENSBTAT00000017840.5 | c.5276A>G | p.His1759Arg |
| PLXNB1 | 22 | 52026994 | C>CTCGCGCCTGAATGATGATCGATATGCCCATGGGCAATGGCGGCCCGCCCGGCTGTCATTGCT | 0/1 | 0/0 | 0/0 | 0/0 | 0/0 | ENSBTAT00000017840.5 | c.5277_5278insTCGCGCCTGAATGATGATCGATATGCCCATGGGCAATGGCGGCCCGCCCGGCTGTCATTGCT | p.Gly1760fs |
| ENSBTAG00000016153 | 22 | 52156998 | C>T | 0/1 | 0/0 | 0/0 | 0/0 | 0/0 | ENSBTAT00000021503.5 | c.4G>A | p.Glu2Lys |
| PLXND1 | 22 | 56811192 | CT>C | 0/1 | 0/0 | 0/0 | 0/0 | 0/0 | ENSBTAT00000002375.5 | c.3248delT | p.Leu1083fs |
| PLXND1 | 22 | 56811198 | G>C | 0/1 | 0/0 | 0/0 | 0/0 | 0/0 | ENSBTAT00000002375.5 | c.3253G>C | p.Ala1085Pro |
| PRIM2 | 23 | 2805865 | T>A | 0/1 | 0/0 | 0/0 | 0/0 | 0/0 | ENSBTAT00000010434.5 | c.162A>T | p.Lys54Asn |
| VPS52 | 23 | 7371914 | A>ATCATGCTGCGCGAAGGGATTGAGGCCGCACTTATCGTCGGCATCATTGCC | 0/1 | 0/0 | 0/0 | 0/0 | 0/0 | ENSBTAT00000003427.5 | c.1999_2000insGGCAATGATGCCGACGATAAGTGCGGCCTCAATCCCTTCGCGCAGCATGA | p.Phe667fs |
| VPS52 | 23 | 7371918 | T>TATCTCAAGCAAACA | 0/1 | 0/0 | 0/0 | 0/0 | 0/0 | ENSBTAT00000003427.5 | c.1995_1996insTGTTTGCTTGAGAT | p.Asn666fs |
| VPS52 | 23 | 7371920 | G>GCCGCGGGCAGTGGA | 0/1 | 0/0 | 0/0 | 0/0 | 0/0 | ENSBTAT00000003427.5 | c.1993_1994insTCCACTGCCCGCGG | p.Thr665fs |
| **Gene** | **BTA** | **Position** | **Base change** | **Genotype** | | | | | **Transcript** | **cDNA** | **Protein** |
|  |  |  |  | **Case** | **Sire** | **Dam** | **a** | **b** |  |  |  |
| ITPR3 | 23 | 7718412 | C>CATACGCGCGGATCGTAGTATTTCTTGTTCGGCTGGTCTTCGCCTTTCGGGTTGCCCAGCTGGCCCTG | 0/1 | 0/0 | 0/0 | 0/0 | 0/0 | ENSBTAT00000053490.2 | c.291_292insATACGCGCGGATCGTAGTATTTCTTGTTCGGCTGGTCTTCGCCTTTCGGGTTGCCCAGCTGGCCCTG | p.Gln98fs |
| IP6K3 | 23 | 7797422 | AT>A | 0/1 | 0/0 | 0/0 | 0/0 | 0/0 | ENSBTAT00000047737.2 | c.1036delA | p.Ile346fs |
| IP6K3 | 23 | 7797425 | A>G | 0/1 | 0/0 | 0/0 | 0/0 | 0/0 | ENSBTAT00000047737.2 | c.1034T>C | p.Met345Thr |
| IP6K3 | 23 | 7797432 | C>T | 0/1 | 0/0 | 0/0 | 0/0 | 0/0 | ENSBTAT00000047737.2 | c.1027G>A | p.Val343Ile |
| IP6K3 | 23 | 7797433 | G>GA | 0/1 | 0/0 | 0/0 | 0/0 | 0/0 | ENSBTAT00000047737.2 | c.1025_1026insT | p.Val343fs |
| ARMC12 | 23 | 9684049 | C>CGCAGAGTCGCCGGATTATCGGCACCACGGTGTTCAGCGGCATGCTGGTGGCGACGGCGATCGGGATTGTGTTTATTCCGTCGCTGTTCGTGCTGTTCCAG | 0/1 | 0/0 | 0/0 | 0/0 | 0/0 | ENSBTAT00000014449.4 | c.83_84insGCAGAGTCGCCGGATTATCGGCACCACGGTGTTCAGCGGCATGCTGGTGGCGACGGCGATCGGGATTGTGTTTATTCCGTCGCTGTTCGTGCTGTTCCAG | p.Val29fs |
| TREML1 | 23 | 15081866 | C>CCAGGCGCGCAAGTCACCGCCAGGGCGTTTATCGGCCACGCTGCCAAGGGCTCGCTCCAGGCAGTTTTGCACCAGG | 0/1 | 0/0 | 0/0 | 0/0 | 0/0 | ENSBTAT00000008500.3 | c.948_*1insCCTGGTGCAAAACTGCCTGGAGCGAGCCCTTGGCAGCGTGGCCGATAAACGCCCTGGCGGTGACTTGCGCGCCTG |  |
| CUL7 | 23 | 16652961 | TC>T | 0/1 | 0/0 | 0/0 | 0/0 | 0/0 | ENSBTAT00000061262.2 | c.1288delG | p.Glu430fs |
| CUL9 | 23 | 16786206 | T>C | 0/1 | 0/0 | 0/0 | 0/0 | 0/0 | ENSBTAT00000026522.4 | c.5798T>C | p.Leu1933Pro |
| ANKRD66 | 23 | 19956716 | A>G | 0/1 | 0/0 | 0/0 | 0/0 | 0/0 | ENSBTAT00000064235.1 | c.140A>G | p.Asn47Ser |
| ADGRF5 | 23 | 20059407 | TG>T | 0/1 | 0/0 | 0/0 | 0/0 | 0/0 | ENSBTAT00000035930.4 | c.3366delC | p.Ile1123fs |
| ENSBTAG00000007075 | 23 | 27842903 | C>G | 0/1 | 0/0 | 0/0 | 0/0 | 0/0 | ENSBTAT00000009299.5 | c.313C>G | p.Leu105Val |
| DDR1 | 23 | 27978505 | A>T | 0/1 | 0/0 | 0/0 | 0/0 | 0/0 | ENSBTAT00000014143.5 | c.1989T>A | p.Asp663Glu |
| DHX16 | 23 | 28147906 | GA>G | 0/1 | 0/0 | 0/0 | 0/0 | 0/0 | ENSBTAT00000009148.3 | c.2018delA | p.Lys673fs |
| DHX16 | 23 | 28147909 | G>T | 0/1 | 0/0 | 0/0 | 0/0 | 0/0 | ENSBTAT00000009148.3 | c.2019G>T | p.Lys673Asn |
| BOLA-NC1 | 23 | 28357859 | G>A | 0/1 | 0/0 | 0/0 | 0/0 | 0/0 | ENSBTAT00000021451.5 | c.155C>T | p.Thr52Met |
| BOLA-NC1 | 23 | 28357860 | T>A | 0/1 | 0/0 | 0/0 | 0/0 | 0/0 | ENSBTAT00000021451.5 | c.154A>T | p.Thr52Ser |
| BOLA-NC1 | 23 | 28357902 | G>C | 0/1 | 0/0 | 0/0 | 0/0 | 0/0 | ENSBTAT00000021451.5 | c.112C>G | p.Leu38Val |
| ENSBTAG00000031843 | 23 | 29223832 | A>T | 0/1 | 0/0 | 0/0 | 0/0 | 0/0 | ENSBTAT00000035615.4 | c.925T>A | p.Phe309Ile |
| ENSBTAG00000000214 | 23 | 29765327 | T>A | 0/1 | 0/0 | 0/0 | 0/0 | 0/0 | ENSBTAT00000025749.5 | c.783T>A | p.His261Gln |
| PSMG4 | 23 | 50330313 | GA>G | 0/1 | 0/0 | 0/0 | 0/0 | 0/0 | ENSBTAT00000044250.3 | c.182delT | p.Ile61fs |
| PSMG4 | 23 | 50330315 | TG>T | 0/1 | 0/0 | 0/0 | 0/0 | 0/0 | ENSBTAT00000044250.3 | c.180delC | p.Ile61fs |
| **Gene** | **BTA** | **Position** | **Base change** | **Genotype** | | | | | **Transcript** | **cDNA** | **Protein** |
|  |  |  |  | **Case** | **Sire** | **Dam** | **a** | **b** |  |  |  |
| DSEL | 24 | 9743480 | G>GAAGGCTGCGCCTAAAGCAGATCCTAAAGCAGCTGAAAAACCAGCTGAAGCTGAAGCAGCGCCAAAAGCAAAC | 0/1 | 0/0 | 0/0 | 0/0 | 0/0 | ENSBTAT00000056960.2 | c.2457_2458insAAGGCTGCGCCTAAAGCAGATCCTAAAGCAGCTGAAAAACCAGCTGAAGCTGAAGCAGCGCCAAAAGCAAAC | p.Val819_Trp820insLysAlaAlaProLysAlaAspProLysAlaAlaGluLysProAlaGluAlaGluAlaAlaProLysAlaAsn |
| MTCL1 | 24 | 41650888 | T>A | 0/1 | 0/0 | 0/0 | 0/0 | 0/0 | ENSBTAT00000065698.1 | c.2561T>A | p.Leu854Gln |
| MTCL1 | 24 | 41650891 | C>CGCT | 0/1 | 0/0 | 0/0 | 0/0 | 0/0 | ENSBTAT00000065698.1 | c.2564_2565insGCT | p.Ser855dup |
| MTCL1 | 24 | 41650894 | C>A | 0/1 | 0/0 | 0/0 | 0/0 | 0/0 | ENSBTAT00000065698.1 | c.2567C>A | p.Pro856Gln |
| MTCL1 | 24 | 41650900 | C>T | 0/1 | 0/0 | 0/0 | 0/0 | 0/0 | ENSBTAT00000065698.1 | c.2573C>T | p.Pro858Leu |
| EPG5 | 24 | 46051808 | T>C | 0/1 | 0/0 | 0/0 | 0/0 | 0/0 | ENSBTAT00000016012.4 | c.7304A>G | p.Asn2435Ser |
| EPG5 | 24 | 46051818 | C>T | 0/1 | 0/0 | 0/0 | 0/0 | 0/0 | ENSBTAT00000016012.4 | c.7294G>A | p.Gly2432Ser |
| EPG5 | 24 | 46051877 | G>A | 0/1 | 0/0 | 0/0 | 0/0 | 0/0 | ENSBTAT00000016012.4 | c.7235C>T | p.Thr2412Met |
| EPG5 | 24 | 46051878 | T>A | 0/1 | 0/0 | 0/0 | 0/0 | 0/0 | ENSBTAT00000016012.4 | c.7234A>T | p.Thr2412Ser |
| EPG5 | 24 | 46051884 | C>T | 0/1 | 0/0 | 0/0 | 0/0 | 0/0 | ENSBTAT00000016012.4 | c.7228G>A | p.Val2410Ile |
| EPG5 | 24 | 46051902 | C>T | 0/1 | 0/0 | 0/0 | 0/0 | 0/0 | ENSBTAT00000016012.4 | c.7210G>A | p.Val2404Ile |
| ALPK2 | 24 | 58160228 | G>GAAAAACACCCTTCGCTGACTGAGAAGATCAATCAACCCGCCGGTACACAGGCAGAACGAAAACAGCACAAACACTTCTTTTGACAGAATTGAC | 0/1 | 0/0 | 0/0 | 0/0 | 0/0 | ENSBTAT00000018977.5 | c.2952_2953insGTCAATTCTGTCAAAAGAAGTGTTTGTGCTGTTTTCGTTCTGCCTGTGTACCGGCGGGTTGATTGATCTTCTCAGTCAGCGAAGGGTGTTTTT | p.Tyr984_Leu985insValAsnSerValLysArgSerValCysAlaValPheValLeuProValTyrArgArgValAspTerSerSerGlnSerAlaLysGlyValPhe |
| STUB1 | 25 | 559224 | G>GCGACCAGTGCATCGTTTTTATTCCAGCCCGTGGCAAAGGCGTTAGCCTCGTATGCCGGGAAAATCCCCACTTCAGGCATCT | 0/1 | 0/0 | 0/0 | 0/0 | 0/0 | ENSBTAT00000025473.5 | c.248_249insCGACCAGTGCATCGTTTTTATTCCAGCCCGTGGCAAAGGCGTTAGCCTCGTATGCCGGGAAAATCCCCACTTCAGGCATCT | p.Cys83_Arg84insAspGlnCysIleValPheIleProAlaArgGlyLysGlyValSerLeuValCysArgGluAsnProHisPheArgHisLeu |
| PKD1 | 25 | 1642207 | C>CGCTGACACCCGCGGCGGTAGTGCGGCTGGCGGAAGCCGCGTACGAAAAATATGGCT | 0/1 | 0/0 | 0/0 | 0/0 | 0/0 | ENSBTAT00000027480.5 | c.6382_6383insAGCCATATTTTTCGTACGCGGCTTCCGCCAGCCGCACTACCGCCGCGGGTGTCAGC | p.Ser2128fs |
| PKD1 | 25 | 1642209 | C>CAATGATTTCAAACTCAAAGGCGGCGTGCTGGCGGGCAGCGAAGA | 0/1 | 0/0 | 0/0 | 0/0 | 0/0 | ENSBTAT00000027480.5 | c.6380_6381insTCTTCGCTGCCCGCCAGCACGCCGCCTTTGAGTTTGAAATCATT | p.Ser2128fs |
| MEFV | 25 | 2692204 | G>A | 0/1 | 0/0 | 0/0 | 0/0 | 0/0 | ENSBTAT00000025458.5 | c.2002C>T | p.Pro668Ser |
| TMC7 | 25 | 17063708 | TC>T | 0/1 | 0/0 | 0/0 | 0/0 | 0/0 | ENSBTAT00000021939.4 | c.915delG | p.Lys306fs |
| ENSBTAG00000046132 | 25 | 22947154 | T>C | 0/1 | 0/0 | 0/0 | 0/0 | 0/0 | ENSBTAT00000062992.1 | c.800T>C | p.Leu267Pro |
| GSG1L | 25 | 25852475 | GC>G | 0/1 | 0/0 | 0/0 | 0/0 | 0/0 | ENSBTAT00000034207.4 | c.20delG | p.Gly7fs |
| GSG1L | 25 | 25852479 | C>CG | 0/1 | 0/0 | 0/0 | 0/0 | 0/0 | ENSBTAT00000034207.4 | c.16dupC | p.Arg6fs |
| **Gene** | **BTA** | **Position** | **Base change** | **Genotype** | | | | | **Transcript** | **cDNA** | **Protein** |
|  |  |  |  | **Case** | **Sire** | **Dam** | **a** | **b** |  |  |  |
| GSG1L | 25 | 25852484 | GCTCGTCTT>G | 0/1 | 0/0 | 0/0 | 0/0 | 0/0 | ENSBTAT00000034207.4 | c.4_11delAAGACGAG | p.Lys2fs |
| ATP2A1 | 25 | 26191461 | T>A | 0/1 | 0/0 | 0/0 | 0/0 | 0/0 | ENSBTAT00000008593.5 | c.1594A>T | p.Thr532Ser |
| KCTD13 | 25 | 26611960 | C>CCCGGA | 0/1 | 0/0 | 0/0 | 0/0 | 0/0 | ENSBTAT00000021258.2 | c.631_632insCCGGA | p.Leu211fs |
| KCTD13 | 25 | 26611965 | C>A | 0/1 | 0/0 | 0/0 | 0/0 | 0/0 | ENSBTAT00000021258.2 | c.636C>A | p.Phe212Leu |
| RABGEF1 | 25 | 28516586 | A>T | 0/1 | 0/0 | 0/0 | 0/0 | 0/0 | ENSBTAT00000005011.5 | c.449A>T | p.His150Leu |
| PLCE1 | 26 | 15669834 | G>GCTTC | 0/1 | 0/0 | 0/0 | 0/0 | 0/0 | ENSBTAT00000025249.5 | c.5336_5337insTTCC | p.Cys1780fs |
| PLCE1 | 26 | 15669838 | G>GAACGTACTTTCTTGGCGAAGAGAAGATAAATTTACTCATGATAAAAA | 0/1 | 0/0 | 0/0 | 0/0 | 0/0 | ENSBTAT00000025249.5 | c.5339_5340insAACGTACTTTCTTGGCGAAGAGAAGATAAATTTACTCATGATAAAAA | p.Cys1780fs |
| PLCE1 | 26 | 15669840 | C>CTCTTTTCGTAGACCATTGAGATGTGAATG | 0/1 | 0/0 | 0/0 | 0/0 | 0/0 | ENSBTAT00000025249.5 | c.5341_5342insTCTTTTCGTAGACCATTGAGATGTGAATG | p.Gln1781fs |
| ALDH18A1 | 26 | 16983694 | C>T | 0/1 | 0/0 | 0/0 | 0/0 | 0/0 | ENSBTAT00000015703.2 | c.298G>A | p.Glu100Lys |
| PI4K2A | 26 | 18684508 | CCG>C | 0/1 | 0/0 | 0/0 | 0/0 | 0/0 | ENSBTAT00000002604.4 | c.297_298delCG | p.Glu100fs |
| PKD2L1 | 26 | 21062138 | GT>G | 0/1 | 0/0 | 0/0 | 0/0 | 0/0 | ENSBTAT00000014222.4 | c.1763delA | p.Tyr588fs |
| ITPRIP | 26 | 25133108 | G>T | 0/1 | 0/0 | 0/0 | 0/0 | 0/0 | ENSBTAT00000005212.4 | c.1636C>A | p.His546Asn |
| ITPRIP | 26 | 25133110 | T>TTTGTCAGTGCCGTATTTGGCGCCGTTACAGCCGTGGGCACGCTGTGGCTGGCCAGCGTTGACGCACAGGCCGGGCTGTTGATCTGG | 0/1 | 0/0 | 0/0 | 0/0 | 0/0 | ENSBTAT00000005212.4 | c.1633_1634insCCAGATCAACAGCCCGGCCTGTGCGTCAACGCTGGCCAGCCACAGCGTGCCCACGGCTGTAACGGCGCCAAATACGGCACTGACAA | p.Asp545fs |
| ENSBTAG00000010522 | 26 | 25715553 | C>CAT | 0/1 | 0/0 | 0/0 | 0/0 | 0/0 | ENSBTAT00000013918.5 | c.2886_2887insAT | p.Gly963fs |
| ENSBTAG00000010522 | 26 | 25715555 | T>G | 0/1 | 0/0 | 0/0 | 0/0 | 0/0 | ENSBTAT00000013918.5 | c.2885A>C | p.Glu962Ala |
| ENSBTAG00000010522 | 26 | 25715558 | T>TTCATACAGGTCCGG | 0/1 | 0/0 | 0/0 | 0/0 | 0/0 | ENSBTAT00000013918.5 | c.2882-1_2882insCCGGACCTGTATGA |  |
| FUOM | 26 | 25875117 | G>GTCGATACGGTTTCCGTTCGACAGCAGGAAA | 0/1 | 0/0 | 0/0 | 0/0 | 0/0 | ENSBTAT00000007505.5 | c.334_335insTCGATACGGTTTCCGTTCGACAGCAGGAAA | p.Gly112delinsValAspThrValSerValArgGlnGlnGluArg |
| FUOM | 26 | 25875121 | T>TAACGGGCTTTATCGGCCACGATGCG | 0/1 | 0/0 | 0/0 | 0/0 | 0/0 | ENSBTAT00000007505.5 | c.338_339insAACGGGCTTTATCGGCCACGATGCG | p.Met113fs |
| FUOM | 26 | 25875124 | T>TCGAGAAACGCGCCAGCACATCC | 0/1 | 0/0 | 0/0 | 0/0 | 0/0 | ENSBTAT00000007505.5 | c.341_342insCGAGAAACGCGCCAGCACATCC | p.Arg116fs |
| FUOM | 26 | 25875126 | G>GACGGTCA | 0/1 | 0/0 | 0/0 | 0/0 | 0/0 | ENSBTAT00000007505.5 | c.344_345insCGGTCAA | p.Glu115fs |
| NKX1-2 | 26 | 44417413 | TC>T | 0/1 | 0/0 | 0/0 | 0/0 | 0/0 | ENSBTAT00000063746.1 | c.316delG | p.Glu106fs |
| C10orf90 | 26 | 46296939 | G>A | 0/1 | 0/0 | 0/0 | 0/0 | 0/0 | ENSBTAT00000032726.3 | c.1544C>T | p.Thr515Met |
| FAT1 | 27 | 15504564 | TG>T | 0/1 | 0/0 | 0/0 | 0/0 | 0/0 | ENSBTAT00000027527.5 | c.6961delC | p.His2321fs |
| **Gene** | **BTA** | **Position** | **Base change** | **Genotype** | | | | | **Transcript** | **cDNA** | **Protein** |
|  |  |  |  | **Case** | **Sire** | **Dam** | **a** | **b** |  |  |  |
| ERI1 | 27 | 24193384 | A>G | 0/1 | 0/0 | 0/0 | 0/0 | 0/0 | ENSBTAT00000012775.2 | c.107A>G | p.Glu36Gly |
| ERI1 | 27 | 24193385 | G>T | 0/1 | 0/0 | 0/0 | 0/0 | 0/0 | ENSBTAT00000012775.2 | c.108G>T | p.Glu36Asp |
| CHRNB3 | 27 | 37180826 | AG>A | 0/1 | 0/0 | 0/0 | 0/0 | 0/0 | ENSBTAT00000039752.3 | c.1138delG | p.Val380fs |
| ENSBTAG00000045608 | 28 | 3274081 | T>A | 0/1 | 0/0 | 0/0 | 0/0 | 0/0 | ENSBTAT00000063767.1 | c.562A>T | p.Lys188* |
| TRIM67 | 28 | 3461613 | G>GCTGGGGGCGATTTTAGGTAAAGAAGACGATCATGTCAGCGAAGCCGACTGCAAAACGGAACCAGAAAAGGTAC | 0/1 | 0/0 | 0/0 | 0/0 | 0/0 | ENSBTAT00000063258.1 | c.2406_2407insGTACCTTTTCTGGTTCCGTTTTGCAGTCGGCTTCGCTGACATGATCGTCTTCTTTACCTAAAATCGCCCCCAG | p.Leu803fs |
| RET | 28 | 13604804 | TA>T | 0/1 | 0/0 | 0/0 | 0/0 | 0/0 | ENSBTAT00000000744.5 | c.2137-2delA |  |
| RET | 28 | 13604806 | G>T | 0/1 | 0/0 | 0/0 | 0/0 | 0/0 | ENSBTAT00000000744.5 | c.2137-1G>T |  |
| RHOBTB1 | 28 | 16744671 | TA>T | 0/1 | 0/0 | 0/0 | 0/0 | 0/0 | ENSBTAT00000025506.5 | c.683delT | p.Leu228fs |
| DNA2 | 28 | 24973978 | G>C | 0/1 | 0/0 | 0/0 | 0/0 | 0/0 | ENSBTAT00000003395.4 | c.2297C>G | p.Ala766Gly |
| USP54 | 28 | 29674834 | T>TTGGCGATATCGTCATTAAAACCAACAACCTGTGCGCGGCAAAAGATGACTGCACCCGTTTGAAAAACGCGCTGGTTAACCTGGGCAATACGCGTAACTG | 0/1 | 0/0 | 0/0 | 0/0 | 0/0 | ENSBTAT00000061411.2 | c.1488_1489insCAGTTACGCGTATTGCCCAGGTTAACCAGCGCGTTTTTCAAACGGGTGCAGTCATCTTTTGCCGCGCACAGGTTGTTGGTTTTAATGACGATATCGCCA | p.Ser496_Lys497insGlnLeuArgValLeuProArgLeuThrSerAlaPhePheLysArgValGlnSerSerPheAlaAlaHisArgLeuLeuValLeuMetThrIleSerPro |
| COMTD1 | 28 | 31171094 | G>GATT | 0/1 | 0/0 | 0/0 | 0/0 | 0/0 | ENSBTAT00000017255.5 | c.758_759insAAT | p.Gly253_Asp254insIle |
| ZMIZ1 | 28 | 35120180 | A>T | 0/1 | 0/0 | 0/0 | 0/0 | 0/0 | ENSBTAT00000025717.5 | c.502A>T | p.Thr168Ser |
| GRID1 | 28 | 40855002 | C>G | 0/1 | 0/0 | 0/0 | 0/0 | 0/0 | ENSBTAT00000026142.4 | c.613G>C | p.Gly205Arg |
| VSTM4 | 28 | 43701317 | G>GTCTGTAACGGCATTGGCTGTCTGATGGTGGGCGCCCTGCTCAACCGCGGCGTCAGGGCGACATGGATCGGGGCCTCAGGCATCGTCCTCACCGGGATTCCCGC | 0/1 | 0/0 | 0/0 | 0/0 | 0/0 | ENSBTAT00000008254.5 | c.775_776insGCGGGAATCCCGGTGAGGACGATGCCTGAGGCCCCGATCCATGTCGCCCTGACGCCGCGGTTGAGCAGGGCGCCCACCATCAGACAGCCAATGCCGTTACAGA | p.Pro259fs |
| ENSBTAG00000009763 | 28 | 44834234 | TA>T | 0/1 | 0/0 | 0/0 | 0/0 | 0/0 | ENSBTAT00000052182.1 | c.349delA | p.Met117fs |
| ENSBTAG00000034891 | 29 | 5669228 | TA>T | 0/1 | 0/0 | 0/0 | 0/0 | 0/0 | ENSBTAT00000049353.2 | c.1053delT | p.Phe351fs |
| ENSBTAG00000046665 | 29 | 27718194 | GAT>G | 0/1 | 0/0 | 0/0 | 0/0 | 0/0 | ENSBTAT00000056729.2 | c.261_262delAT | p.Ser88fs |
| ENSBTAG00000046665 | 29 | 27718198 | T>TAA | 0/1 | 0/0 | 0/0 | 0/0 | 0/0 | ENSBTAT00000056729.2 | c.258_259insTT | p.Thr87fs |
| FOXRED1 | 29 | 29993848 | T>G | 0/1 | 0/0 | 0/0 | 0/0 | 0/0 | ENSBTAT00000030276.3 | c.197T>G | p.Val66Gly |
| AHNAK | 29 | 41589774 | C>T | 0/1 | 0/0 | 0/0 | 0/0 | 0/0 | ENSBTAT00000052103.2 | c.3570G>A | p.Met1190Ile |
| AHNAK | 29 | 41589788 | T>A | 0/1 | 0/0 | 0/0 | 0/0 | 0/0 | ENSBTAT00000052103.2 | c.3556A>T | p.Lys1186* |
| **Gene** | **BTA** | **Position** | **Base change** | **Genotype** | | | | | **Transcript** | **cDNA** | **Protein** |
|  |  |  |  | **Case** | **Sire** | **Dam** | **a** | **b** |  |  |  |
| C11orf86 | 29 | 45625539 | G>A | 0/1 | 0/0 | 0/0 | 0/0 | 0/0 | ENSBTAT00000030041.2 | c.271-1G>A |  |
| C11orf86 | 29 | 45625547 | G>A | 0/1 | 0/0 | 0/0 | 0/0 | 0/0 | ENSBTAT00000030041.2 | c.278G>A | p.Arg93Lys |
| RPS6KB2 | 29 | 45969677 | C>CAACGACGGCCTGCGGGGCTTCGCCCCCCAGCAGACTGAGCAGACCCTGCGTAAAATTTTGCAGACCATTAAGGACGCTAACGCG | 0/1 | 0/0 | 0/0 | 0/0 | 0/0 | ENSBTAT00000012092.4 | c.770_771insAACGACGGCCTGCGGGGCTTCGCCCCCCAGCAGACTGAGCAGACCCTGCGTAAAATTTTGCAGACCATTAAGGACGCTAACGCG | p.Ala257_Leu258insThrThrAlaCysGlyAlaSerProProSerArgLeuSerArgProCysValLysPheCysArgProLeuArgThrLeuThrArg |
| ENSBTAG00000046090 | 29 | 46970069 | C>CGTAACTCCCGCCCACCTCAGTGAACTCCAATCT | 0/1 | 0/0 | 0/0 | 0/0 | 0/0 | ENSBTAT00000063655.1 | c.351_352insAGATTGGAGTTCACTGAGGTGGGCGGGAGTTAC | p.Thr117_Ala118insArgLeuGluPheThrGluValGlyGlySerTyr |
| ENSBTAG00000046090 | 29 | 46970072 | T>TGGGAGCCGGGCTTGC | 0/1 | 0/0 | 0/0 | 0/0 | 0/0 | ENSBTAT00000063655.1 | c.348_349insGCAAGCCCGGCTCCC | p.Leu116_Thr117insAlaSerProAlaPro |
| ENSBTAG00000046090 | 29 | 46970074 | A>C | 0/1 | 0/0 | 0/0 | 0/0 | 0/0 | ENSBTAT00000063655.1 | c.347T>G | p.Leu116Arg |
| CCND1 | 29 | 47544639 | G>GCGGCGACGTTCGACATCATGGTGCTCGATCTTGGCTTGC | 0/1 | 0/0 | 0/0 | 0/0 | 0/0 | ENSBTAT00000023277.5 | c.103_104insCGACGTTCGACATCATGGTGCTCGATCTTGGCTTGCCGG | p.Ala34_Glu35insAlaThrPheAspIleMetValLeuAspLeuGlyLeuPro |
| SHANK2 | 29 | 48230037 | C>T | 0/1 | 0/0 | 0/0 | 0/0 | 0/0 | ENSBTAT00000044310.2 | c.671G>A | p.Arg224His |
| ENSBTAG00000019548 | 30 | 18794052 | A>T | 0/1 | 0/0 | 0/0 | 0/0 | 0/0 | ENSBTAT00000026044.4 | c.121A>T | p.Thr41Ser |
| ENSBTAG00000019548 | 30 | 18794070 | T>TTTCGCAGTTGCGCCACA | 0/1 | 0/0 | 0/0 | 0/0 | 0/0 | ENSBTAT00000026044.4 | c.140_141insTCGCAGTTGCGCCACAT | p.Phe48fs |
| ENSBTAG00000019548 | 30 | 18794074 | T>TTCAGCGGAGGCAGCCCGAAAATGCGCGCATAGTCCCGGTTAAACTGCGGCACACTC | 0/1 | 0/0 | 0/0 | 0/0 | 0/0 | ENSBTAT00000026044.4 | c.143_144insTCAGCGGAGGCAGCCCGAAAATGCGCGCATAGTCCCGGTTAAACTGCGGCACACTC | p.Asp49fs |
| ENSBTAG00000038933 | 30 | 39277840 | C>T | 0/1 | 0/0 | 0/0 | 0/0 | 0/0 | ENSBTAT00000063830.1 | c.913G>A | p.Asp305Asn |
| TAF7L | 30 | 55133054 | T>A | 0/1 | 0/0 | 0/0 | 0/0 | 0/0 | ENSBTAT00000066188.1 | c.989A>T | p.Asn330Ile |
| ENSBTAG00000005319 | 30 | 55602388 | GC>G | 0/1 | 0/0 | 0/0 | 0/0 | 0/0 | ENSBTAT00000006996.5 | c.671delG | p.Gly224fs |
| ENSBTAG00000014287 | 30 | 58419642 | C>T | 0/1 | 0/0 | 0/0 | 0/0 | 0/0 | ENSBTAT00000018979.4 | c.181C>T | p.Arg61Cys |
| CHM | 30 | 74398483 | C>CGCTTGGCGATACCCCAACAATTTTTTACGTCATAAGCATCTTATGCA | 0/1 | 0/0 | 0/0 | 0/0 | 0/0 | ENSBTAT00000012631.5 | c.1778_1779insGCTTGGCGATACCCCAACAATTTTTTACGTCATAAGCATCTTATGCA | p.Glu594fs |
| STARD8 | 30 | 87052688 | G>A | 0/1 | 0/0 | 0/0 | 0/0 | 0/0 | ENSBTAT00000029082.5 | c.2371C>T | p.Pro791Ser |
| STARD8 | 30 | 87052689 | G>GCCACCCACAC | 0/1 | 0/0 | 0/0 | 0/0 | 0/0 | ENSBTAT00000029082.5 | c.2369_2370insGTGTGGGTGG | p.Pro791fs |
| STARD8 | 30 | 87052694 | G>T | 0/1 | 0/0 | 0/0 | 0/0 | 0/0 | ENSBTAT00000029082.5 | c.2365C>A | p.Pro789Thr |
| STARD8 | 30 | 87052697 | G>GA | 0/1 | 0/0 | 0/0 | 0/0 | 0/0 | ENSBTAT00000029082.5 | c.2361_2362insT | p.Pro788fs |
| STARD8 | 30 | 87052699 | CT>C | 0/1 | 0/0 | 0/0 | 0/0 | 0/0 | ENSBTAT00000029082.5 | c.2359delA | p.Ser787fs |
| STARD8 | 30 | 87052703 | G>GC | 0/1 | 0/0 | 0/0 | 0/0 | 0/0 | ENSBTAT00000029082.5 | c.2355_2356insG | p.Leu786fs |
| **Gene** | **BTA** | **Position** | **Base change** | **Genotype** | | | | | **Transcript** | **cDNA** | **Protein** |
|  |  |  |  | **Case** | **Sire** | **Dam** | **a** | **b** |  |  |  |
| STARD8 | 30 | 87052705 | T>TGCTCGCGAAAAACCTGACAACACCG | 0/1 | 0/0 | 0/0 | 0/0 | 0/0 | ENSBTAT00000029082.5 | c.2353_2354insCGGTGTTGTCAGGTTTTTCGCGAGC | p.Glu785fs |
| STARD8 | 30 | 87052708 | G>GGGCTCCAGACACC | 0/1 | 0/0 | 0/0 | 0/0 | 0/0 | ENSBTAT00000029082.5 | c.2350_2351insGGTGTCTGGAGCC | p.Ala784fs |
| STARD8 | 30 | 87052712 | CACT>C | 0/1 | 0/0 | 0/0 | 0/0 | 0/0 | ENSBTAT00000029082.5 | c.2344_2346delAGT | p.Ser782del |
| STARD8 | 30 | 87052717 | T>TCATCGTTC | 0/1 | 0/0 | 0/0 | 0/0 | 0/0 | ENSBTAT00000029082.5 | c.2341_2342insGAACGATG | p.Tyr781fs |
| EDA2R | 30 | 89706166 | C>A | 0/1 | 0/0 | 0/0 | 0/0 | 0/0 | ENSBTAT00000065245.1 | c.355C>A | p.Leu119Ile |
| EDA2R | 30 | 89706169 | G>T | 0/1 | 0/0 | 0/0 | 0/0 | 0/0 | ENSBTAT00000065245.1 | c.358G>T | p.Val120Leu |
| EDA2R | 30 | 89706174 | G>GCCGAACAACATCCCAGGCCGCGCCAGCGCAAGCCTGCGCGA | 0/1 | 0/0 | 0/0 | 0/0 | 0/0 | ENSBTAT00000065245.1 | c.363_364insCCGAACAACATCCCAGGCCGCGCCAGCGCAAGCCTGCGCGA | p.Phe122fs |
| SLC9A7 | 30 | 90235580 | TAA>T | 0/1 | 0/0 | 0/0 | 0/0 | 0/0 | ENSBTAT00000004260.5 | c.785_786delTT | p.Phe262fs |
| FAM120C | 30 | 96944381 | CT>C | 0/1 | 0/0 | 0/0 | 0/0 | 0/0 | ENSBTAT00000005030.4 | c.2985delA | p.Glu996fs |
| FAM120C | 30 | 96944386 | C>A | 0/1 | 0/0 | 0/0 | 0/0 | 0/0 | ENSBTAT00000005030.4 | c.2981G>T | p.Gly994Val |
| GPR82 | 30 | 107358518 | AC>A | 0/1 | 0/0 | 0/0 | 0/0 | 0/0 | ENSBTAT00000064230.1 | c.215delG | p.Gly72fs |
| POLA1 | 30 | 125652004 | A>AGC | 0/1 | 0/0 | 0/0 | 0/0 | 0/0 | ENSBTAT00000010742.4 | c.1053_1054dupGC | p.Leu352fs |
| POLA1 | 30 | 125652008 | A>AGCGTCAGCATGCGTTTAATTACGCGCTGTTCGGTGTGGGTGAATTCACGACCTTCTACTTTGGTCGGGAAACGGCCATCGCCGCCAAACAGGTTATCCACCGCG | 0/1 | 0/0 | 0/0 | 0/0 | 0/0 | ENSBTAT00000010742.4 | c.1050_1051insCGCGGTGGATAACCTGTTTGGCGGCGATGGCCGTTTCCCGACCAAAGTAGAAGGTCGTGAATTCACCCACACCGAACAGCGCGTAATTAAACGCATGCTGACGC | p.Trp351fs |
| PTCHD1 | 30 | 126939699 | G>GAAAA | 0/1 | 0/0 | 0/0 | 0/0 | 0/0 | ENSBTAT00000027948.3 | c.183_184insTTTT | p.Leu62fs |
| PTCHD1 | 30 | 126939702 | T>TTCGCC | 0/1 | 0/0 | 0/0 | 0/0 | 0/0 | ENSBTAT00000027948.3 | c.180_181insGGCGA | p.Ser61fs |
| PTCHD1 | 30 | 126939705 | G>A | 0/1 | 0/0 | 0/0 | 0/0 | 0/0 | ENSBTAT00000027948.3 | c.178C>T | p.His60Tyr |
| PTCHD1 | 30 | 126939709 | G>GTCAC | 0/1 | 0/0 | 0/0 | 0/0 | 0/0 | ENSBTAT00000027948.3 | c.173_174insGTGA | p.Gln59fs |
| PTCHD1 | 30 | 126939710 | G>GAATCGTATTACCCAACA | 0/1 | 0/0 | 0/0 | 0/0 | 0/0 | ENSBTAT00000027948.3 | c.172_173insTGTTGGGTAATACGATT | p.Pro58fs |
| PTCHD1 | 30 | 126939712 | C>CTCA | 0/1 | 0/0 | 0/0 | 0/0 | 0/0 | ENSBTAT00000027948.3 | c.170_171insTGA | p.Ala57_Pro58insGlu |
| CNKSR2 | 30 | 129105653 | A>T | 0/1 | 0/0 | 0/0 | 0/0 | 0/0 | ENSBTAT00000061509.2 | c.308T>A | p.Ile103Lys |
| ARHGAP6 | 30 | 137693623 | A>ATCAGCTTGAGCAACGTGGTTTTACCGGTACCGTTGGCACCCAGCAGACCGATACGGTCGCCGCGCTGCAGCACCATCGAGAAGTCCTTGAGCAG | 0/1 | 0/0 | 0/0 | 0/0 | 0/0 | ENSBTAT00000003400.3 | c.2219_2220insTCAGCTTGAGCAACGTGGTTTTACCGGTACCGTTGGCACCCAGCAGACCGATACGGTCGCCGCGCTGCAGCACCATCGAGAAGTCCTTGAGCAG | p.Gln740fs |
| CD99 | 30 | 140194483 | AAG>A | 0/1 | 0/0 | 0/0 | 0/0 | 0/0 | ENSBTAT00000010674.4 | c.453_454delCT | p.Phe152fs |
