# Supplementary material for: De novo ZIC2 frameshift variant associated with frontonasal dysplasia in a Limousin calf
Source: BMC Genomics. 2021 Jan 2;22:1. doi: 10.1186/s12864-020-07350-y (PMC7777292; doi:10.1186/s12864-020-07350-y)
Supplement: Supplementary file 2 — Additional file 2: Table S2. Candidate genes for frontonasal dysplasia (FND), arhinencephaly, holoprosencephaly and cyclopia in mammalian animals according to NCBI. The bovine orthologues gene were presented with the chromosomal position according to UMD3.1. The candidate gene ZIC2 which was filtered out of whole genome sequencing data is in bold. [file 12864_2020_7350_MOESM2_ESM.docx]

**Table S2** Candidate genes for frontonasal dysplasia (FND), arhinencephaly, holoprosencephaly and cyclopia in mammals according to NCBI. The bovine orthologues gene with their chromosomal position are presented according to UMD3.1. The candidate gene *ZIC2* which was filtered out of whole genome sequencing data is printed in bold.

| **Phenotype** | **Gene** | **Gene full name** | **Species** | **Bovine gene ID** | **Bovine gene** | **BTA** | **Position** |
| --- | --- | --- | --- | --- | --- | --- | --- |
| Cyclopia | *zic1* | zic family member 1 (odd-paired homolog, Drosophila) | Danio rerio | ENSBTAG00000014751 | *ZIC1* | 1 | 121863151-121867967 |
| Holoprosencephaly | *Boc* | biregional cell adhesion molecule-related/down-regulated by oncogenes (Cdon) binding protein | Mus musculus | ENSBTAG00000013909 | *BOC* | 1 | 58428032-58464999 |
| Holoprosencephaly | *TRAPPC10* | trafficking protein particle complex 10 | Homo sapiens | ENSBTAG00000007100 | *TRAPPC10* | 1 | 146785260-146840277 |
| Holoprosencephaly | *ZIC4* | Zic family member 4 | Homo sapiens | ENSBTAG00000014749 | *ZIC4* | 1 | 121874133-121886919 |
| Holoprosencephaly | *GLI2* | GLI family zinc finger 2 | Homo sapiens  Mus musculus | ENSBTAG00000011682 | *GLI2* | 2 | 72977209-73168370 |
| Cyclopia | *gbx2* | gastrulation brain homeobox 2 | Danio rerio | ENSBTAG00000008898 | *GBX2* | 3 | 116266172-116268268 |
| FND | *ALX3* | ALX homeobox 3 | Homo sapiens | ENSBTAG00000020966 | *ALX3* | 3 | 33469022-33478299 |
| Holoprosencephaly | *STIL* | STIL, centriolar assembly protein | Homo sapiens | ENSBTAG00000017844 | *STIL* | 3 | 99537145-99587496 |
| Holoprosencephaly  Arhinencephaly | *SHH* | sonic hedgehog | Homo sapiens  Mus musculus  Gallus gallus | ENSBTAG00000024552 | *SHH* | 4 | 118265405-118274566 |
| Arhinencephaly | Gli3 | GLI-Kruppel family member GLI3 | Mus musculus | ENSBTAG00000010671 | GLI3 | 4 | 79444243-79758476 |
| FND | *ALX1* | ALX homeobox 1 | Homo sapiens  Felis catus | ENSBTAG00000014977 | *ALX1* | 5 | 14991697-15013533 |
| Holoprosencephaly | *KMT2D* | lysine methyltransferase 2D | Homo sapiens | ENSBTAG00000014429 | *KMT2D* | 5 | [30926510-30967026](http://genome.ucsc.edu/cgi-bin/hgTracks?position=chr5:30926510-30967026&hgsid=822937943_mU9H0BuAL3sMyAI5ZtY4KMS5rcHf&xenoRefGene=pack&hgFind.matches=NM_003482,) |
| Holoprosencephaly | *PPP1R12A* | protein phosphatase 1 regulatory subunit 12A | Homo sapiens | ENSBTAG00000014609 | *PPP1R12A* | 5 | [9352198-9511533](http://genome.ucsc.edu/cgi-bin/hgTracks?position=chr5:9352198-9511533&hgsid=822937943_mU9H0BuAL3sMyAI5ZtY4KMS5rcHf&refGene=pack&hgFind.matches=NM_001102217,) |
| Holoprosencephaly | *Rfx4* | regulatory factor X, 4 (influences HLA class II expression) | Mus musculus | ENSBTAG00000025837 | *RFX4* | 5 | 70323198-70395056 |
| Holoprosencephaly | *GAS1* | growth arrest specific 1 | Homo sapiens | ENSBTAG00000046803 | *GAS1* | 8 | 81511017-81512051 |
| Holoprosencephaly | *PTCH1* | patched 1 | Homo sapiens  Mus musculus | ENSBTAG00000048213  ENSBTAG00000009903  ENSBTAG00000048213 | *N/A*  *N/A*  *N/A* | 8  8  8 | 83551682-83581560  83522484-83546361  83551682-83581560 |
| Cyclopia | *ESR1* | estrogen receptor 1 | Homo sapiens | ENSBTAG00000007159 | *ESR1* | 9 | 89969586-90255801 |
| Holoprosencephaly | *DLL1* | delta like canonical Notch ligand 1 | Homo sapiens | ENSBTAG00000031476 | *DLL1* | 9 | 105508313-105516028 |
| Holoprosencephaly | *Bmp4* | bone morphogenetic protein 4 | Mus musculus | ENSBTAG00000003835 | *BMP4* | 10 | 66751296-66755026 |
| FND | *SIX2* | SIX homeobox 2 | Homo sapiens  Mus musculus | ENSBTAG00000004159 | *SIX2* | 11 | 27260475-27263699 |
| Holoprosencephaly | *APOB* | apolipoprotein B | Homo sapiens | ENSBTAG00000008505 | *APOB* | 11 | 77953380-78040118 |
| Holoprosencephaly | *NOTCH1* | notch 1 | Homo sapiens | ENSBTAG00000022799 | *NOTCH1* | 11 | 103986874-104030692 |
| Holoprosencephaly | *SIX3* | SIX homeobox 3 | Homo sapiens  Mus musculus | ENSBTAG00000027017 | *SIX3* | 11 | 27200797-27204850 |
| **Holoprosencephaly** | ***ZIC2*** | **Zic family member 2** | **Homo sapiens**  **Mus musculus** | **ENSBTAG00000025246** | ***ZIC2*** | **12** | **80718632-80724166** |
| Holoprosencephaly | *FOXH1* | forkhead box H1 | Homo sapiens | ENSBTAG00000004761 | *FOXH1* | 14 | 1654701-1656256 |
| Holoprosencephaly | *RAD21* | RAD21 cohesin complex component | Homo sapiens | ENSBTAG00000007303 | *RAD21* | 14 | [49558483-49588329](http://genome.ucsc.edu/cgi-bin/hgTracks?position=chr14:49558483-49588329&hgsid=822937943_mU9H0BuAL3sMyAI5ZtY4KMS5rcHf&refGene=pack&hgFind.matches=NM_001034717,) |
| FND | *ALX4* | ALX homeobox 4 | Homo sapiens | ENSBTAG00000027563 | *ALX4* | 15 | 75154393-75187019 |
| FND | *Pax6* | paired box 6 | Mus musculus | ENSBTAG00000004561 | *PAX6* | 15 | 63356631-63384294 |
| Holoprosencephaly | *APOE* | apolipoprotein E | Homo sapiens | ENSBTAG00000010123 | *APOE* | 15 | 53040105-53042792 |
| Holoprosencephaly | *PRRX1* | paired related homeobox 1 | Homo sapiens | ENSBTAG00000004570 | *PRRX1* | 16 | 39016045-39091099 |
| Holoprosencephaly | *DISP1* | dispatched RND transporter family member 1 | Homo sapiens | ENSBTAG00000049277 | *DISP1* | 16 | [26998680-27209884](http://genome.ucsc.edu/cgi-bin/hgTracks?position=chr16:26998680-27209884&hgsid=822937943_mU9H0BuAL3sMyAI5ZtY4KMS5rcHf&refGene=pack&hgFind.matches=NM_001038161,) |
| Holoprosencephaly | *CNOT1* | CCR4-NOT transcription complex subunit 1 | Homo sapiens | ENSBTAG00000020782 | *CNOT1* | 18 | [26390037-26473032](http://genome.ucsc.edu/cgi-bin/hgTracks?position=chr18:26390037-26473032&hgsid=822937943_mU9H0BuAL3sMyAI5ZtY4KMS5rcHf&refGene=pack&hgFind.matches=NM_001206039,) |
| Holoprosencephaly | *NOG* | noggin | Homo sapiens | ENSBTAG00000040282 | *NOG* | 19 | 7613376-7614074 |
| Holoprosencephaly | *FBXW11* | F-box and WD repeat domain containing 11 | Homo sapiens | ENSBTAG00000015376 | *FBXW11* | 20 | 3584765-3624526 |
| Cyclopia | *Egfr* | Epidermal growth factor receptor | Drosophila melanogaster | ENSBTAG00000011628 | *EGFR* | 22 | 892005-1069280 |
| Holoprosencephaly | *TGIF1* | TGFB induced factor homeobox 1 | Homo sapiens  Mus musculus | ENSBTAG00000007718 | *TGIF1* | 24 | 37940878-37948732 |
| Holoprosencephaly | *TWSG1* | twisted gastrulation BMP signaling modulator 1 | Homo sapiens  Mus musculus | ENSBTAG00000001805 | *TWSG1* | 24 | 41986506-42008147 |
| Holoprosencephaly | *FGF8* | fibroblast growth factor 8 | Homo sapiens | ENSBTAG00000001530 | *FGF8* | 26 | 22375029-22380835 |
| Holoprosencephaly | *SMC3* | structural maintenance of chromosomes 3 | Homo sapiens | ENSBTAG00000013905 | *SMC3* | 26 | [31387437-31425087](http://genome.ucsc.edu/cgi-bin/hgTracks?position=chr26:31387437-31425087&hgsid=822937943_mU9H0BuAL3sMyAI5ZtY4KMS5rcHf&refGene=pack&hgFind.matches=NM_174295,) |
| Holoprosencephaly | *FGFR1* | fibroblast growth factor receptor 1 | Homo sapiens | ENSBTAG00000015457 | *FGFR1* | 27 | 33250534-33291989 |
| Holoprosencephaly | *NODAL* | nodal growth differentiation factor | Homo sapiens  Gallus gallus | ENSBTAG00000013090 | *NODAL* | 28 | 26794899-26801991 |
| Holoprosencephaly | *CDON* | cell adhesion associated, oncogene regulated | Homo sapiens  Mus musculus | ENSBTAG00000009315 | *CDON* | 29 | 29694499-29757830 |
| Holoprosencephaly | *DHCR7* | 7-dehydrocholesterol reductase | Homo sapiens | ENSBTAG00000016465 | *DHCR7* | 29 | 48930324-48950644 |
| Holoprosencephaly | *SMC1A* | structural maintenance of chromosomes 1A | Homo sapiens | ENSBTAG00000017761 | *SMC1A* | X | [96218220-96252806](http://genome.ucsc.edu/cgi-bin/hgTracks?position=chrX:96218220-96252806&hgsid=822937943_mU9H0BuAL3sMyAI5ZtY4KMS5rcHf&refGene=pack&hgFind.matches=NM_174614,) |
| Holoprosencephaly | *STAG2* | stromal antigen 2 | Homo sapiens | ENSBTAG00000009121 | *STAG2* | X | [7890545-8020362](http://genome.ucsc.edu/cgi-bin/hgTracks?position=chrX:7890545-8020362&hgsid=822937943_mU9H0BuAL3sMyAI5ZtY4KMS5rcHf&refGene=pack&hgFind.matches=NM_001354660,) |
| Cyclopia | *dpp* | decapentaplegic | Drosophila melanogaster | N/A | *N/A* | N/A | N/A |
| Holoprosencephaly | *foxa2.L* | forkhead box A2 L homeolog | Xenopus laevis | N/A | *N/A* | N/A | N/A |
| Holoprosencephaly | *Hhat* | hedgehog acyltransferase | Mus musculus | N/A | *N/A* | N/A | N/A |
| Holoprosencephaly | *HPE1* | holoprosencephaly 1, alobar | Homo sapiens | N/A | *N/A* | N/A | N/A |
| Holoprosencephaly | *HPE6* | holoprosencephaly 6 | Homo sapiens | N/A | *N/A* | N/A | N/A |
| Holoprosencephaly | *HPE8* | holoprosencephaly 8 | Homo sapiens | N/A | *N/A* | N/A | N/A |
| Holoprosencephaly | *LOC110008580* | Zic family member 2 polyalanine repeat instability region | Homo sapiens | N/A | *N/A* | N/A | N/A |
| Holoprosencephaly | *SBE2* | SHH brain enhancer 2 | Homo sapiens | N/A | *N/A* | N/A | N/A |
| Holoprosencephaly | *six3a* | SIX homeobox 3a | Danio rerio | N/A | *N/A* | N/A | N/A |
